# Synthesis and Biological Evaluation of New Thiocarbamoylpyrazoline and Chalcone Derivatives on Bone Cancer Cell Lines: *In Vitro* and *In Silico* Studies

**DOI:** 10.1021/acsomega.6c00603

**Published:** 2026-06-05

**Authors:** Fatma Demir, Nuran Kahriman, Ali Aydın, Burçin Türkmenoğlu, Emre Mandal, Safiye Emirdağ

**Affiliations:** † Department of Chemistry, Faculty of Science, 52976 Karadeniz Technical University, Trabzon 61080, Türkiye; ‡ Department of Basic Medical Science, Faculty of Medicine Bozok University, Yozgat 66900, Türkiye; § Department of Analytical Chemistry, Faculty of Pharmacy, 162315 Erzincan Binali Yıldırım University, Erzincan 24002, Türkiye; ∥ Department of Chemistry, Faculty of Science, 37509 Ege University, İzmir 35040, Türkiye

## Abstract

In the pursuit of multifunctional therapeutic agents, a new series of thiocarbamoylpyrazoline derivatives were synthesized starting from chalcones, and all synthesized compounds (**1–12**) were structurally characterized using spectroscopic methods (NMR, FT-IR, and Q-TOF-LC-MS) and elemental analysis. The anticancer properties of the test compounds were evaluated *in vitro* against human bone cancer cell lines MG63 and SW1353 using MTT and LDH assays. LDH assay results demonstrated that the compounds exhibited no detectable cytotoxicity toward normal human chondrocyte (HC) cells. Compared to the reference drug 5-fluorouracil (5-FU), several compounds displayed notable antiproliferative activity. Tumor selectivity index (TSI) analysis identified compounds **1**, **2, 4**, and **10** as having high tumor selectivity, indicating a preferential cytotoxic effect toward cancer cells over normal cells. Among these, compounds **4** and **10** exhibited the most favorable anticancer profiles, combining potent antiproliferative activity with minimal toxicity to normal cells. Furthermore, to support the experimental anticancer findings, *in silico* molecular docking studies were performed using the crystal structures of caspase-3 (1GFW), human metalloproteinase-13 (3KEJ), human estrogen receptor (3ERT), and EGFR (1M17) against compounds **1**, **2**, **4**, and **10**, and their binding modes were analyzed.

## Graphical abstract

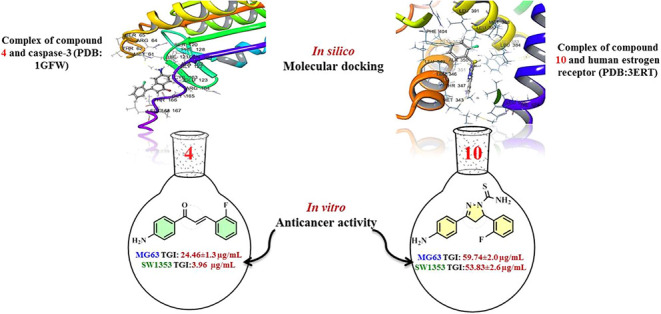

## 1 Introduction

Cancer remains a major global health challenge, despite substantial advances in diagnosis and treatment.^1,2^ Although chemotherapy continues to play a central role in cancer management, its effectiveness is often limited by toxicity to normal cells and the emergence of drug resistance. Therefore, the development of new anticancer agents with improved selectivity and reduced adverse effects remains an important goal.

Chalcones, characterized by the 1,3-diaryl-2-propen-1-one scaffold, are naturally occurring compounds and valuable synthetic intermediates.^3^ Among their diverse biological properties,^4–10^ their anticancer potential has attracted considerable attention.^11–22^ In particular, structural modification of chalcones, including halogen substitution, has been reported to improve biological activity and enhance their relevance in medicinal chemistry (Figure 1).^23–27^

**Figure 1:**
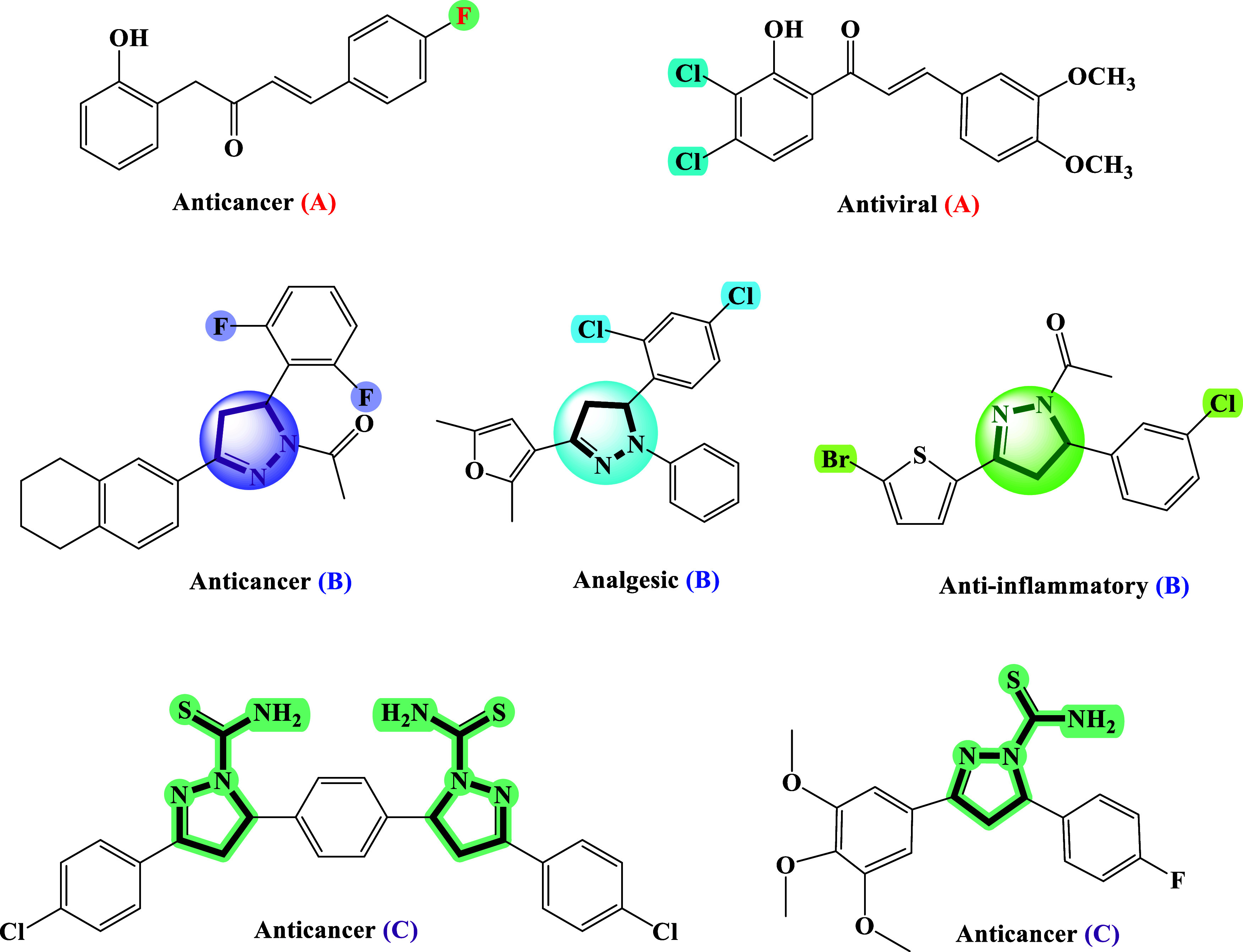
Some bioactive halo-substituted chalcones (A), pyrazoline derivatives (B), and thiocarbamoylpyrazoline compounds (C).

Chalcones also serve as useful precursors for the synthesis of pyrazoline derivatives, an important class of nitrogen-containing heterocycles in medicinal chemistry.^28–30^ Pyrazoline-based compounds have been associated with a variety of biological activities, including anticancer effects,^31–36^ and previous studies have suggested that they may act through several molecular targets such as EGFR-TK, COX-2, B-Raf kinase, aurora kinases, telomerase, and tubulin polymerization (Figure 1).^37,38^

Among these compounds, thiocarbamoylpyrazoline derivatives are of particular interest because they combine a pyrazoline nucleus with a carbothioamide moiety, a structural feature associated with notable pharmacological potential.^39–42^ Selected bioactive halo-substituted chalcones, pyrazoline derivatives, and thiocarbamoylpyrazoline compounds reported in the literature, together with their biological activities, are presented in Figure 1.^39–42^

Driven by the continuing need for new anticancer agents with improved efficacy and reduced side effects, natural product-like systems and heterocyclic scaffolds remain important in medicinal chemistry. In this context, fluorine-substituted aminochalcones and novel thiocarbamoylpyrazoline derivatives were synthesized and evaluated for their anticancer effects against bone cancer cell lines, with the aim of assessing the potential of 1,3,5-trisubstituted Δ^2^-pyrazoline derivatives as promising anticancer candidates.

## 2 Results and Discussion

This study reports the synthesis of six fluoro-substituted aminochalcones (**1–6**) and their corresponding thiocarbamoylpyrazoline derivatives (**7–12**). In the design of both fluoro-substituted aminochalcone and thiocarbamoyl compounds, substituents with high biological activity were preferred.

Amino (−NH_2_) and fluorine substituents were deliberately chosen in the design of the synthesized molecules because of their well-established contributions to biological activity in medicinal chemistry. The −NH_2_ group is a key functional moiety, as it can act as both a hydrogen bond donor and acceptor, enabling substituted compounds to form strong interactions with amino acid residues in enzyme active sites, thereby enhancing binding affinity and inhibitory potency.^43,44^ In addition, its electron-donating character can increase the nucleophilicity of neighboring atoms and stabilize reactive intermediates, further modulating enzyme activity. This dual hydrogen-bonding capacity and electronic influence make the amino group particularly valuable for improving molecular interactions with biological targets through donor–acceptor mechanisms.^43,44^

Similarly, fluorine was incorporated because of its distinctive electronic modulation, hydrogen-bonding potential, and well-known bioisosteric effects in medicinal chemistry. Fluorine’s strong electronegativity can modulate electronic properties, affect lipophilicity, and improve metabolic stability. Moreover, fluorine can increase binding affinity through inductive effects without significantly increasing steric volume.^45,46^

Collectively, these properties make the −NH_2_ and −F groups widely employed functional groups in rational drug design and enzyme-targeted therapies. For this reason, these two functional groups were selected.

The anticancer activities of the synthesized compounds were systematically evaluated. Compounds **1–6** and **12** have been previously reported in the literature, whereas compounds **7–11** are novel. Importantly, all synthesized compounds (**1–12**) have been evaluated for their anticancer activity against MG63 and SW1353 bone cancer cell lines for the first time in this study.^15,42–54^

### 2.1 Chemistry

The detailed synthetic procedures for the preparation of chalcones (**1–6**) and thiocarbamoylpyrazoline derivatives (**7–12**) are illustrated in Scheme 1. The purity of all synthesized compounds was initially assessed by thin-layer chromatography (TLC). When necessary, further purification was achieved using column chromatography. The identity and purity of the newly synthesized molecules were confirmed by ^1^H NMR, ^13^C-APT NMR, FT-IR spectroscopic characterization, HRMS, and elemental analysis. The ACD NMR program was also used to support data from NMR spectroscopy.^55^

**Scheme 1:**
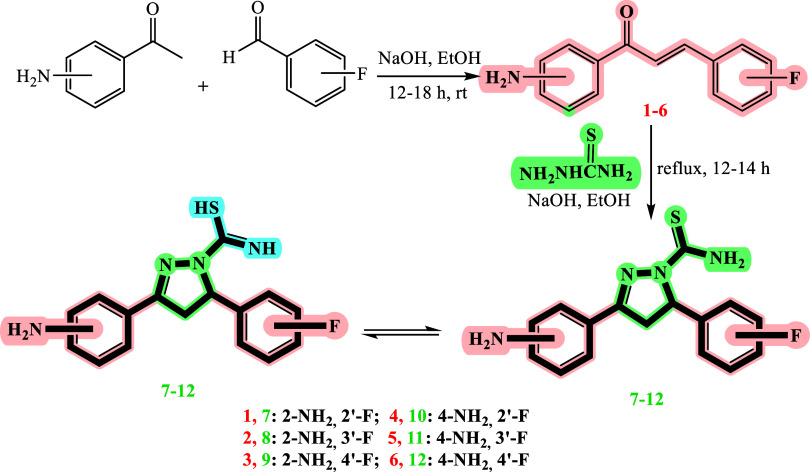
Consecutive Synthesis of Chalcones (**1–6**) and Thiocarbamoylpyrazoline Derivatives (**7–12**)

In the first stage of the study, the appropriate aldehydes and ketones were subjected to Claisen–Schmidt condensation in a basic ethanol/water reaction medium to afford fluoro-substituted aminochalcones.^9–12^

Upon examination of the ^1^H NMR spectra of chalcone derivatives **1–6**, it is observed that the H-2 protons of the α,β-unsaturated carbonyl system resonate in the range of 7.50–7.76 ppm, while the H-3 protons appear in the range of 7.71–7.89 ppm, with a coupling constant of *J* = 16.0 Hz. This coupling constant, which is indicative of a *trans* configuration between the two protons, along with the AB spin system they exhibit, provides strong evidence for the formation of the chalcone skeleton and confirms the successful synthesis of the targeted structures. Similarly, in the ^13^C NMR spectra of the same compounds, the resonance signals of the carbonyl carbon (C-1) are observed in the range of 187.68–191.63 ppm, those of the C-2 carbon in the range of 121.78–124.69 ppm, and those of the C-3 carbon in the range of 135.62–141.86 ppm further support the proposed structures. Analysis of the FT-IR spectra of these compounds reveals characteristic peaks corresponding to asymmetric −NH_2_ stretching vibrations in the range of 3330–3480 cm^–1^ and symmetric −NH_2_ stretching vibrations in the range of 3214–3480 cm^–1^. Additionally, C=O stretching vibrations appear in the range of 1628–1647 cm^–1^, and C=C stretching vibrations are observed between 1569 and 1579 cm^–1^. Mass spectrometric (Q-TOF-LC-MS) analysis further confirmed these results. A molecular ion peak at *m*/*z* 314 was identified for all compounds. In particular, the sodium adduct ion [M + Na]^+^ at *m*/*z* 337 was typically observed as the base peak.

These spectral features confirm the presence of the expected functional groups in chalcones.

Chalcones are highly effective intermediates in the synthesis of bioactive five-membered heterocyclic compounds. In particular, the *α,β*-unsaturated carbonyl system in their structures plays a key role in cyclization reactions. Therefore, in the second part of the synthetic study, novel thiocarbamoylpyrazoline derivatives (**7**–**12**) were synthesized via a Michael addition reaction between thiosemicarbazide and the previously prepared chalcone derivatives.

The structures of the synthesized thiocarbamoylpyrazoline derivatives (compounds **7**–**12**) were elucidated by FT-IR, ^1^H NMR, ^13^C NMR (APT), and mass spectrometric analyses. In the FT-IR spectra of compounds **7**–**12**, characteristic symmetric and asymmetric stretching vibrations of the −NH_2_ groups were observed as sharp bands in the range of 3136–3477 cm^–1^. In addition, absorption bands corresponding to C=N and C=S stretching, supporting the presence of the thiocarbamoylpyrazoline moiety, were detected in the ranges of 1576–1627 cm^–1^ and 1332–1399 cm^–1^, respectively, consistent with the proposed structures. LC-Q-TOF-MS analysis showed a protonated ion peak at *m*/*z* 242 ([M + H]^+^), consistent with the calculated value of 242 for chalcones.

Analysis of the ^1^H NMR spectra of compounds **7**–**12** revealed characteristic splitting patterns consistent with the structure of the pyrazoline ring. Specifically, the diastereotopic methylene protons at the C-4 position (C4–H_A_ and C4–H_B_) appeared as doublet of doublets (dd) within the chemical shift range of δ 3.01–3.22 ppm for C4–H_A_ (*J*_AX_ = 4 Hz, *J*_AB_ = 16 Hz or *J*_AB_ = 20 Hz) and δ 3.74–4.02 ppm for C4–H_B_ (*J*_BX_ = 12 Hz, *J*_BA_ = 16 Hz or *J*_BA_ = 20 Hz). Additionally, a doublet of doublets (dd) was observed at δ 5.85–6.04 ppm with the coupling constants *J*_5–4a_ = 4 Hz, *J*_5–4b_ = 12 Hz, corresponding to the methine proton at the C-5 position (C5–H_X_) of the pyrazoline ring. These splitting patterns are indicative of an ABX spin system, arising from geminal and vicinal couplings between the nonequivalent methylene protons (H_A_ and H_B_ at C-4) and the adjacent methine proton (H_X_ at C-5).

This coupling behavior is a hallmark feature of diastereotopic methylene and methine environments, providing further evidence for the integrity of the pyrazoline core structure (Figure 2).^56^

**Figure 2:**
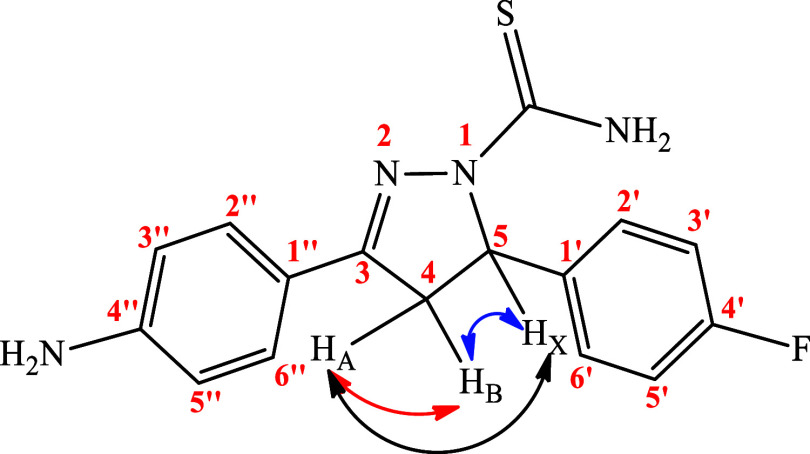
ABX spin system of pyrazoline ring.

In addition, thione/thiol tautomerism in the carbothioamide group was observed in the NMR spectra of compounds **7**–**12** (Figure 3). Spectral results indicate that the compounds are in the thiol form in ^1^H NMR, with broad singlets for the −SH and −NH peaks resonating in the ranges of 7.86–8.10 ppm and 7.64–8.04 ppm, respectively.^57,58^

**Figure 3:**
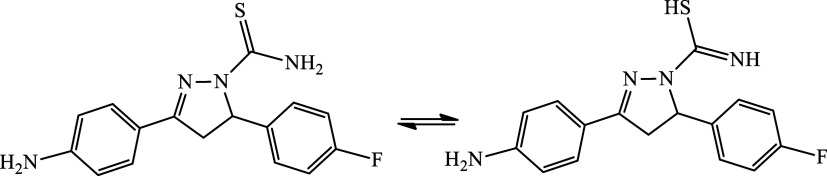
Tautomerism equilibrium in compound **12**.

When examining the ^13^C- NMR spectra (APT) of the compounds, the quaternary peak around 148.48–156.35 ppm belongs to the C-3 carbon formed as a result of cyclization, and the −CH_2_– (C-4) peaks around 41.80–44.05 ppm and −CH– (C-5) peaks around 56.32–62.39 ppm are the most obvious indications of the pyrazoline ring and support the structure. In addition, the peaks resonated in the range of 175.35–175.57 ppm, belonging to the quaternary −C=S carbon, confirm the presence of the carbothioamide group in the structure. The disappearance of the carbonyl carbon peak, which resonates in the 187–191 ppm range of the starting compounds, chalcones, is another piece of spectral evidence that supports the structure. In LC-Q-TOF-MS analyses, the protonated molecular ion peaks at *m*/*z* 315 ([M + H]^+^) are in good agreement with the calculated values for the synthesized thiocarbamoylpyrazoline compounds.

In this study, the ^1^H NMR spectra show signals consistent with the thiol form, whereas the ^13^C NMR data support the predominance of the thione form. This difference arises from the dynamic thione-thiol tautomerism and the differing acquisition times: ^1^H NMR is recorded rapidly, while ^13^C NMR requires longer acquisition, allowing the thermodynamically more stable thioamide to dominate. The broad ^1^H NMR signals (δ 7.64–8.04 ppm for −NH and 7.86–8.10 ppm for −SH) reflect rapidly exchanging protons and hydrogen-bonding effects. In contrast, the ^13^C NMR signal in the range of δ 175.35–175.57 ppm, characteristic of a C=S carbon, confirms the thione form. No C=N resonance was observed (∼160–170 ppm), supporting the depiction of the thioamide as the dominant tautomer.^57,58^ Furthermore, the intense C=S vibrations observed in the FT-IR spectra in the 1332–1339 cm^–1^ frequency range also confirm that the compounds are in the thione form even in the solid state.

All spectroscopic results support the proposed structures. In addition to the novelty of compounds **7–11**, no studies have been found in the literature regarding the activities of any of the synthesized compounds (**1–12**) against MG63 and SW1353 bone cancers. In this study, these compounds were synthesized as potential derivatives of bioactive molecules.

All spectra for the synthesized compounds are provided in the Supporting Information.

### 2.2 Biological Evaluation

#### 2.2.1 Evaluation of Items According to NCI60 Screening Methodology

MTT results reveal that the tested compounds exhibit strong antiproliferative activity in the low-micromolar range against the MG63 and SW1353 cell lines (Table 1). The GI50 values, mostly ranging from 4.42 to 7.67 μM (MG63) and from 3.63 to 5.97 μM (SW1353), indicate that the compounds have a significant cytostatic effect (Table 1). In this context, compound **2** stands out as the strongest antiproliferative agent with GI_50_: 4.81 and 5.05 μM, and IC_50_: 9.98 and 11.44 μM in MG63 and SW1353 cells, respectively, while compound 1 similarly showed high efficacy with GI_50_: 5.47 and 5.01 μM, and IC_50_: 15.25 and 18.32 μM (Table 1). In contrast, compound **4** stands out as the most selective compound, despite having a higher GI_50_ (7.67 μM) in MG63, reaching a significantly higher TGI (845.1 μM) in normal HC cells, and exhibiting the highest selectivity index (TSI: 14.35) (Table 1). When TGI values are examined, it is seen that the concentrations required to completely halt growth in SW1353 cells (e.g., 9.32 μM for compound **2**, 16.41 μM for compound **4**) are lower than in MG63 (Table 1). The LC_50_ values being >1590–2072 μM for most compounds indicate that proliferation is suppressed rather than cell death. The fact that TGI values in normal HC cells generally range from 55.78 to 845.1 μM confirms that the compounds exhibit significant selectivity against tumor cells (Table 1). In contrast, the reference agent, 5-fluorouracil, exhibited low selectivity, with GI_50_: 10.99–12.07 μM, TGI: 393.9–451.7 μM, and TSI: 1.09. Overall, compounds **2**, **1**, and **4** stand out as the most promising candidates in terms of both efficacy and selectivity (Table 1).

**Table 1:** GI_50_, TGI, and LC_50_ Values of the Substances in MG63, SW1353, and HC Cell Lines^a^ ^,^ ^b^ ^,^ ^c^

	**MG63**				**SW1353**				**HC**				
**compound** (μM)	**GI**_**50**_	**TGI**	**IC50**	**LC**_**50**_	**GI**_**50**_	**TGI**	**IC**_**50**_	**LC**_**50**_	**GI**_**50**_	**TGI**	**IC**_**50**_	**LC**_**50**_	**TSI**
**1**	5.47	27.14 ± 1.1	15.25 ± 0.7	>2072	5.01	12.68 ± 0.6	18.32 ± 0.9	>2072	4.60	90.82 ± 3.2	97.63 ± 1.9	>2072	**4.56**
**2**	4.81	37.47 ± 1.4	9.98	425.5 ± 8.1	5.05	9.32	11.44 ± 0.6	118.0 ± 3.4	5.72	113.45 ± 4.4	121.52 ± 2.0	>2072	**4.85**
**3**	4.93	46.66 ± 1.9	33.07 ± 1.1	612.3 ± 9.5	5.84	13.97 ± 0.6	18.32 ± 0.7	294.0 ± 4.7	8.74	55.78 ± 1.9	76.83 ± 1.7	>2072	**1.84**
**4**	7.67	101.38 ± 3.5	136.5 ± 2.8	857.2 ± 12.6	5.97	16.41 ± 0.7	22.66 ± 0.9	714.5 ± 6.0	13.88 ± 0.6	845.1 ± 11.4	685.6 ± 9.5	>2072	**14.35**
**5**	6.75	58.89 ± 2.7	53.56 ± 1.5	781.3 ± 11.7	5.88	13.22 ± 0.6	16.95 ± 0.8	198.8 ± 3.1	8.66	57.06 ± 1.5	77.21 ± 1.6	>2072	**1.58**
**6**	6.09	62.16 ± 2.4	92.88 ± 1.9	1346.5 ± 15.3	6.01	15.33 ± 0.7	20.56 ± 0.8	416.5 ± 3.9	9.32	93.74 ± 1.8	137.14 ± 1.9	>2072	**2.42**
**7**	4.96	162.0 ± 7.5	165.7 ± 2.8	>1590	3.72	170.3 ± 6.3	183.3 ± 3.6	>1590	4.80	220.6 ± 3.6	163.1 ± 2.2	>1590	**1.33**
**8**	5.47	182.1 ± 7.6	195.8 ± 3.0	>1590	4.77	109.2 ± 4.1	179.7 ± 3.0	>1590	5.38	237.2 ± 3.7	192.2 ± 2.7	>1590	**1.63**
**9**	5.82	207.2 ± 8.1	175.0 ± 2.7	>1590	5.66	90.1 ± 3.9	111.0 ± 2.5	>1590	5.57	300.0 ± 3.9	270.6 ± 2.9	>1590	**2.02**
**10**	5.31	190.0 ± 8.0	236.9 ± 3.2	>1590	4.83	171.2 ± 4.2	194.7 ± 2.9	>1590	5.12	715.2 ± 8.0	629.4 ± 5.3	>1590	**3.96**
**11**	5.18	196.7 ± 7.9	169.0 ± 2.8	>1590	3.63	245.8 ± 5.6	254.7 ± 3.2	>1590	5.09	258.7 ± 4.1	237.5 ± 3.0	>1590	**1.17**
**12**	4.42	209.9 ± 7.8	227.7 ± 2.9	>1590	4.29	221.6 ± 5.8	230.3 ± 3.1	>1590	5.41	257.7 ± 4.4	266.6 ± 2.9	>1590	**1.19**
**5-FU**	10.1 ± 0.6	451.7 ± 9.6	527.1 ± 5.6	>3843	12.1 ± 0.6	393.9 ± 6.7	471.0 ± 4.6	>3843	11.6 ± 0.6	462.1 ± 4.3	440.7 ± 4.5	>3843	**1.09**

a Percent inhibition indicated is ±SDs of three independent measurements.

b If the percentage inhibition is less than 10, the SD value is <0.5.

c GI_50_, concentration causing 50% growth inhibition; TGI, total growth inhibition concentration; LC_50_, lethal concentration causing 50% cell death; IC_50_, concentration causing 50% inhibition of cell viability; and TSI, tumor selectivity index.

When the cytotoxic activity of the test molecules on membranes was assessed in Table 2, it was revealed that substances **4** (% 5.9), **7** (% 6.7), **9** (% 7.6), **10** (% 5.9), **11** (% 7.8), and **12** (% 6.4) were less damaging to the normal HC cell membrane. Furthermore, it was evaluated through the cell membrane damage test (LDH test) that substances **1** (% 14.1), **6** (% 13.8), and **8** (% 12.8) were as toxic as the control drug 5-FU (% 11), while the other substances exhibited toxicity in the range of 15–20% [Table 2]. When MTT and LDH tests are evaluated together, it can be concluded that substances **4** and **10** cause the optimal anticancer effect while causing the least damage to normal cells. Accordingly, these results indicate that the chalcone derivative bearing an amino group in the para position and a fluorine atom in the ortho position, along with its corresponding thiocarbamoylpyrazoline derivative, may be the most promising candidates for anticancer activity among the synthesized compounds.

**Table 2:** % Cytotoxicity Values of the Substances at TGI Concentration^a^ ^,^ ^b^

	cancer		control
% cytotoxicity	**MG63**	**SW1353**	**HC**
**1**	24.7 ± 1.3	30.7 ± 1.8	14.1 ± 1.0
**2**	24.0 ± 1.3	43.1 ± 2.1	18.8 ± 1.0
**3**	14.0 ± 1.1	32.5 ± 1.9	19.6 ± 1.2
**4**	20.8 ± 1.2	31.2 ± 1.6	**5.9**
**5**	15.8 ± 1.0	29.6 ± 1.4	19.3 ± 1.1
**6**	12.3 ± 0.9	33.5 ± 1.3	13.8 ± 0.9
**7**	11.7 ± 0.9	6.4	6.7
**8**	7.6	16.2 ± 1.0	12.8 ± 0.9
**9**	12.2 ± 1.0	17.1 ± 1.0	7.6
**10**	6.1	7.7	**5.9**
**11**	9.4	10.0 ± 0.8	7.8
**12**	11.4 ± 1.0	11.1 ± 0.9	6.4
**5-FU**	12.7 ± 0.9	13.8 ± 1.0	11.0 ± 1.0

a Percent cytotoxicity indicated is ± SDs of three independent measurements.

b If the percentage cytotoxicity is less than 10, the SD value is <0.5.

### 2.3 In Silico Studies

Data from computer-aided drug design studies were compared to data from in vitro studies using molecular docking. Thus, *in silico* analysis of compounds identified as active was also obtained. Tables 1 and 2, presenting in vitro data, were used as a basis for molecular docking analysis. The crystal structure selection of targets in molecular docking was also guided by the *in vitro* results. Based on these data, the targets listed in Table 3, namely, human (MP-13) metalloproteinase-13 (3KEJ),^59^ caspase-3 (1GFW),^60^ human estrogen receptor (3ERT),^61^ and (EGFR) epidermal growth factor receptor (1M17),^62^ were initially identified to support cancer cell line studies.

**Table 3:** Binding Parameter Values of Compounds **1, 2, 4, 10** Interact *In Silico* with the Crystal Structures of the Identified Targets

**target**	**crystal structure**	**compound**	**docking score**	**glide emodel**	**glide energy**
**human metalloproteinase-13**	3KEJ	**1**	–6.315	–50.019	–37.274
		**2**	–7.913	–55.286	–39.064
		**4**	–7.459	–56.639	–41.300
		**10**	–7.489	–57.437	–44.957
**caspase-3**	1GFW	**1**	–5.210	–30.875	–25.827
		**2**	–5.467	–35.027	–26.947
		**4**	–6.081	–34.223	–26.246
		**10**	–5.249	–39.846	–31.817
**human estrogen receptor**	3ERT	**1**	–8.257	–46.271	–33.577
		**2**	–6.271	–41.922	–30.391
		**4**	–7.436	–45.796	–34.177
		**10**	–8.570	–67.660	–45.148
**EGFR**	1M17	**1**	–7.067	–49.416	–34.992
		**2**	–6.730	–48.699	–35.335
		**4**	–6.732	–46.811	–33.468
		**10**	–7.837	–59.583	–43.379

When MP-13 was considered to play a critical role in bone cancer, the crystal structure of this target was determined from the Protein Data Bank, and compounds (**1**, **2**, **4**, and **10**) that were active *in vitro* were subjected to molecular docking analysis. These data, obtained according to molecular docking calculations, are shown in Table 3. When the data calculated according to molecular docking for the MP-13 target were analyzed in Table 3, the docking scores for compounds **1**, **2**, **4**, and **10** are −6.315, −7.913, −7.459, and −7.489 kcal/mol, respectively.

The binding modes of the *in silico* molecular docking interaction of compounds **1**, **2**, **4**, and **10** with the crystal structure of the MP-13 target and the types of bonds that the compounds make with amino acid residues are presented in Figure 4. Each 2D interaction diagram in Figure 4, which presents the interaction with the crystal structure of the MP-13 target, was analyzed. It was determined that compound **1** had a polar interaction with the 3KEJ crystal structure at the site of the −F substituent, a hydrogen bond interaction with the amino acid Met253 via the −NH_2_ group, a π–π stacking interaction with the amino acid Tyr246 via the phenyl ring, and a hydrophobic interaction (Figure 4A). When the visual of the binding mode of the same target with compound **2** was examined in Figure 4B, a hydrophobic interaction was determined by the −F side, a hydrogen bond interaction with the amino acid residue Met263 via the −NH_2_ group, and a charged (positive) interaction with the phenyl ring. In Figure 4C, because of the interaction of compound **4** with the crystal structure of MP-13, it was determined that there was a hydrogen-bonding interaction via the amino acid −NH_2_, a π–π stacking interaction with the amino acid Tyr246 via the phenyl ring, and a hydrophobic interaction with −F. Finally, *in silico* interaction of the same target with compound **10**, a hydrogen-bonding interaction with the amino acid residue Lys249 via = S, a hydrogen-bonding interaction with the amino acid Phe241 via −NH_2_, π–π stacking interactions on the phenyl ring, and a polar interaction on the side of −F occurred (Figure 4D).

**Figure 4:**
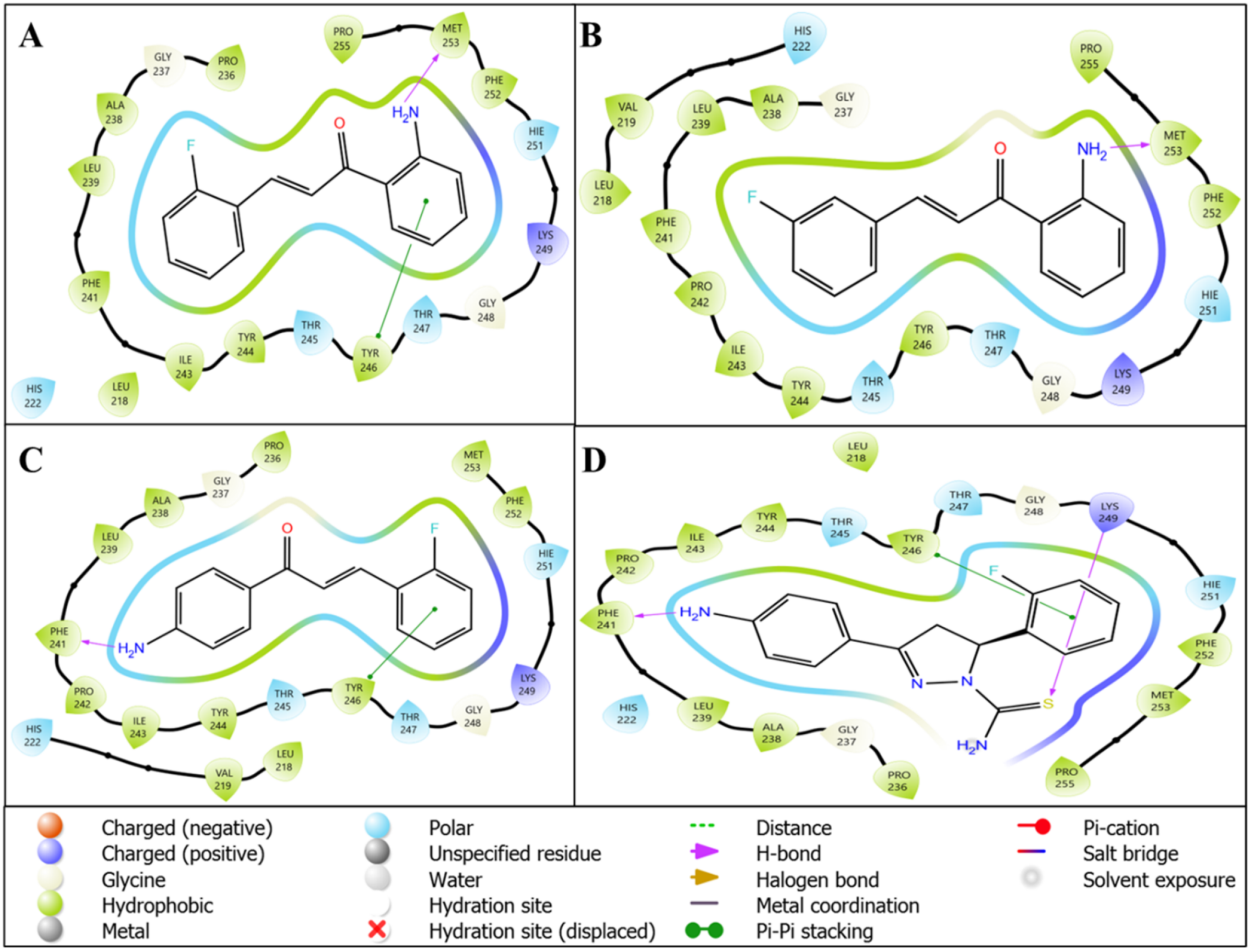
2D diagrams of the interactions of (A) compound **1**, (B) compound **2**, (C) compound **4**, and (D) compound **10** with the crystal structure PDB ID: 3KEJ.

Caspase-3, which plays a crucial role in the apoptosis of cancer cells, was identified as the second target in this study. Compounds **1**, **2**, **4**, and **10** were compared in silico with the crystal structure (1GFW) obtained from the Protein Data Bank. These binding parameter values are presented in Table 3. When the binding parameter values of compounds **1**, **2**, **4**, and **10** for this target were examined, the docking score, Glide model, and Glide energy values of compound **4** were −6.081, −34.223, and −26.246 kcal/mol, respectively. Additionally, 2D diagrams of these binding parameter values are presented in Figure 5.

**Figure 5:**
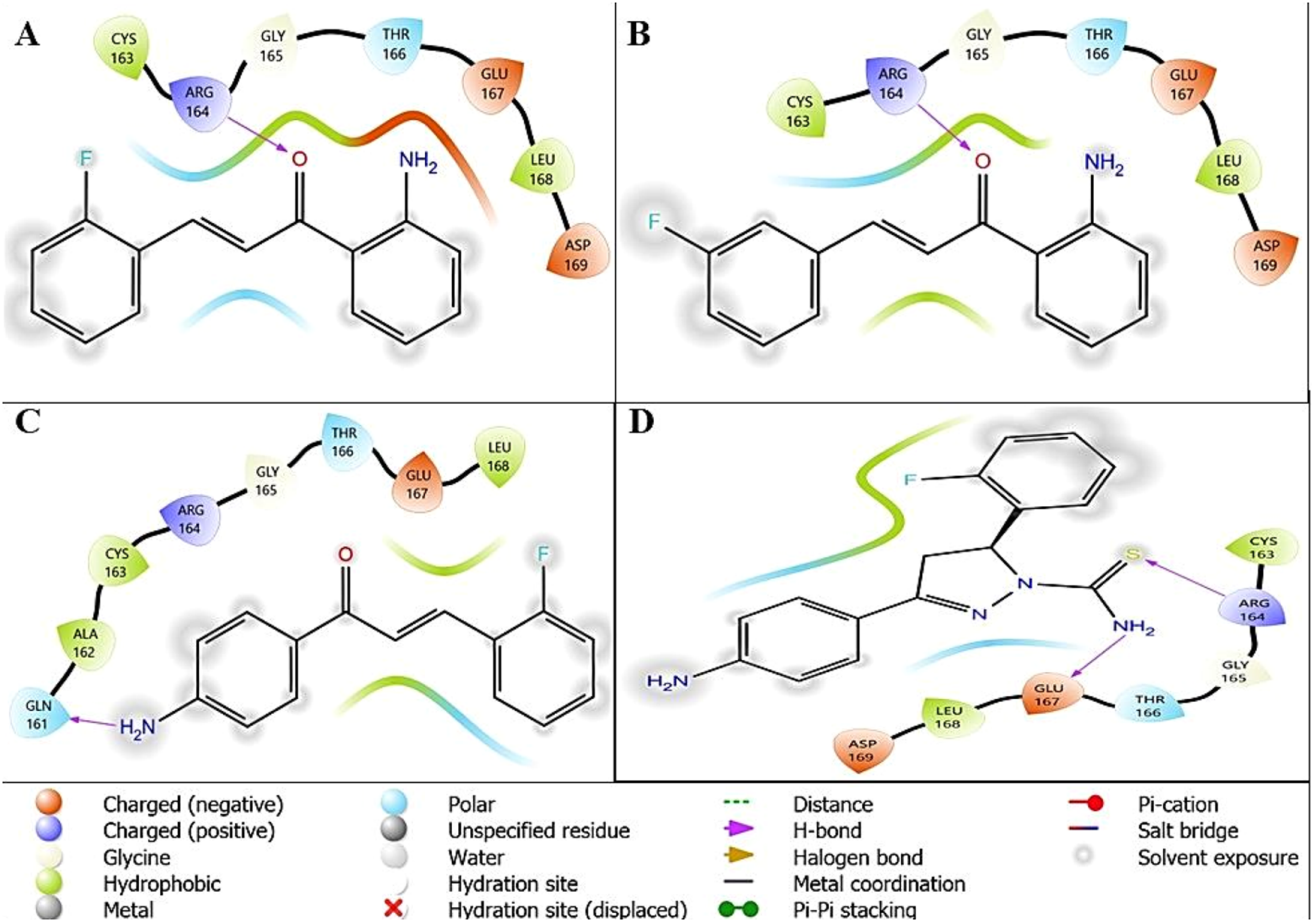
2D diagrams of the interactions of (A) compound **1**, (B) compound **2**, (C) compound **4**, and (D) compound **10** with the crystal structure PDB ID: 1GFW.

Another target identified using in silico approaches is the human estrogen receptor. To support the data from the in vitro study, the crystal structure of this target was determined as 3ERT from the Protein Data Bank. This crystal structure was interacted with compounds **1**, **2**, **4**, and **10** using the molecular docking method, and calculations were performed. The obtained data are presented in Table 3. The docking scores of the crystal structure of the human estrogen receptor (3ERT) were calculated as −8.257 kcal/mol for compound **1**, −6.271 kcal/mol for compound **2**, −7.436 kcal/mol for compound **4**, and −8.570 kcal/mol for compound **10**, respectively. In addition, the 2D graph of the interaction of the human estrogen receptor with the four related compounds is shown in Figure 6. Figure 6A shows that compound **1** is docked in the active pocket region of the 3ERT crystal structure, interacts hydrophobically, and forms a hydrogen bond with amino acid residue Leu387 via −NH_2_. The hydrophobic interaction of compound **2** via the −NH_2_ group is shown in Figure 6B. Figure 6C shows that compound **4**, while docked in the active pocket region of the human estrogen receptor target, exhibits a hydrophobic and polar interactions via the −F atom and hydrogen bonds with the Asp351 amino acid via the −NH_2_ group. Figure 6D shows that compound **10**, which is docked in the active binding region of the 3ERT crystal structure, hydrogen bonds with the Asp351 amino acid via the −NH_2_ group.

**Figure 6:**
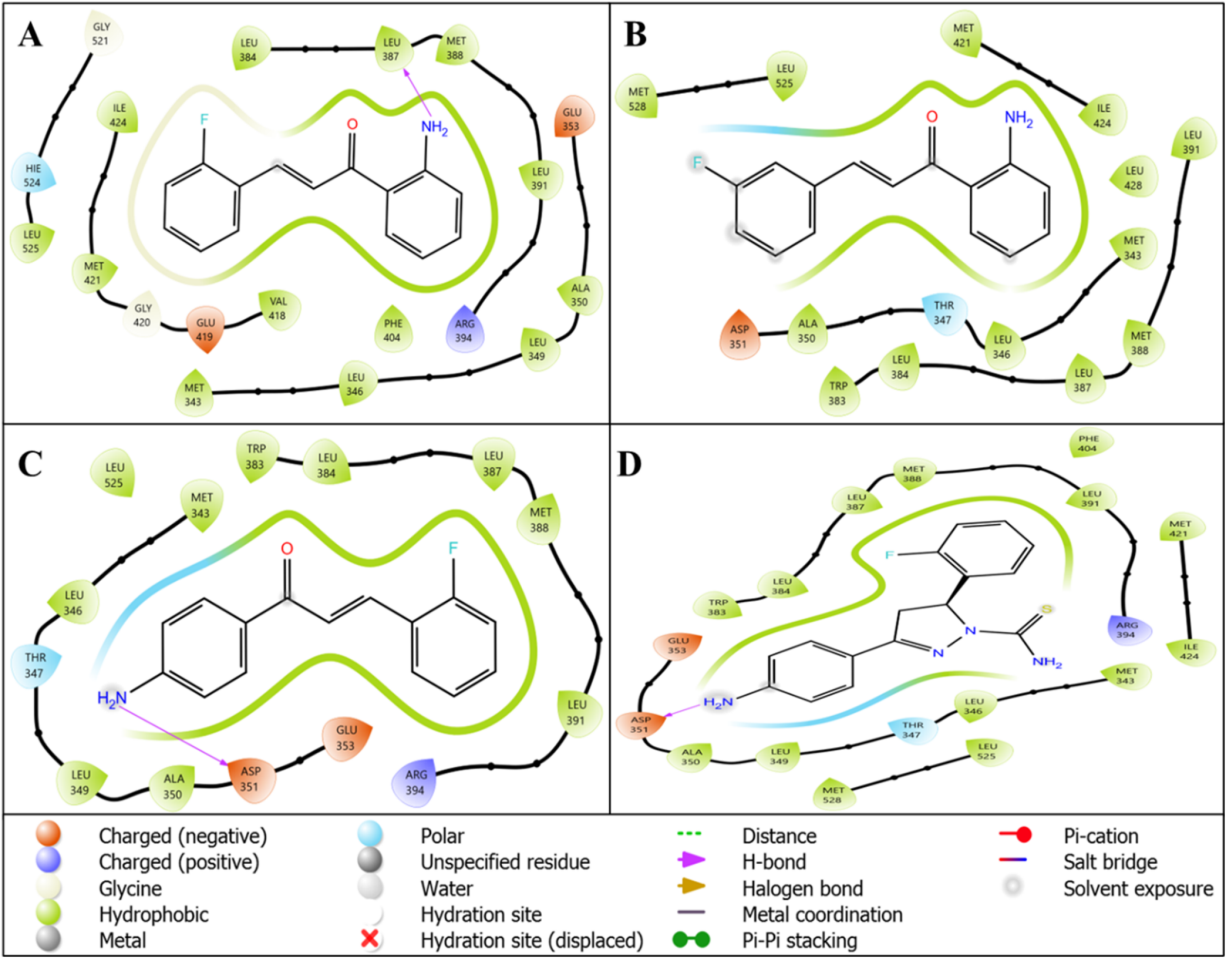
2D diagrams of the interactions of (A) compound **1**, (B) compound **2**, (C) compound **4**, and (D) compound **10** with the crystal structure PDB ID: 3ERT.

In a molecular docking study, compounds **1**, **2**, **4**, and **10** interacted in silico with the EGFR target, which inhibits the progression and growth of cancer cells. According to the numerical values of this interaction presented in Table 3, the docking score for compound **10** was −7.837 kcal/mol, the Glide model value was −59.583 kcal/mol, and the Glide energy was −43.379 kcal/mol. In addition, the docking scores for EGFR were calculated as −7.067 for compound **1**, −6.730 for compound **2**, and −4.732 for compound **4**, respectively. In addition to these parameter values, other data obtained were the binding modes. Figure 7 presents the 2D interaction diagrams of compounds **1**, **2**, **4**, and **10** that interacted with EGFR in silico. The hydrogen bond interaction of compound 1 with the amino acid residue Glu738 via the −NH_2_ group, the hydrogen bond interaction with the amino acid residue Lys721 via = O, and the hydrophobic interaction by the −F atom were determined in Figure 7A. Figure 7B shows the hydrogen bond interaction of compound **2**, which is located in the active pocket region of the crystal structure of the relevant target, with the amino acid residue Glu738 via the NH_2_ group, and the hydrogen bond interaction with the amino acid residue Lys721 via = O. Figure 7C shows the hydrogen bond interaction with the amino acid residue Leu764 via −NH_2_, the hydrogen bond interaction with the amino acid residue Thr830 via = O, and the hydrophobic and partially polar interaction at the region where Met769 is docked. It was determined that compound **10**, which has the best binding parameter value for the EGFR target, is docked in the active pocket region in the 2-dimensional interaction diagram in Figure 7D, has a hydrogen bond interaction with the Asp831 amino acid residue via −NH_2_, a hydrogen bond with Met769, an important acid in EGFR, via −NH_2_, and also a hydrophobic interaction.

**Figure 7:**
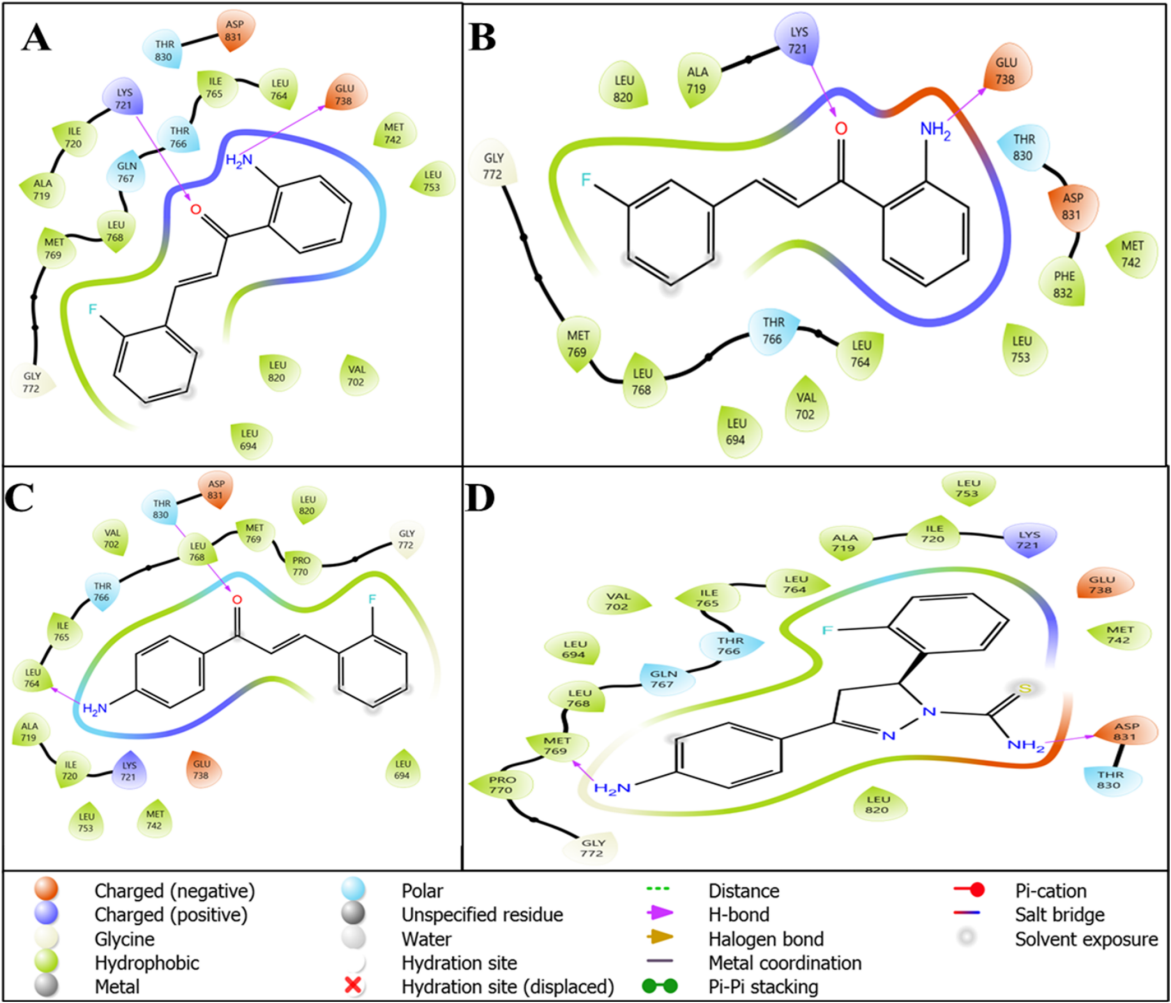
2D diagrams of the interactions of (A) compound **1**, (B) compound **2**, (C) compound **4**, and (D) compound **10** with the crystal structure PDB ID: 1M17.

## 3 Conclusion

In this study, a series of bioactive fluoro-substituted aminochalcones (**1–6**) and their corresponding thiocarbamoylpyrazoline derivatives (**7–12**) were synthesized. The anticancer effects of these compounds on MG63 and SW1353 bone cancer cell lines were evaluated for the first time. Among the tested compounds, chalcone **4** and thiocarpyrazoline compound (**10**) synthesized from it exhibited the most promising anticancer activity, selectively targeting cancer cells while sparing healthy ones. In this study, in addition to synthesis, design, and in vitro methods, in silico approaches were also applied. *In silico* studies were conducted using molecular docking. The molecular docking study analyzed cancer targets (MP-13, caspase-3, human estrogen receptor, EGFR). Compounds **1**, **2**, **4**, and **10** could theoretically be potential drug candidates for anticancer studies.

## 4 Experimental Section

The materials and equipment used in this study are detailed in the Supporting Information.

### 4.1 Methods

#### 4.1.1 General Procedure for the Synthesis of Chalcones (1–6)

Chalcone derivatives were synthesized via the Claisen–Schmidt condensation reaction, following previously reported procedures.^9–12^ Sodium hydroxide (5 g, 12.5 mmol) was dissolved in a 30 mL mixture of water and ethanol (1:2 v/v) under magnetic stirring at room temperature. Separately, 2′- or 4′-aminoacetophenone (1.35 g, 10 mmol) was dissolved in ethanol (10 mL) and added to the alkaline solution with constant stirring until fully dissolved.

2-, 3-, or 4-Fluorobenzaldehyde (1.08, 1.09, or 1.09 mL; 10 mmol, respectively), each dissolved in ethanol (10 mL), was added dropwise to the reaction mixture over a period of 30 min. The resulting mixtures were stirred at room temperature for 12 h. The reaction progress was monitored by thin-layer chromatography (TLC). Upon completion, water was added to the reaction mixture to induce precipitation. The mixtures were refrigerated overnight to allow the solid products to mature. The resulting yellow precipitates were filtered by using a sintered glass crucible and washed with cold water. The crude products were dried by using a lyophilizer. The structures of the synthesized chalcones were elucidated using spectroscopic and spectrometric techniques, including ^1^H NMR, ^13^C NMR (APT), HRMS, FT-IR spectroscopy, and elemental analysis.^9–13^ The general synthetic route is listed in Scheme 2.

**Scheme 2:**
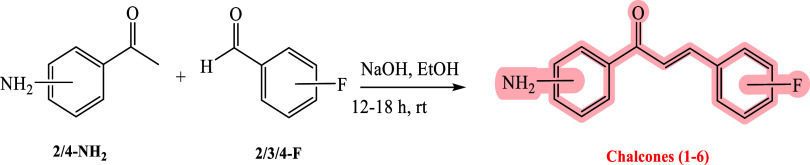
General Synthetic Route of Chalcones (**1–6**)

The numbering of the atoms in compounds **1** and **7** is given in Figure 8 as an example for spectroscopic analysis of chalcones and thiocarbamoylpyrazolines.

**Figure 8:**
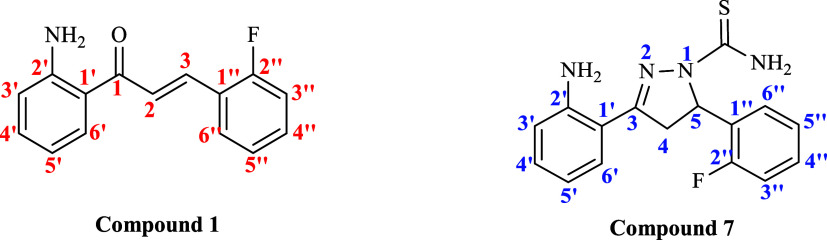
Numbering of the atoms in compounds **1** and **7**.

##### 4.1.1.1 (2*E*)-1-(2-Aminophenyl)-3-(2-fluorophenyl)­prop-2-en-1-one (1)

Yield: 77%. Yellow solid, M.p.: 88–90 °C (lit.:91–93 °C),^51^ Rf: 0.70 (hexane-diethyl ether 1:3). FT-IR (cm^–1^): 3440, 3323 (−NH_2_), 1637 (C=O), 1569 (C=C). ^1^H NMR (400 MHz, CDCl_3_, ppm): δ, 7.89 (d, *J* = 8.0 Hz, 1H, H-6′); 7.86 (AB, *J* = 16.0 Hz, 1H, H-3); 7.76 (AB, *J* = 16.0 Hz, 1H, H-2); δ= 7.67 (app t, *J* = 8.0 Hz, 1H, H-4′); 7.41 (d, *J* = 8.0 Hz, 1H, H-6″); 7.35 (app t, *J* = 8.0 Hz, 1H, H-4″); 7.22 (app t, *J* = 8.0 Hz, 1H, H-5″); 7.16 (app t, *J* = 8.0 Hz, 1H, H-3″); 6.75 (d, *J* = 8.0 Hz, 1H, H-3′); δ = 6.72 (app t, *J* = 8.0 Hz, 1H, H-5′); 6.39 (bs, −NH_2_). ^13^C NMR (100 MHz, CDCl_3_, ppm): 191.63 (C-1), 162.89, 160.37 (d, *J*_C–F_ = 252 Hz, C-2″), 151.10 (C-2′), 135.64, 135.62 (d, *J*_C–F_ = 2 Hz, C-3), 134.45 (C-4′), 131.41, 131.33 (d, *J*_C–F_ = 8 Hz, C-4″), 131.11 (C-6′), 129.63, 129.60 (d, *J*_C–F_ = 3 Hz, C-6″), 125.81, 125.74 (d, *J*_C–F_ = 7 Hz, C-5″), 124.46, 124.42 (d, *J*_C–F_ = 4 Hz, C-2), 123.45, 123.34 (d, *J*_C–F_ = 11 Hz, C-1″), 118.93 (C-1′), 117.32 (C-5′), 116.37, 116.15 (d, *J*_C–F_ = 22 Hz, C-3″), 115.93 (C-3′). LC-Q-TOF-MS *m*/*z* calcd for C_15_H_12_FNO [M + H]^+^: 242.1008, Found: 242.1005. Elemental analysis (%) calcd for C_15_H_12_FNO: C 74.68, H 5.01, N 5.81; found: C 74.63, H 5.02, N 5.81.

##### 4.1.1.2 (2*E*)-1-(2-Aminophenyl)-3-(3-fluorophenyl)­prop-2-en-1-one (2)

Yield: 85%. Yellow solid, M.p.: 102–104 °C (lit.:102–104 °C).^51^ Rf: 0.60 (hexane-diethyl ether 1:3). FT-IR (cm^–1^): 3439, 3321 (−NH_2_), 1639 (C=O), 1574 (C=C). ^1^H NMR (400 MHz, CDCl_3_, ppm): δ, 7.87 (d, *J* = 8.0 Hz, 1H, H-6′); 7.71 (AB, *J* = 16.0 Hz, 1H, H-3); 7.63 (AB, *J* = 16.0 Hz, 1H, H-2); 7.42–7.31 (m, 4H, H-4′/2″/5″/6″); 7.15–7.09 (m, 1H, H-4″); 6.75–6.71 (m, 2H, H-3′/5′); 6.40 (bs, 2H, −NH_2_).^13^C NMR (100 MHz, CDCl_3_, ppm): 191.29 (C-1), 164.29, 161.84 (d, *J*_C–F_ = 245 Hz, C-3″), 151.14 (C-2′), 141.45, 141.42 (d, *J*_C–F_ = 3 Hz, C-3), 137.60, 137.53 (d, *J*_C–F_ = 7 Hz, C-1″), 134.52 (C-4′), 131.04 (C-6′), 130.49, 130.41 (d, *J*_C–F_ = 8 Hz, C-5″), 124.37, 124.34 (d, *J*_C–F_ = 3 Hz, C-6″); 124.31 (C-2), 118.79 (C-1′), 117.37 (C-5′), 117.04, 116.83 (d, *J*_C–F_ = 21 Hz, C-4″), 115.94 (C-3′), 114.45, 114.23 (d, *J*_C–F_ = 22 Hz, C-2″). LC-Q-TOF-MS *m*/*z* calcd for C_15_H_12_FNO [M + H]^+^: 242.0981, Found: 242.1005. Elemental analysis (%) calcd for C_15_H_12_FNO: C 74.68, H 5.01, N 5.81; found: C 74.65, H 5.02, N 5.81.

##### 4.1.1.3 (2*E*)-1-(2-Aminophenyl)-3-(4-fluorophenyl)­prop-2-en-1-one (3)

Yield: 88%. Yellow solid, M.p.: 85–87 °C (lit.:87–89 °C).^51^ Rf: 0.60 (hexane-diethyl ether 1:3). FT-IR (cm^–1^): 3427, 3316 (−NH_2_), 1647 (C=O), 1575 (C=C). ^1^H NMR (400 MHz, CDCl_3_, ppm): δ, 7.88 (d, *J* = 8.0 Hz, 1H, H-6′); 7.73 (AB, *J* = 16.0 Hz, 1H, H-3); 7.67–7.62 (dd, *J*_1_ = 8.0 Hz, *J*_2_ = 4.0 Hz, 2H, H-2″/6″); 7.57 (AB, *J* = 16.0 Hz, 1H, H-2); 7.32 (app t, *J* = 8.0 Hz, 1H, H-4′); 7.13 (d, *J* = 8.0 Hz, 2H, H-3″/5″); 6.74–6.71 (m, 2H, H-3′/H-5′); 6.37 (bs, −NH_2_). ^13^C NMR (100 MHz, CDCl_3_, ppm): 191.48 (C-1), 165.06, 162.57 (d, *J*_C–F_ = 249 Hz, C-4″), 151.04 (C-2′), 141.66 (C-3), 134.40 (C-4′), 131.53, 131.50 (d, *J*_C–F_ = 3.0 Hz, C-1″), 130.98 (C-6′), 130.16, 130.08 (d, *J*_C–F_ = 8.0 Hz, C-2″/6″), 122.83, 122.80 (d, *J*_C–F_ = 3.0 Hz, C-2), 118.95 (C-1′), 117.36 (C-5′), 116.17 (C-3′), 115.95, 115.91 (d, *J*_C–F_ = 4 Hz, C-3″/5″). LC-Q-TOF-MS *m*/*z* calcd for C_15_H_12_FNO [M + H]^+^: 242.1008, Found: 242.1000. Elemental analysis (%) calcd for C_15_H_12_FNO: C 74.68, H 5.01, N 5.81; found: C 74.68, H 5.01, N 5.81.

##### 4.1.1.4 (2*E*)-1-(4-Aminophenyl)-3-(2-fluorophenyl)­prop-2-en-1-one (4)

Yield: 85%. Light yellow solid, M.p.: 141–143 °C (lit.:138–140 °C).^52^ Rf: 0.70 (diethyl ether). FT-IR (cm^–1^): 3330, 3220 (−NH_2_), 1629 (C=O), 1578 (C=C). ^1^H NMR (400 MHz, CDCl_3_, ppm): δ, 7.96 (d, *J* = 8.0 Hz, 2H, H-2′/6′); 7.89 (AB, *J* = 16.0 Hz, 1H, H-3); 7.68 (AB, *J* = 16.0 Hz, 1H, H-2); 7.65 (d, *J* = 8.0 Hz, 1H, H-6″); 7.39–7.36 (m, 1H, H-3″); 7.20 (app t, *J* = 8.0 Hz, 1H, H-4″); 7.14 (app t, *J* = 8.0 Hz, 1H, H-5″); 6.73 (d, *J* = 8.0 Hz, 2H, H-3′/5′); 4.23 (s, 2H, −NH_2_). ^13^C NMR (100 MHz, CDCl_3_, ppm): 188.08 (C-1), 162.93, 160.40 (d, *J*_C–F_ = 253 Hz, C-2″), 151.25 (C-4′), 135.91, 135.89 (d, *J*_C–F_ = 2 Hz, C-3), 131.41, 131.32 (d, *J*_C–F_ = 9 Hz, C-4″), 131.21 (C-2′/6′), 129.76, 129.73 (d, *J*_C–F_ = 3 Hz, C-6″), 128.36 (C-1′), 124.74, 124.67 (d, *J*_C–F_ = 7 Hz, C-5″), 124.46, 124.42 (d, *J*_C–F_ = 4 Hz, C-2), 123.46, 123.35 (d, *J*_C–F_ = 11 Hz, C-1″), 116.36, 116.14 (d, *J*_C–F_ = 22 Hz, C-3″), 113.96 (C-3′/5′). LC-Q-TOF-MS *m*/*z* calcd for C_15_H_12_FNO [M + H]^+^: 242.1008, Found: 242.0995. Elemental analysis (%) calcd for C_15_H_12_FNO: C 74.68, H 5.01, N 5.81; found: C 74.67, H 5.01, N 5.81.

##### 4.1.1.5 (2*E*)-1-(4-Aminophenyl)-3-(3-fluorophenyl)­prop-2-en-1-one (5)

Yield: 74%. Light yellow solid, M.p.: 138–140 °C. Rf: 0.45 (hexane-diethyl ether 1:3). FT-IR (cm^–1^): 3480, 3344 (−NH_2_), 1634 (C=O), 1579 (C=C). ^1^H NMR (400 MHz, CDCl_3_, ppm): δ, 7.96 (d, *J* = 8.0 Hz, 2H, H-2′/6′); 7.75 (AB, *J* = 16.0 Hz, 1H, H-3); 7.56 (AB, *J* = 16.0 Hz, 1H, H-2); 7.43–7.33 (m, 3H, H-2″/5″/6″); 7.12 (app t, *J* = 8.0 Hz, 1H, H-4″); 6.73 (d, *J* = 8.0 Hz, 2H, H-3′/5′); 4.21 (s, 2H, −NH_2_). ^13^C NMR (100 MHz, CDCl_3_, ppm): 187.68 (C-1), 164.28, 161.83 (d, *J*_C–F_ = 245 Hz, C-3″), 151.25 (C-4′), 141.67, 141.64 (d, *J*_C–F_ = 7 Hz, C-3), 137.67, 137.59 (d, *J*_C–F_ = 8 Hz, C-1″), 131.18 (C-2′/6′), 130.47, 130.38 (d, *J*_C–F_ = 9 Hz, C-5″), 128.32 (C-1′), 124.44, 124.41 (d, *J*_C–F_ = 43 Hz, C-6″), 123.18 (C-2), 117.02, 116.81 (d, *J*_C–F_ = 21 Hz, C-4″), 114.39, 114.17 (d, *J*_C–F_ = 22 Hz, C-2″), 113.97 (C-3′/5′). LC-Q-TOF-MS *m*/*z* calcd for C_15_H_12_FNO [M + H]^+^: 242.1008, Found: 242.0992. Elemental analysis (%) calcd for C_15_H_12_FNO: C 74.68, H 5.01, N 5.81; found: C 74.68, H 5.02, N 5.81.

##### 4.1.1.6 (2*E*)-1-(4-Aminophenyl)-3-(4-fluorophenyl)­prop-2-en-1-one (6)

Yield: 79%. Light yellow solid, M.p.: 140–142 °C (lit: 136–138 °C).^53,54^ Rf: 0.70 (diethyl ether). FT-IR (cm^–1^): 3334, 3214 (−NH_2_), 1628 (C=O), 1578 (C=C). ^1^H NMR (400 MHz, CDCl_3_, ppm): δ, 7.95 (d, *J* = 8.0 Hz, 2H, H-2′/6′); 7.77 (AB, *J* = 16.0 Hz, 1H, H-3); 7.66–7.63 (dd, *J*_1_ = 8.0 Hz, *J*_2_ = 4.0 Hz, 2H, H-2″/6″); 7.50 (AB, *J* = 16.0 Hz, 1H, H-2); 7.12 (app t, *J* = 8.0 Hz, 2H, H-3″/5″); 6.71 (d, *J* = 8.0 Hz, 2H, H-3′/5′); 4.20 (s, 2H, −NH_2_). ^13^C NMR (100 MHz, CDCl_3_, ppm): 187.86 (C-1), 165.05, 162.56 (d, *J*_C–F_ = 251 Hz, C-4″), 151.15 (C-4′), 141.86 (C-3), 131.58, 131.55 (d, *J*_C–F_ = 3 Hz, C-1′), 131.10­(C-2′/6′), 130.17, 130.08 (d, *J*_C–F_ = 9 Hz, C-2″/6″), 128.47 (C-1″), 121.71, 121.69 (d, *J*_C–F_ = 2 Hz, C-2), 116.13, 115.92 (d, *J*_C–F_ = 21 Hz, C-3″/5″), 113.96 (C-3′/5′). LC-Q-TOF-MS *m*/*z* calcd for C_15_H_12_FNO [M + H]^+^: 242.1008, Found: 242.0989. Elemental analysis (%) calcd for C_15_H_12_FNO: C 74.68, H 5.01, N 5.81; found: C 74.67, H 5.01, N 5.81.

#### 4.1.2 General Procedure for the Synthesis of Thiocarbamoylpyrazolines (7–12)

Compounds **7–12** were synthesized via the addition reaction of thiosemicarbazide to chalcone derivatives in a basic medium, as described in the literature.^56–60^ For each reaction, a mixture containing the corresponding chalcone (5 mmol), sodium hydroxide (12.5 mmol), and thiosemicarbazide (6 mmol) in ethanol (30 mL) was refluxed for 12 h. The progress of the reaction was monitored by thin-layer chromatography (TLC). After completion of the reaction, the reaction mixtures were cooled to room temperature and poured into ice-cold water with a small amount of salt to cause precipitation. The resulting solids were filtered and dried using a lyophilizer. The crude products were evaluated by TLC and purified, if necessary, by recrystallization and column chromatography. The structures of the synthesized compounds were confirmed by spectroscopic methods including ^1^H NMR, ^13^C NMR (APT), LC-MS (ESI), and FT-IR.^61,62^ Among the synthesized thiocarbamoylpyrazoline compounds, compound number **12** is available in the literature,^63^ and compounds numbers **7**–**11** have not been found in the literature. The synthesis equation of the compounds is given in Scheme 3.

**Scheme 3:**
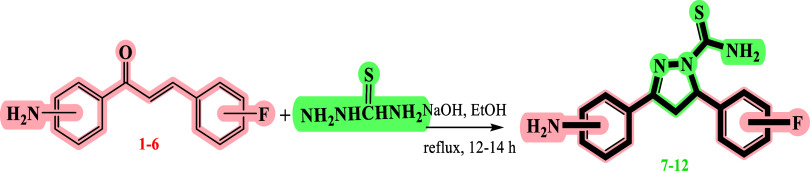
General Synthetic Route of Thiocarbamoylpyrazolines (**7**–**12**)

##### 4.1.2.1 3-(2-Aminophenyl)-5-(2-fluorophenyl)-4,5-dihydro-1*H*-pyrazole-1-carbotioamide (7)

Yield: 63%. White solid, M.p.: 198–200 °C. Rf: 0.60 (diethyl ether). FT-IR (cm^–1^): 3440, 3390 (−NH_2_), 3303 (−NH_2_, H-bonded), 1582 (C=N), 1338 (C=S). ^1^H NMR (400 MHz, DMSO-*d*_6_, ppm): δ, 8.10 (bs, 1H, −SH); 8.04 (bs, 1H, −NH); 7.33–7.10 (m, 5H, H-6′/3″/4″/5″/6″); 6.98 (app t, *J* = 8.0 Hz, 1H, H-4′); 6.82 (d, *J* = 8.0 Hz, 1H, H-3′); 6.68 (bs, 2H, Ar–NH_2_); 6.50 (app t, *J* = 8.0 Hz, 1H, H-5′); 6.04–6.00 (dd, *J*_5/4a_ = 4 Hz, *J*_5/4b_ = 12 Hz, 1H, H-5); 4.02–3.99 (dd, *J*_BA_= 16.0 Hz, *J*_BX_= 12.0 Hz, 1H, H-4b), 3.21–3.15 (dd, *J*_AB_= 20 Hz, *J*_AX_= 4.0 Hz, 1H, H-4a). ^13^C NMR (100 MHz, DMSO-*d*_6_, ppm): 175.45 (C=S), 160.68, 158.25 (d, *J*_C–F_ = 243 Hz, C-2″), 157.71 (C-2′), 148.48 (C-3), 131.55 (C-4′), 130.83 (C-6′), 130.39, 130.25 (d, *J*_C–F_ = 14 Hz, C-1″), 129.41, 129.33 (d, *J*_C–F_ = 8 Hz, C-6″), 127.32, 127.28 (d, *J*_C–F_ = 4 Hz, C-4″), 124.88, 124.84 (d, *J*_C–F_ = 4 Hz, C-5″), 116.12, 115.91 (d, *J*_C–F_ = 21 Hz, C-3″), 115.91 (C-5′), 115.15 (C-3′), 111.37 (C-1′), 56.32 (C-5), 43.04 (C-4). LC-Q-TOF-MS *m*/*z* calcd for C_16_H_15_FN_4_S [M + H]^+^: 315.1080; Found 315.1118. Elemental analysis (%) calcd for C_16_H_15_FN_4_S: C 61.13, H 4.81, N 17.82, S 10.20; found: C 61.13, H 4.81, N 17.82, S 10.22.

##### 4.1.2.2 3-(2-Aminophenyl)-5-(3-fluorophenyl)-4,5-dihydro-1*H*-pyrazole-1-carbotioamide (8)

Yield: 69%. White solid, M.p.: 188–190 °C. Rf: 0.60 (diethyl ether). FT-IR (cm^–1^): 3388, 3290 (−NH_2_), 3164 (−NH_2_, H-bonded), 1581 (C=N), 1332 (C=S). ^1^H NMR (400 MHz, CDCl_3_, ppm): ^1^H NMR (400 MHz, DMSO-*d*_6_, ppm): δ, 8.10 (bs, 1H, −SH); 8.04 (bs, 1H, −NH); 7.39–7.34 (m, 1H, H-6′); 7.19 (d, *J* = 8.0 Hz, 1H, 6″), 7.12 (app t, *J* = 8.0 Hz, 1H, H-5″), 7.07 (app t, *J* = 8.0 Hz, 1H, H-4′); 6.96 (d, *J* = 8.0 Hz, 1H, H-2″); 6.91 (d, *J* = 8.0 Hz, 1H, H-4″); 6.82 (app t, *J* = 8.0 Hz, 1H, H-3′); 6.69 (bs, 2H, Ar–NH_2_); 6.50 (app t, *J* = 8.0 Hz, 1H, H-5′); 5.93–5.89 (dd, *J*_5/4a_ = 4 Hz, *J*_5/4b_ = 12 Hz, 1H, H-5); 3.97–3.90 (dd, *J*_BA_= 16.0 Hz, *J*_BX_= 12.0 Hz, 1H, H-4b), 3.22–3.17 (dd, *J*_AB_= 16 Hz, *J*_AX_= 4.0 Hz, 1H, H-4a). ^13^C NMR (100 MHz, DMSO-*d*_6_, ppm): 175.57 (C=S), 163.88, 161.46 (d, *J*_C–F_ = 242 Hz, C-3″), 157.67 (C-2′), 148.51 (C-3), 146.51 (C-1″), 131.60 (C-4′), 131.13, 131.04 (d, *J*_C–F_ = 9 Hz, C-5″), 130.84 (C-6′), 121.69 (C-6″), 115.95 (C-5′), 115.18 (C-3′), 114.23, 114.02 (d, *J*_C–F_ = 21 Hz, C-2″), 112.78, 112.56 (d, *J*_C–F_ = 20 Hz, C-4″), 111.36 (C-1′), 61.10 (C-5), 43.94 (C-4). LC-Q-TOF-MS *m*/*z* calcd for C_16_H_15_FN_4_S [M + H]^+^: 315.1080; Found 315.1081. Elemental analysis (%) calcd for C_16_H_15_FN_4_S: C 61.13, H 4.81, N 17.82, S 10.20; found: C 61.13, H 4.81, N 17.82, S 10.21.

##### 4.1.2.3 3-(2-Aminophenyl)-5-(4-fluorophenyl)-4,5-dihydro-1*H*-pyrazole-1-carbotioamide (9)

Yield: 71%. White solid, M.p.: 202–204 °C. Rf: 0.62 (diethyl ether). FT-IR (cm^–1^): 3358, 3270 (−NH_2_), 3170 (−NH_2_, H-bonded), 1576 (C=N), 1343 (C=S). ^1^H NMR (400 MHz, CDCl_3_, ppm): δ, 8.05 (bs, 1H, −SH); 7.98 (bs, 1H, −NH); 7.21–7.10 (m, 6H, H-4′/6′/2″/3″/5″/6″); 6.82 (d, *J* = 8.0 Hz, 1H, H-3′); 6.68 (s, 2H, Ar–NH_2_); 6.50 (app t, *J* = 8.0 Hz, 1H, H-5′); 5.89–5.86 (dd, *J*_5/4a_ = 4 Hz, *J*_5/4b_ = 12 Hz, 1H, H-5); 3.96–3.89 (dd, *J*_BA_= 20.0 Hz, *J*_BX_= 8.0 Hz, 1H, H-4b); 3.19–3.14 (dd, *J*_AB_= 20 Hz, *J*_AX_= 4.0 Hz, 1H, H-4a). ^13^C NMR (100 MHz, DMSO-*d*_6_, ppm): 175.48 (C=S), 162.73, 160.32 (d, *J*_C–F_ = 241 Hz, C-4″), 157.67 (C-2′), 148.50 (C-3), 139.83, 139.80 (d, *J*_C–F_ = 3 Hz, C-1″), 131.56 (C-4′), 130.82 (C-6′), 127.89, 127.81 (d, *J*_C–F_ = 8 Hz, C-2″/6″), 115.93 (C-5′), 115.76, 115.55 (d, *J*_C–F_ = 21 Hz, C-3″/5″), 115.16 (C-3′), 111.42­(C-1′), 60.87 (C-5), 44.05 (C-4). LC-Q-TOF-MS *m*/*z* calcd for C_16_H_15_FN_4_S [M + H]^+^: 315.1080; Found 315.1071. Elemental analysis (%) calcd for C_16_H_15_FN_4_S: C 61.13, H 4.81, N 17.82, S 10.20; found: C 61.12, H 4.81, N 17.81, S 10.20.

##### 4.1.2.4 3-(4-Aminophenyl)-5-(2-fluorophenyl)-4,5-dihydro-1*H*-pyrazole-1-carbotioamide (10)

Yield: 75%. White solid, M.p.: 270–272 °C. Rf: 0.68 (diethyl ether). FT-IR (cm^–1^): 3414, 3342 (−NH_2_), 3249 (−NH_2_, H-bonded), 1584 (C=N), 1368 (C=S). ^1^H NMR (400 MHz, DMSO-*d*_6_, ppm): δ, 7.92 (bs, −SH), 7.69 (bs, −NH), 7.54 (d, *J* = 8.0 Hz, 2H, H-2′/6′); 7.31–7.26 (dd, *J*_1_ = 8.0 Hz, *J*_2_ = 4.0 Hz, 1H, H-3″); 7.19 (dd, *J*_1_ = 12.0 Hz, *J*_2_ = 8.0 Hz, 1H, H-6″); 7.12 (app t, *J* = 8.0 Hz, 1H, H-4″); 6.97 (app t, *J* = 8.0 Hz, 1H, H-5″); 6.56 (d, *J* = 8.0 Hz, 2H, H-3′/5′); 6.01–5.98 (dd, *J*_5/4a_ = 4 Hz, *J*_5/4b_ = 12 Hz, 1H, H-5); 5.75 (s, Ar–NH_2_), 3.87–3.80 (dd, *J*_BA_= 16 Hz, *J*_BX_= 12.0 Hz, 1H, H-4b); 3.07–3.01 (dd, *J*_AB_= 20 Hz, *J*_AX_= 4.0 Hz, 1H, H-4a). ^13^C NMR (100 MHz, DMSO-*d*_6_, ppm): 175.35 (C=S), 160.61, 158.18 (d, *J =* 243 Hz, C-2″), 156.35 (C-3), 151.94 (C-4′), 130.31, 130.17 (d, *J*_C–F_ = 14 Hz, C-1″),129.38, 129.30 (d, *J*_C–F_ = 8 Hz, C-6″), 129.23 (C-2′/6′), 127.39, 127.35 (d, *J*_C–F_ = 4 Hz, C-4″), 124.86, 124.83 (d, *J*_C–F_ = 3 Hz, C-5″), 117.90 (C-1′), 116.08, 115.87 (d, *J*_C–F_ = 21 Hz, C-3″), 113.64 (C-3′/5′), 57.56 (C-5), 41.80 (C-4). LC-Q-TOF-MS *m*/*z* calcd for C_16_H_15_FN_4_S [M + H]^+^: 315.1080; Found 315.1100. Elemental analysis (%) calcd for C_16_H_15_FN_4_S: C 61.13, H 4.81, N 17.82, S 10.20; found: C 61.13, H 4.82, N 17.82, S 10.21.

##### 4.1.2.5 3-(4-Aminophenyl)-5-(3-fluorophenyl)-4,5-dihydro-1*H*-pyrazole-1-carbotioamide (11)

Yield: 69%. White solid, M.p.: 290–292 °C. Rf: 0.70 (diethyl ether). FT-IR (cm^–1^): 3418 (−NH_2_), 1627 (C=N), 1399 (C=S). ^1^H NMR (400 MHz, DMSO-*d*_6_, ppm): δ, 7.90 (bs, −SH); 7.67 (bs, −NH); 7.54 (d, *J* = 8.0 Hz, 2H, H-2′/6′); 7.39–7.33 (m, 1H, H-6″); 7.06 (app t, *J* = 8.0 Hz, 1H, H-5″); 6.96 (d, *J* = 4.0 Hz, 1H, H-2″); 6.89 (d, *J* = 12.0 Hz, 1H, H-4″); 6.57 (d, *J* = 8.0 Hz, 2H, H-3′/5′); 5.88 (d, *J* = 8.0 Hz, 1H, H-5); 5.76 (s, 2H, Ar–NH_2_); 3.82–3.75 (dd, *J*_BA_= 16.0 Hz, *J*_BX_= 12.0 Hz, 1H, H-4b); 3.08–3.03 (dd, *J*_AB_= 16 Hz, *J*_AX_= 4.0 Hz, 1H, H-4a). ^13^C NMR (100 MHz, DMSO-*d*_6_, ppm): 175.47 (C=S), 163.88, 161.46 (d, *J*_C–F_ = 242 Hz, C-3″), 156.28 (C-3), 151.98 (C-4′), 146.56, 146.48 (d, *J*_C–F_ = 8 Hz, C-1″), 130.08, 131.00 (d, *J*_C–F_ = 8 Hz, C-5″), 129.25 (C-2′/6′), 121.74 (C-6″), 117.89 (C-1′), 114.17, 113.98 (d, *J*_C–F_ = 19 Hz, C-2″), 113.66 (C-3′/5′), 112.76, 112.54 (d, *J*_C–F_ = 22 Hz, C-4″), 62.39 (C-5), 42.70 (C-4). LC-Q-TOF-MS *m*/*z* calcd for C_16_H_15_FN_4_S [M + H]^+^: 315.1080; Found 315.1108. Elemental analysis (%) calcd for C_16_H_15_FN_4_S: C 61.13, H 4.81, N 17.82, S 10.20; found: C 61.12, H 4.81, N 17.82, S 10.22.

##### 4.1.2.6 3-(4-Aminophenyl)-5-(4-fluorophenyl)-4,5-dihydro-1*H*-pyrazole-1-carbotioamide (12)

Yield: 73%. White solid, M.p.: 251–253 °C (lit.:316 °C).^63^ Rf: 0.60 (diethyl ether). FT-IR (cm^–1^): 3477, 3380 (−NH_2_), 3352 (−NH_2_, H-bonded), 1576 (C=N), 1362 (C=S). ^1^H NMR (400 MHz, DMSO-*d*_6_, ppm): δ= 7.86 (bs, −SH), δ= 7.64 (bs, −NH), δ= 7.54 (d, *J* = 8.0 Hz, 2H, H-2′/6′); δ= 7.14 (d, *J* = 8.0 Hz, 4H, H-2″/6″/3″/5″); δ= 6.58 (d, *J* = 12.0 Hz, 2H, H-3′/5′); δ= 5.85 (d, *J* = 8.0 Hz, 1H, H-5); δ= 5.76 (s, 2H Ar–NH_2_), δ= 3.81–3.74 (dd, *J*_BA_= 20.0 Hz, *J*_BX_= 12.0 Hz, 1H, H-4b); δ= 3.05–3.00 (dd, *J*_AB_= 16 Hz, *J*_AX_= 4.0 Hz, 1H, H-4a). ^13^C NMR (100 MHz, DMSO-*d*_6_, ppm): 175.36 (C=S), 162.72, 160.31 (d, *J*_C–F_ = 241 Hz, C-4″), 156.27 (C-3), 151.95 (C-4′), 139.81 (C-1″), 129.23 (C-2′/6′), 127.89, 127.81 (d, *J*_C–F_ = 8 Hz, C-2″/6″), 117.95 (C-1′), 115.73, 115.52 (d, *J*_C–F_ = 21 Hz, C-3″/5″), 113.65 (C-3′/5′), 62.18 (C-5), 42.80 (C-4). LC-Q-TOF-MS *m*/*z* calcd for C_16_H_15_FN_4_S [M + H]^+^: 315.1080; Found 315.1076. Elemental analysis (%) calcd for C_16_H_15_FN_4_S: C 61.13, H 4.81, N 17.82, S 10.20; found: C 61.12, H 4.81, N 17.82, S 10.21.

#### 4.1.3 Pharmacology

Pharmacological experiments, including cell culture preparation, cell proliferation (MTT) assay, cytotoxic activity assay, microdilution assay, and DNA binding studies, are detailed in the Supporting Information. The methods for calculating IC_50_ values and percentage inhibition are also provided.

#### 4.1.4 *In Silico* Studies

Molecular docking studies were performed to determine the amino acid residues in the active site of compounds **1**, **2**, **4**, and **10**, which interact with anticancer crystal structures in Table 3, via *in silico* approaches, and to calculate the binding parameters. Schrödinger Maestro 14.1 software (Schrödinger Release 2024–3: Glide, LLC, New York, USA)^64–66^ was used in all molecular docking studies. Compounds **1**, **2**, **4**, and **10** were optimized using the LigPrep wizard (Schrödinger Release 2024–3: LigPrep)^64–66^ utility of the software Schrödinger 2024–3 (Schrödinger, LLC, New York, USA). With this method, a net negative change in substituents was produced in each case using possible tautomeric states Epic at pH 7.0 ± 2.0.

Osteosarcoma is the most common type of malignant bone cancer and has a high metastatic capacity.^67,68^ MG63 is one of the cells that exhibit cytotoxic effects against osteosarcoma cells. SW1353 cells are a type of human chondrosarcoma cell line.^69^ The activity of SW1353 cells is investigated by targeting MMP-13 protein expression.^70,71^ Molecular docking analysis has been identified as a target for MMP-13, as it is a critical tool in bone cancer pathogenesis.^72^ Caspase-3 was chosen as another target because it would induce apoptosis in bone cancer cell lines by suppressing MMP-13 expression, where caspase-3 would also be involved.^73,74^ In the study by Zhan et al., EGFR, caspase-3, and estrogen receptor were identified as the best target proteins, ranked according to their expected baseline targets of the PPI network, which are significantly associated with antiosteosarcoma effects.^75^

Crystal structures of the proteins with which compounds interact were obtained from the Protein Data Bank (https://www.rcsb.org/). All crystal structures have different resolution values and binding sites. Crystal structures obtained from the Protein Data Bank, respectively, were prepared separately with the “Protein Preparation Wizard” module of Schrödinger Maestro 14.1 software (Schrödinger Release 2024–3: Glide, LLC, New York, USA). Proteins were prepared by sequential processes such as deletion of water molecules, addition of missing side chains and hydrogen atoms, protonation states, assignment of partial charges, optimization, and minimization using the OPLS-2005 force field. After the ligand and proteins were prepared separately, the docking score was calculated by interacting with the ligand docking wizard. Ligands were docked using Schrödinger Maestro 14.1 software (Schrödinger Release 2024–3: Glide, LLC, New York, USA) to investigate the binding modes.

## Supplementary Material



## Notes

Experimental procedures; characterization data of compounds **1–12**; ^1^H and ^13^C NMR spectra; HRMS spectra; and elemental analysis results (PDF)
