# Supplementary material for: Synthesis and Biological Evaluation of New Thiocarbamoylpyrazoline and Chalcone Derivatives on Bone Cancer Cell Lines: In Vitro and In Silico Studies
Source: ACS Omega. 2026 Jun 5;11(24):35375–88. doi: 10.1021/acsomega.6c00603 (PMC13294917; doi:10.1021/acsomega.6c00603)
Supplement: Supplementary file 1 [file ao6c00603_si_001.pdf]

SUPPORTING INFORMATION

Synthesis and Biological Evaluation of New Thiocarbamoylpyrazoline and Chalcone Derivatives on Bone Cancer Cell Lines: In *Vitro* and In *Silico* Studies

Fatma Demir[a], Nuran Kahriman[a]*, Ali Aydın[b], Burçin Türkmenoğlu[c], Emre Mandal[a], Safiye Emirdağ[d]*

[a]*Department of Chemistry, Faculty of Science, Karadeniz Technical University, 61080, Trabzon-TÜRKİYE*
[b]*Department of Basic Medical Science, Faculty of Medicine Bozok University, 66900, Yozgat-TÜRKİYE*
[c]*Department of Analytical Chemistry, Faculty of Pharmacy, Erzincan Binali Yıldırım University, 24002, Erzincan- TÜRKİYE*
[d]*Department of Chemistry, Faculty of Science, Ege University, 35040, İzmir-TÜRKİYE*

* Email: safiye.emirdag@ege.edu.tr
* Email: nuranyayli@ktu.edu.tr

1. *Experimental*

1.1. *Materials and equipments*

All chemical reagents and solvents used in the synthesis studies, purification and biological activity investigations were high grade commercial products purchased from Merck, Sigma-Aldrich, Acros Organics, and Isolab. Thin-layer chromatography (TLC) and column chromatography were performed on Merck precoated 60 Kieselgel F_{254} analytical aluminum acidic plates and silica gel 60 (0.040-0.063 mm), respectively. All reactions were monitored using TLC. Structure elucidation of the synthesized compounds was performed using a Bruker 400 MHz NMR in $CDCl_3$, DMSO-d_6 with tetramethyl-silane (TMS) as an internal standard. Infrared spectra were obtained using a PerkinElmer 1600FT-IR (4000-400 cm^{-1}) spectrometer. MS analysis was performed using an Agilent 6550 iFunnel high resolution Accurate-Mass QTOF-MS, equipped with an Agilent Dual Jet Stream electrospray ionization (Dual AJS ESI) interface operating in positive ion. Elemental analysis for carbon, hydrogen, nitrogen, and sulphur was performed using a Macro-Cube CHNS analyser. Melting points were determined using a Stuart SMP10 melting point apparatus.

1.2. *Pharmacology*

1.2.1. *Investigation of Anticancer Activities*

1.2.1.1. *Cancer cell lines and cell culture*

In this study, MG63 (ATCC CRL-1427) and SW1353 (ATCC HTB-94) bone cancer cell lines, as well as HC (Sigma Aldrich, 402-05A) normal chondrocyte cell line, were used. All cell culture procedures were performed under sterile conditions in a laminar flow cabinet. The cells were cultured in DMEM supplemented with 10% fetal bovine serum (FBS) and 2% Penicillin-Streptomycin (PenStrep) at 37 °C in a humidified atmosphere containing 5% CO_2. Cells were seeded into 96-well plates at a density of 10,000 cells per well. After approximately 16 hours of pre-incubation, test compounds were added, and measurements were taken following a 24-hour incubation period.

1.2.1.2. *Cell proliferation measurement and determination of NCI-60 survival parameters*

The MTT assay was employed to evaluate the effects of test compounds on cell proliferation and to determine NCI-60 survival parameters. After 24 hours of exposure to the test substances, cell viability was assessed, and results were expressed as percent inhibition. Cells treated with DMSO (solvent control) were considered to represent 100% viability.

NCI-60 survival parameters (GI_{50}, TGI, and LC_{50}) were calculated based on the absorbance values obtained, using logarithmic regression analysis. The following formulas were applied: Cell proliferation: [(Ti-Tz)/(C-Tz)]x100 if Ti>/=Tz (cytostatic effect) or [(Ti-Tz)/Tz]x100 if Ti<Tz (cytotoxic effect) (Tz; zero point, C; control growth, Ti; inhibition by test substance),

GI_{50}: Concentration value that reduces growth by 50% ([(Ti-Tz)/(C-Tz)]x100=50), TGI: Concentration value that reduces growth by 100% (Ti=Tz),

LC_{50}: Concentration value that kills cells in the medium by 50% ([(Ti-Tz)/Tz]x100=-50).

Tumor selectivity index (TSI) was determined according to the following formula;

TSI: TGI concentration averages of normal cells / TGI concentration of cancer cells.

1.2.1.3. Cytotoxicity test

The LDH method was used to determine whether the test compounds were cell cytotoxic or cytostatic. Depending on the tested compounds, the increase in the number of cells that died during the incubation period will cause an increase in LDH in the culture supernatant. Lactate dehydrogenase (LDH) is a stable cytoplasmic enzyme found in most cells. For this purpose, the LDH cell cytotoxicity kit was used according to the manufacturer's procedure. Briefly, the change in the amount of formazan formed as a result of LDH enzyme activity was measured and evaluated according to the following formula;

% Cytotoxicity = [(Substance Absorbance - Low Control / High Control - Low Control) x 100].

1.3. Spectra of compounds

All spectra of the compounds and elemental analysis results are given in Figure S1-Figure S49.

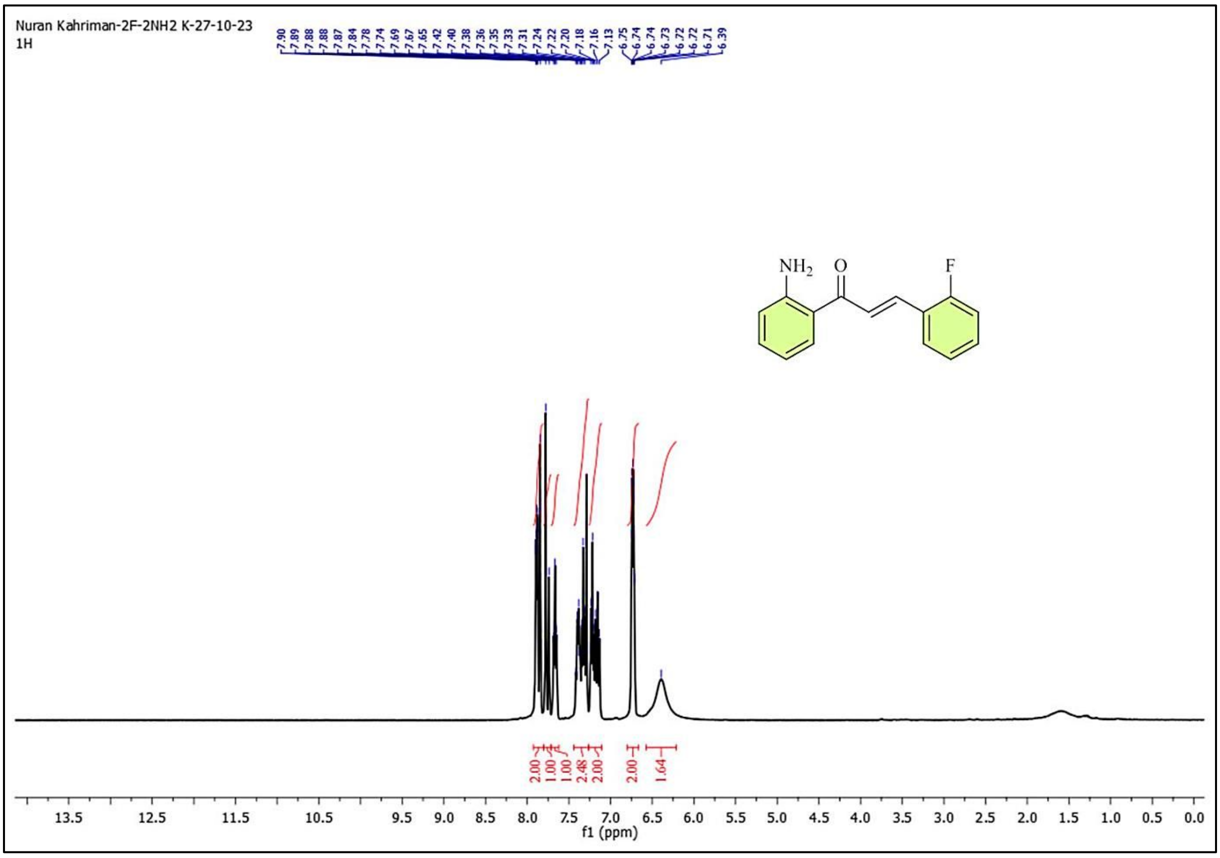

Figure S1. ^{1}H-NMR spectrum of compound **1** (400 MHz, $CDCl_3$, ppm)

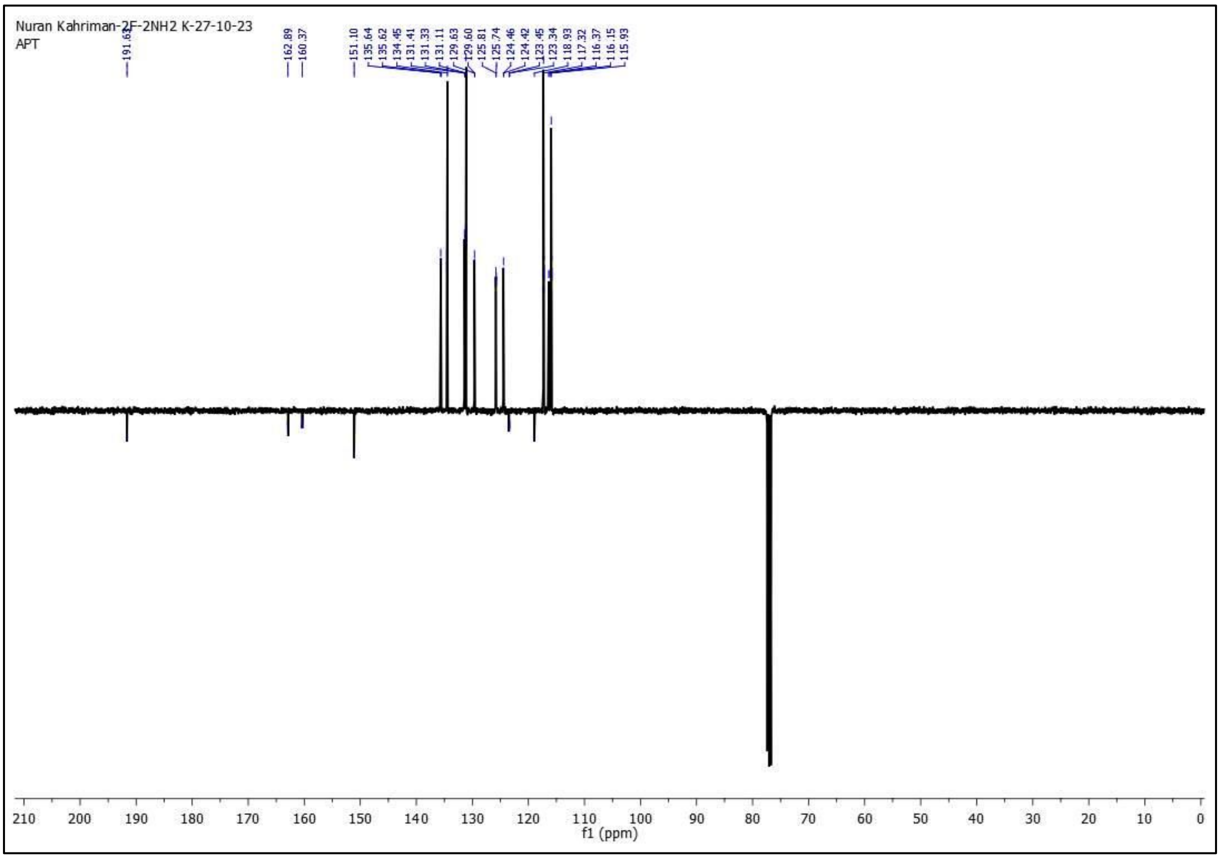

Figure S2. ^{13}C-APT NMR spectrum of compound **1** (100 MHz, $CDCl_3$, ppm)

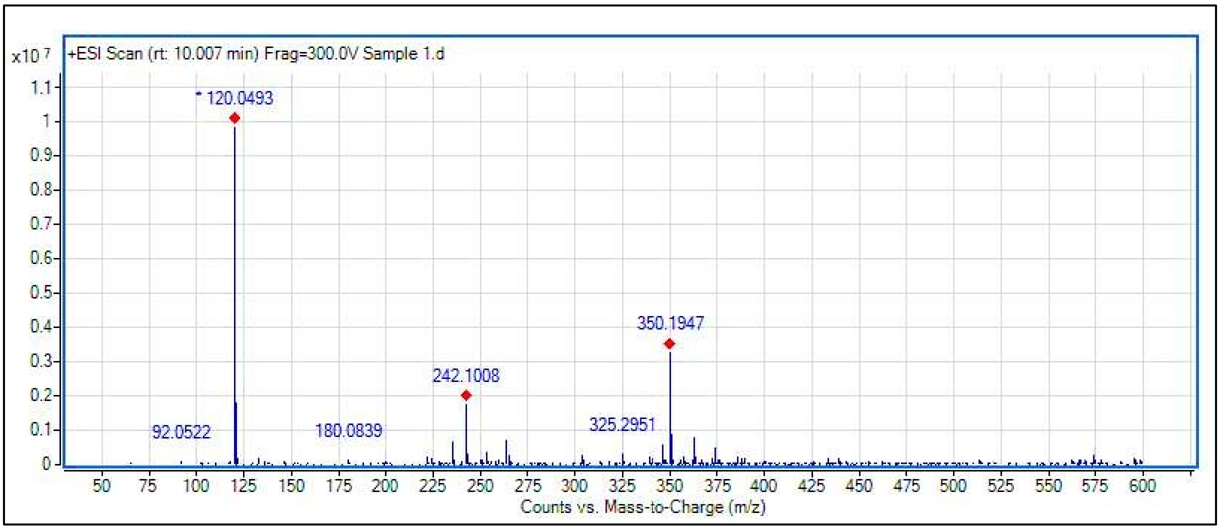

Figure S3. LC-Q-TOF-MS spectrum of compound **1**

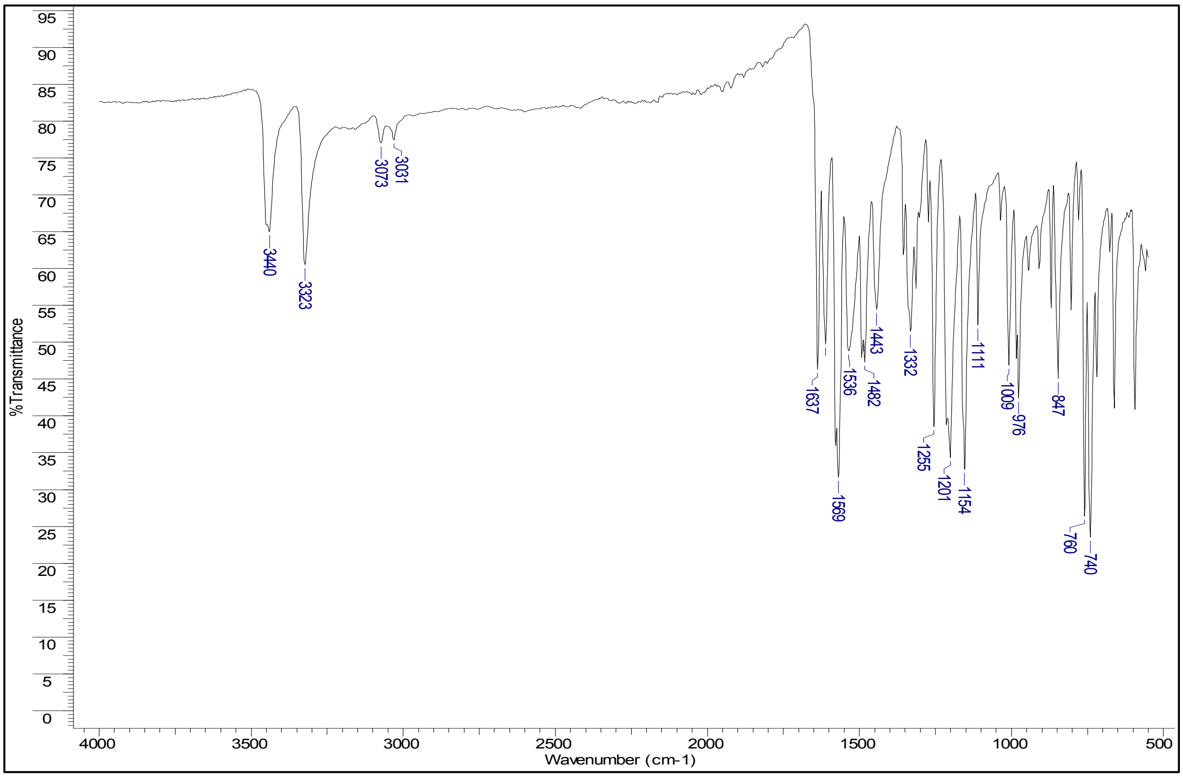

Figure S4. FT-IR spectrum of compound **1** (cm^{-1})

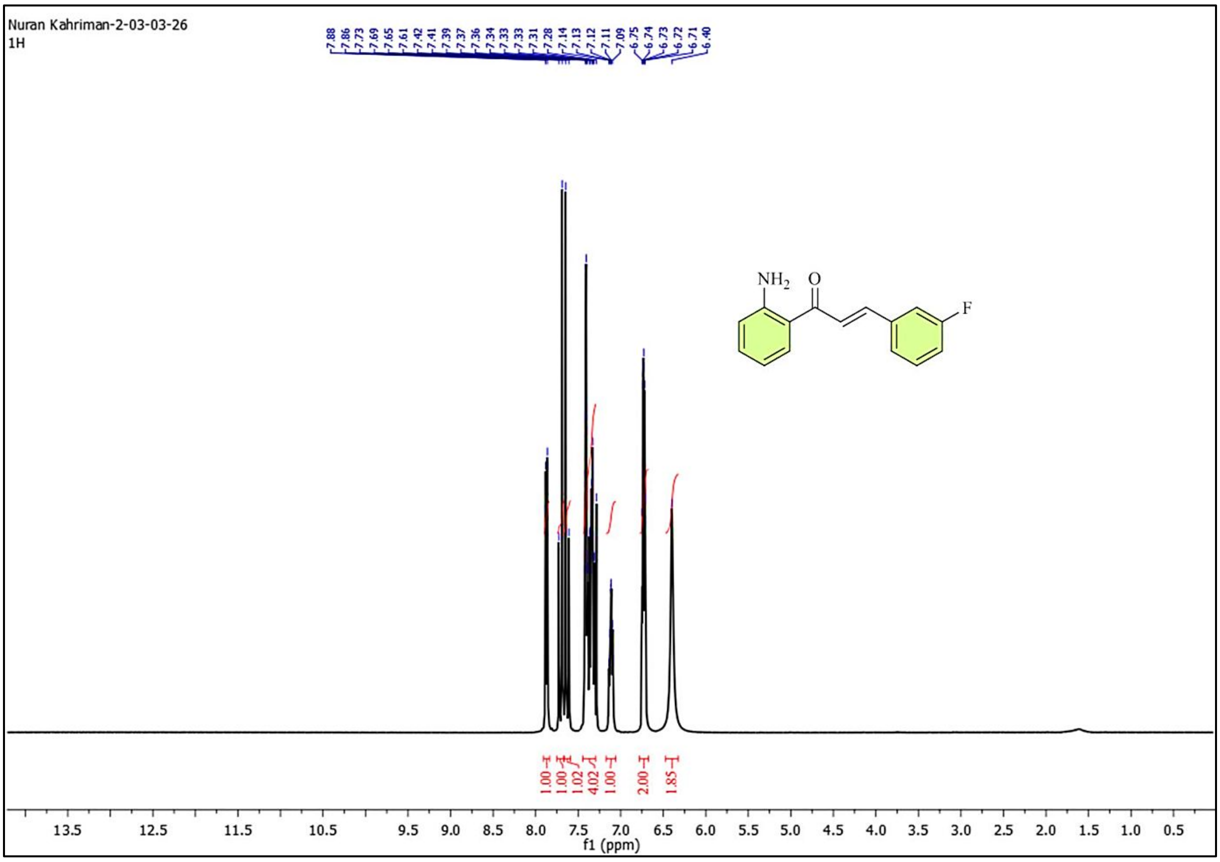

Figure S5. ^{1}H-NMR spectrum of compound **2** (400 MHz, $CDCl_3$, ppm)

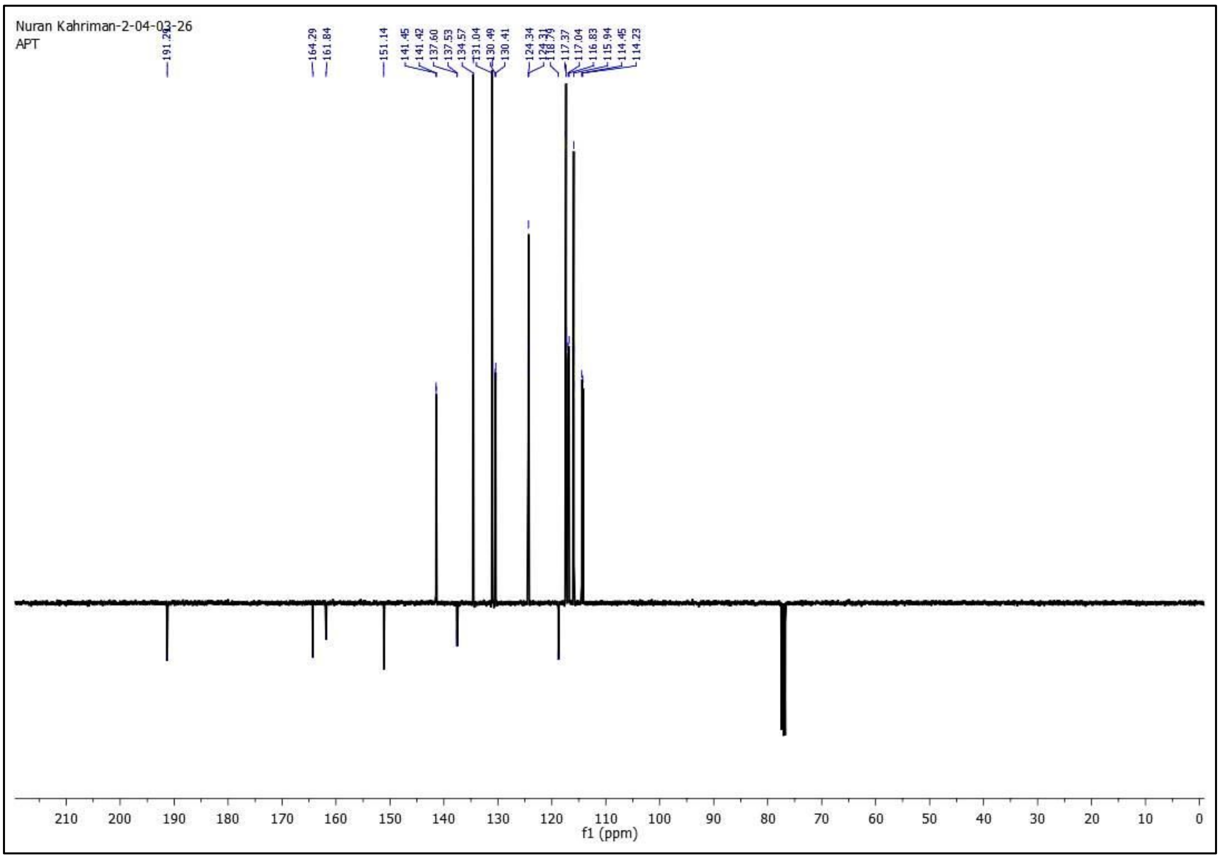

Figure S6.^{13}C-APT NMR spectrum of compound **2** (100 MHz, $CDCl_3$, ppm)

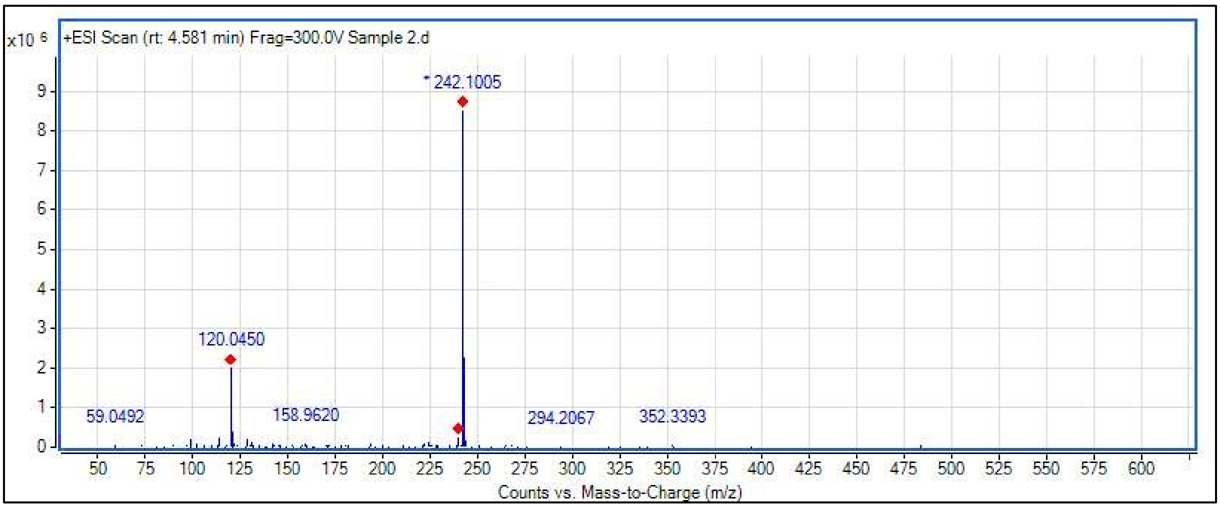

Figure S7. LC-Q-TOF-MS spectrum of compound **2**

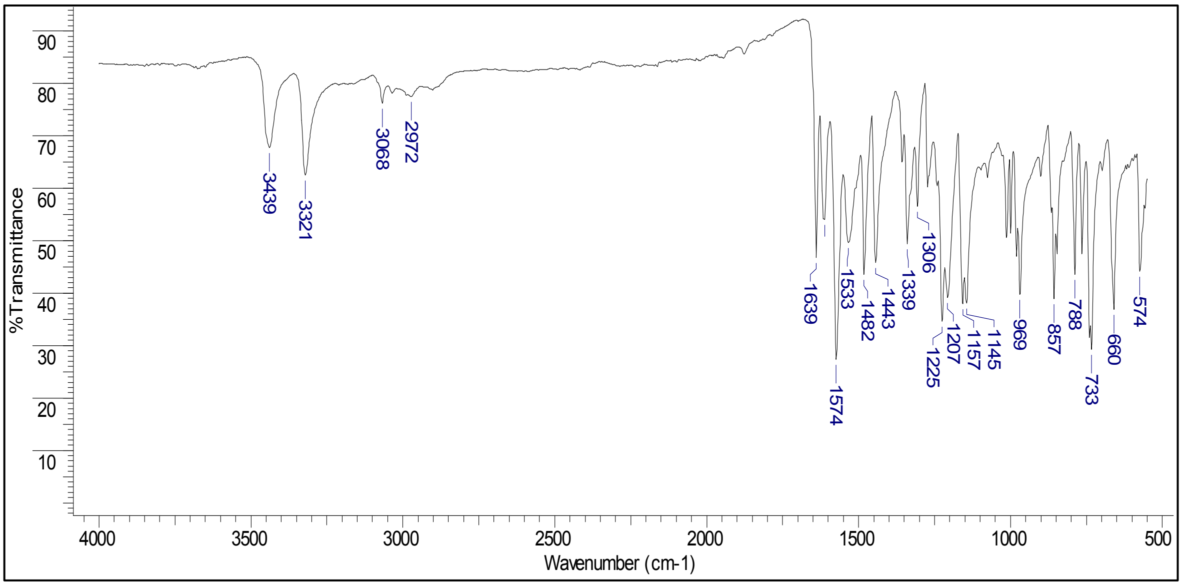

Figure S8. FT-IR spectrum of compound **2** (cm^{-1})

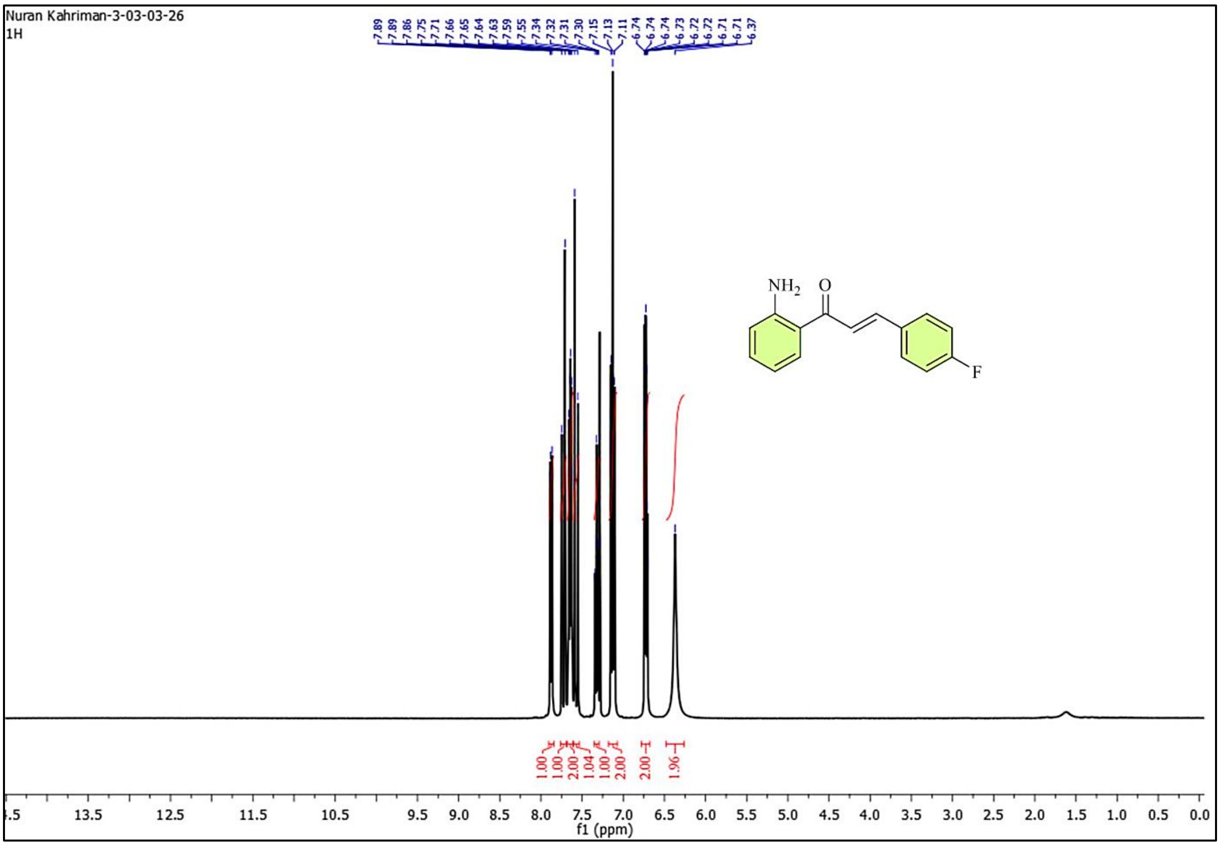

Figure S9. ^{1}H-NMR spectrum of compound **3** (400 MHz, $CDCl_3$, ppm)

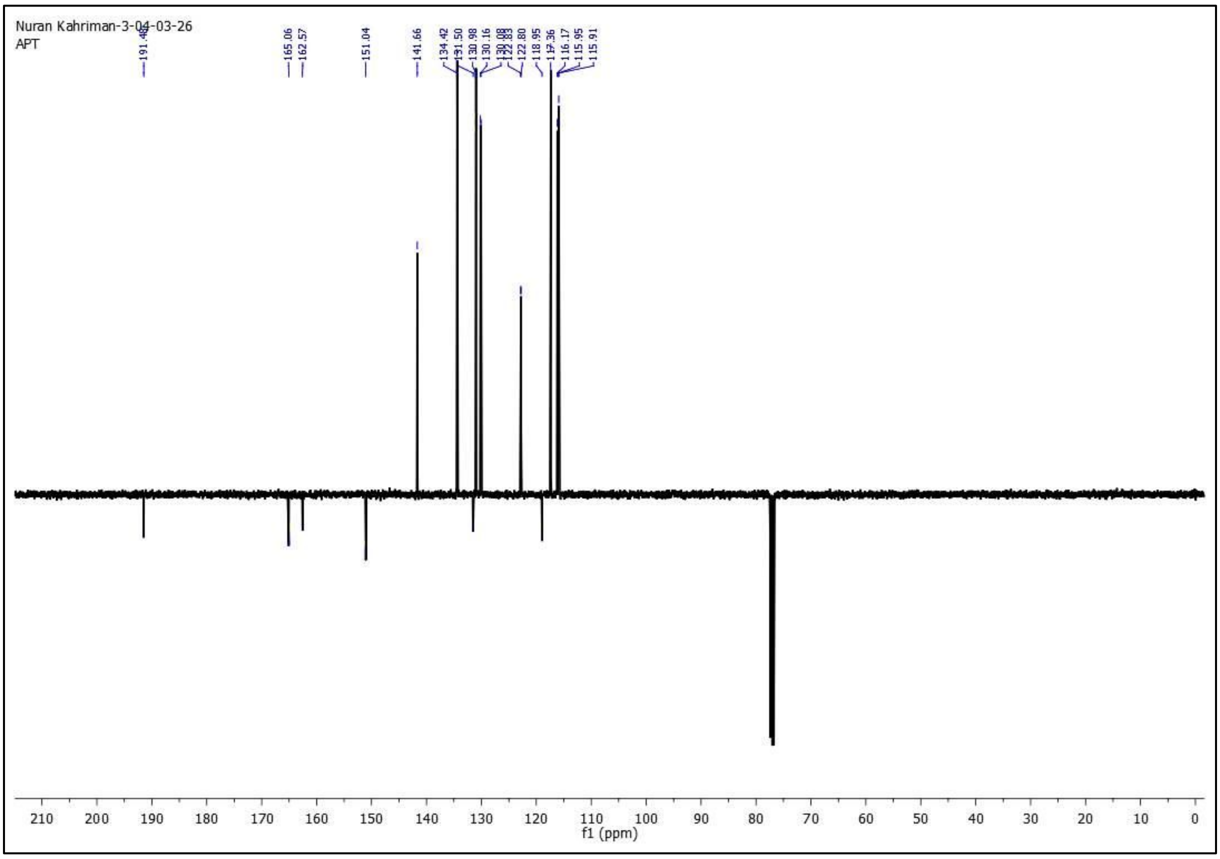

Figure S10.^{13}C-APT NMR spectrum of compound **3** (100 MHz, $CDCl_3$, ppm)

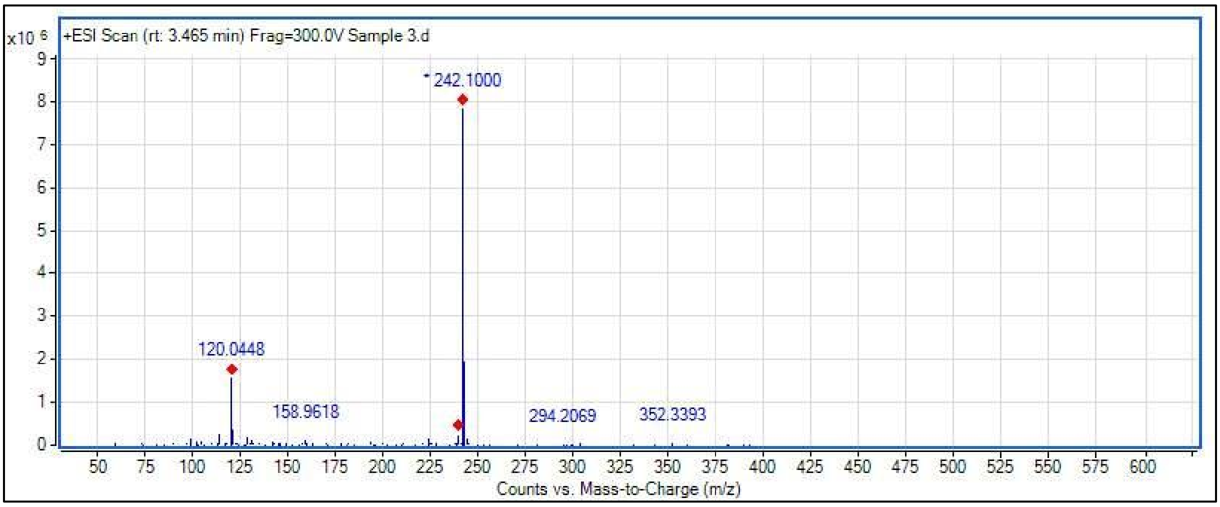

Figure S11. LC-Q-TOF-MS spectrum of compound **3**

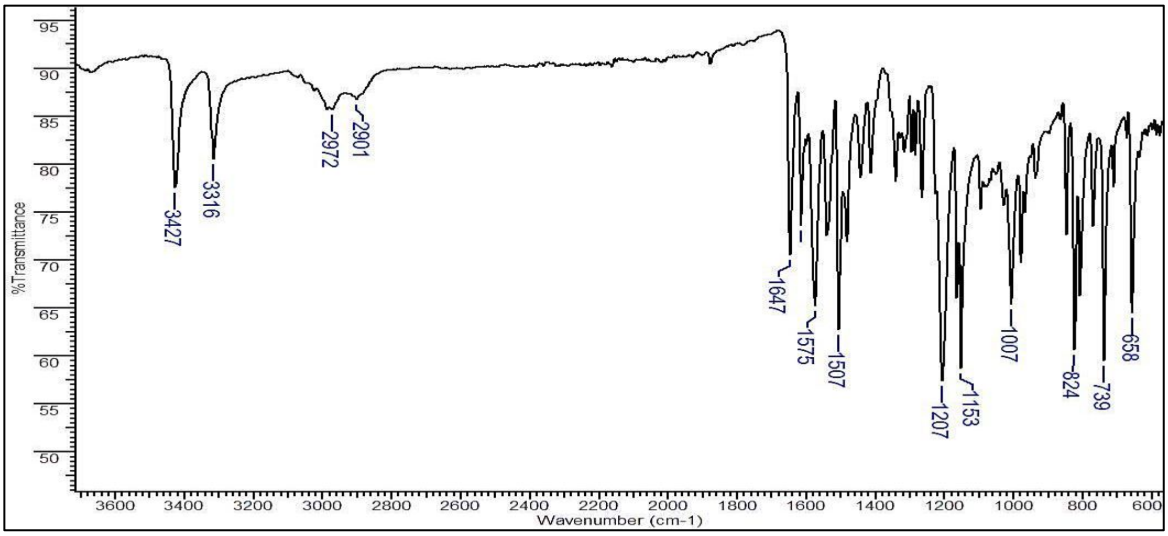

Figure S12. FT-IR spectrum of compound **3** (cm^{-1})

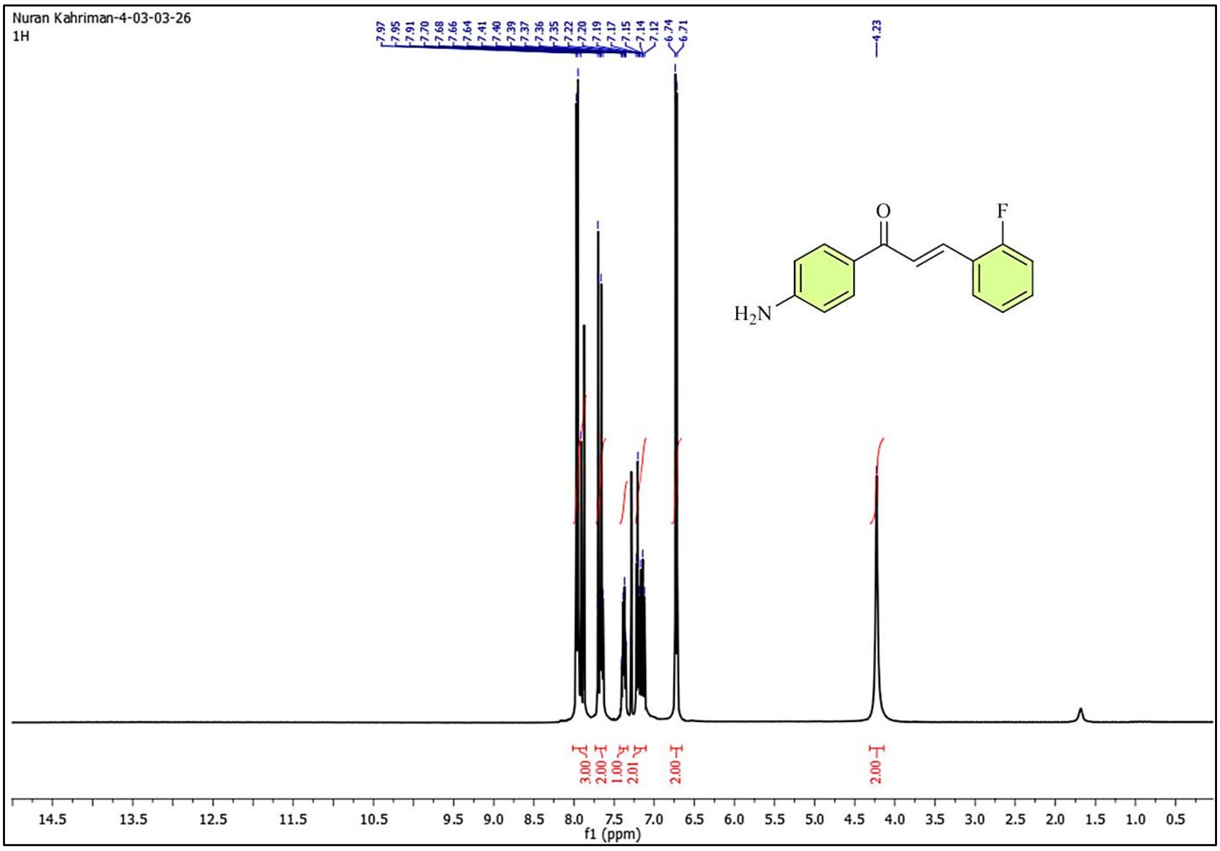

Figure S13. ^{1}H-NMR spectrum of compound **4** (400 MHz, $CDCl_3$, ppm)

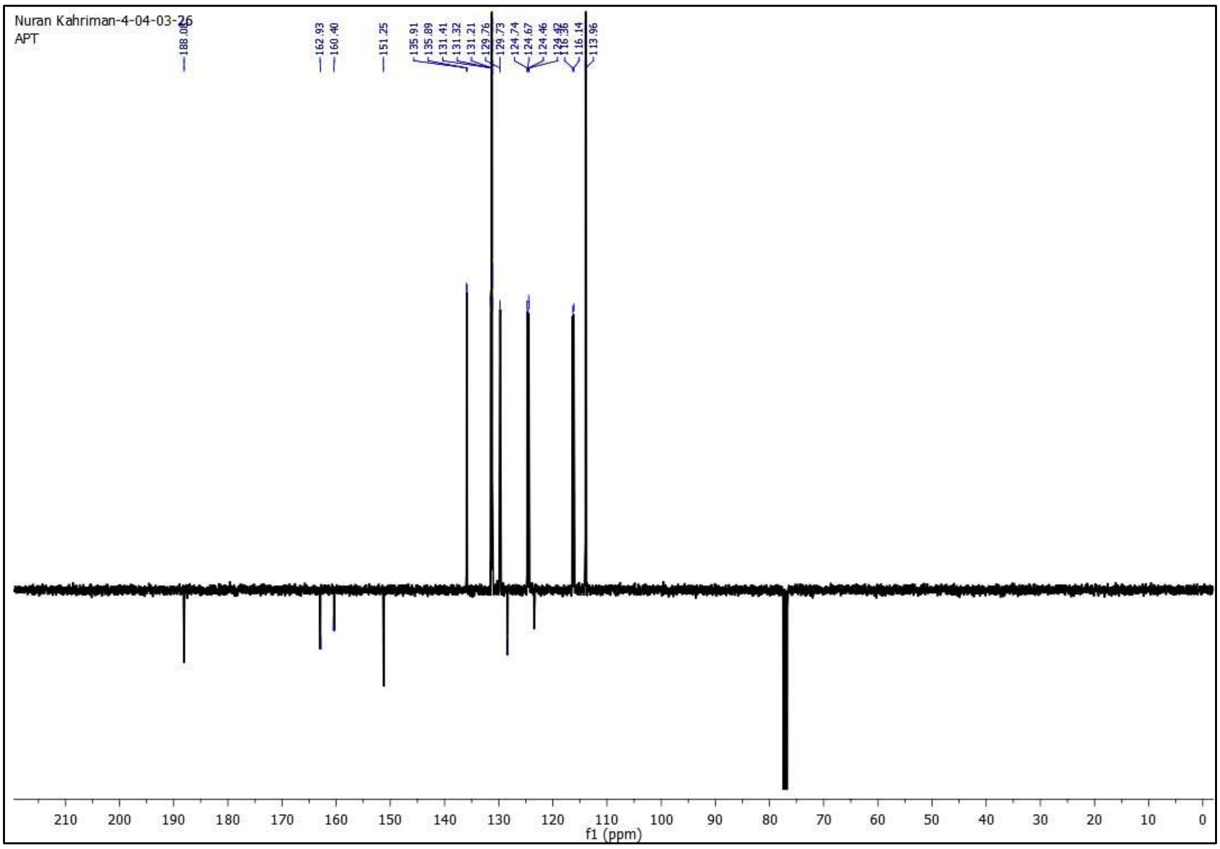

Figure S14.^{13}C-APT NMR spectrum of compound **4** (100 MHz, $CDCl_3$, ppm)

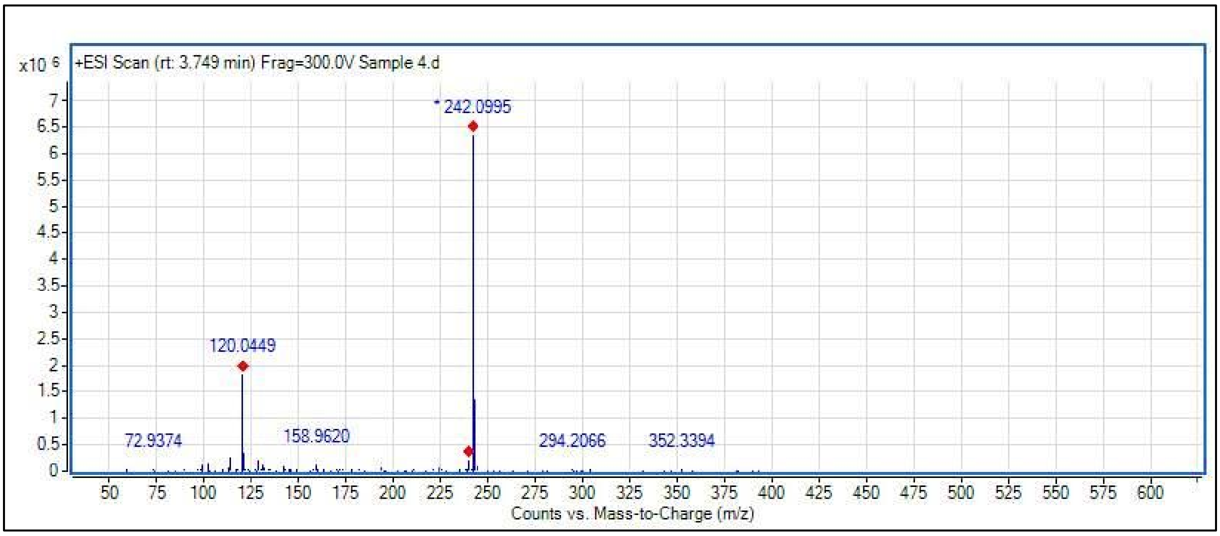

Figure S15. LC-Q-TOF-MS spectrum of compound **4**

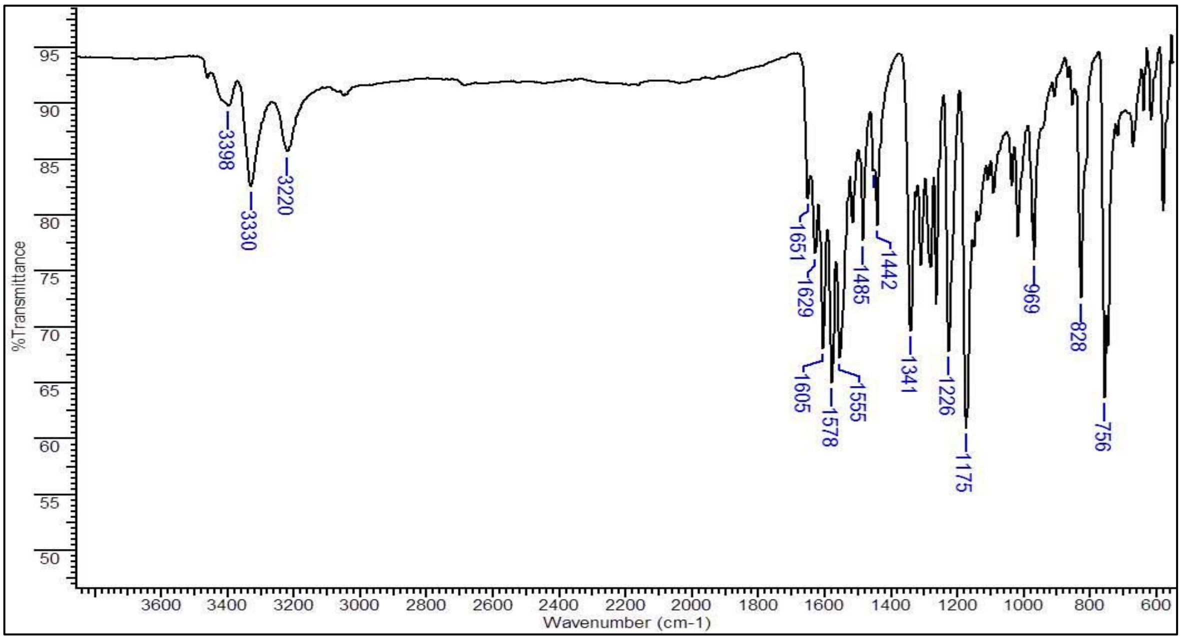

Figure S16. FT-IR spectrum of compound **4** (cm^{-1})

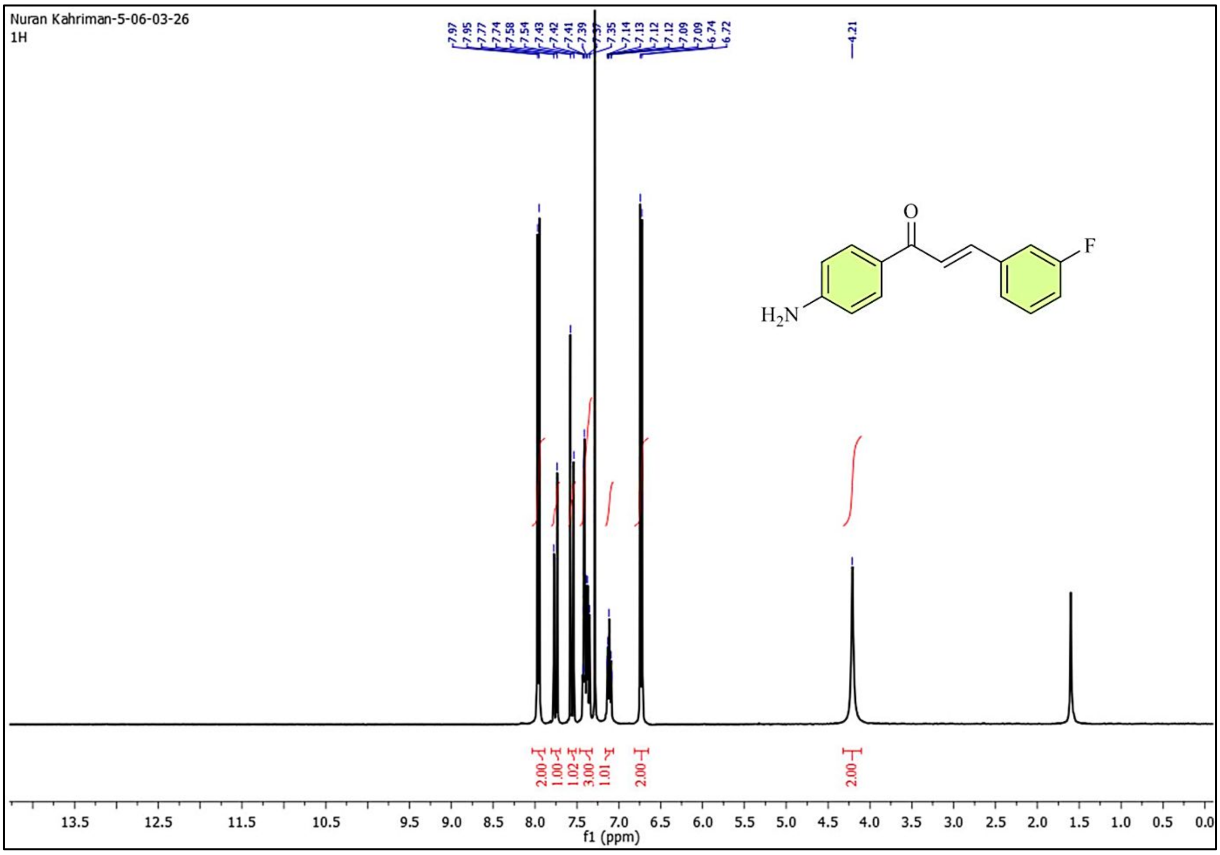

Figure S17. ^{1}H-NMR spectrum of compound **5** (400 MHz, $CDCl_3$, ppm)

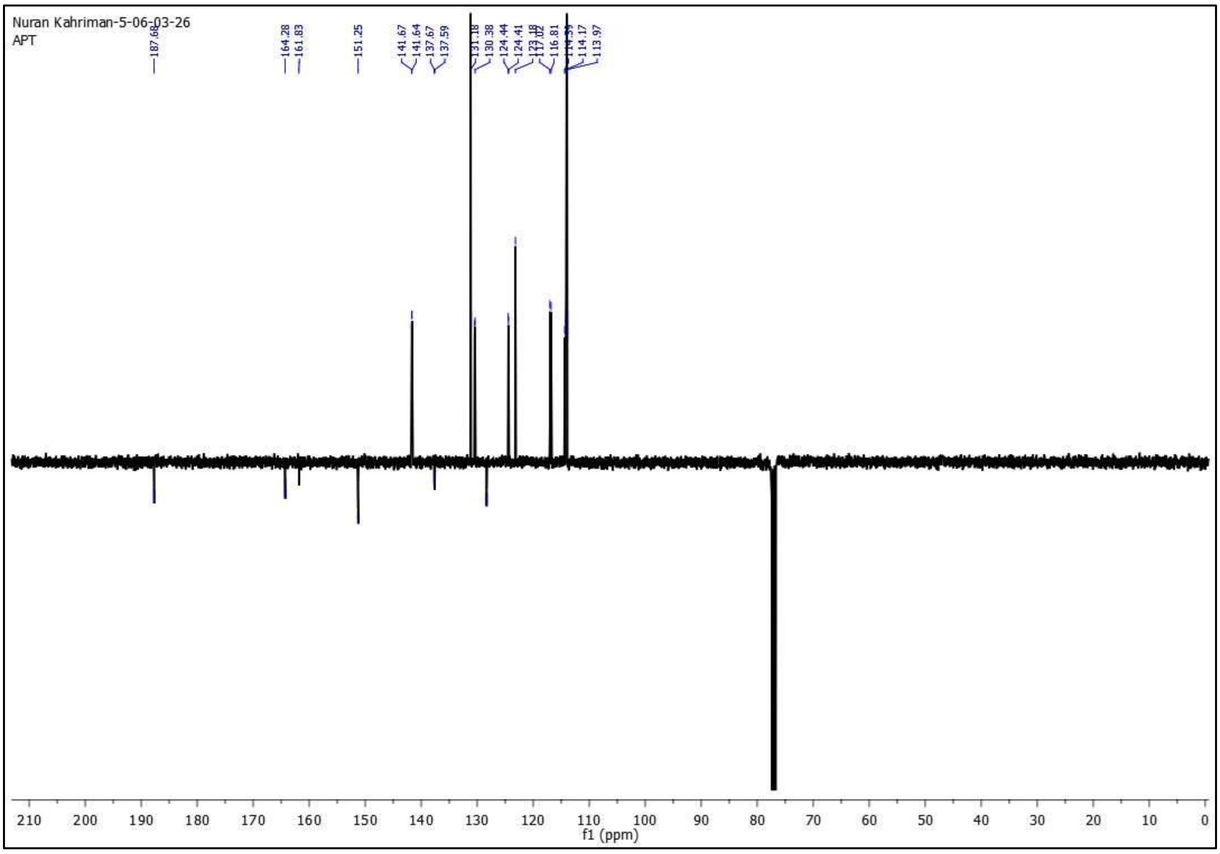

Figure S18. ^{13}C-APT NMR spectrum of compound **5** (100 MHz, $CDCl_3$, ppm)

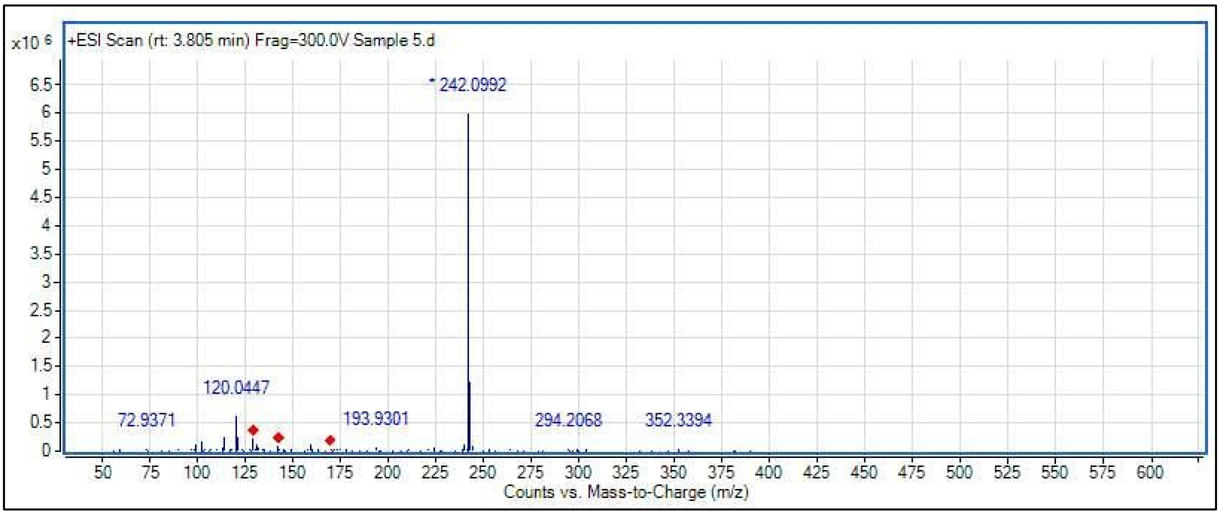

Figure S19. LC-Q-TOF-MS spectrum of compound **5**

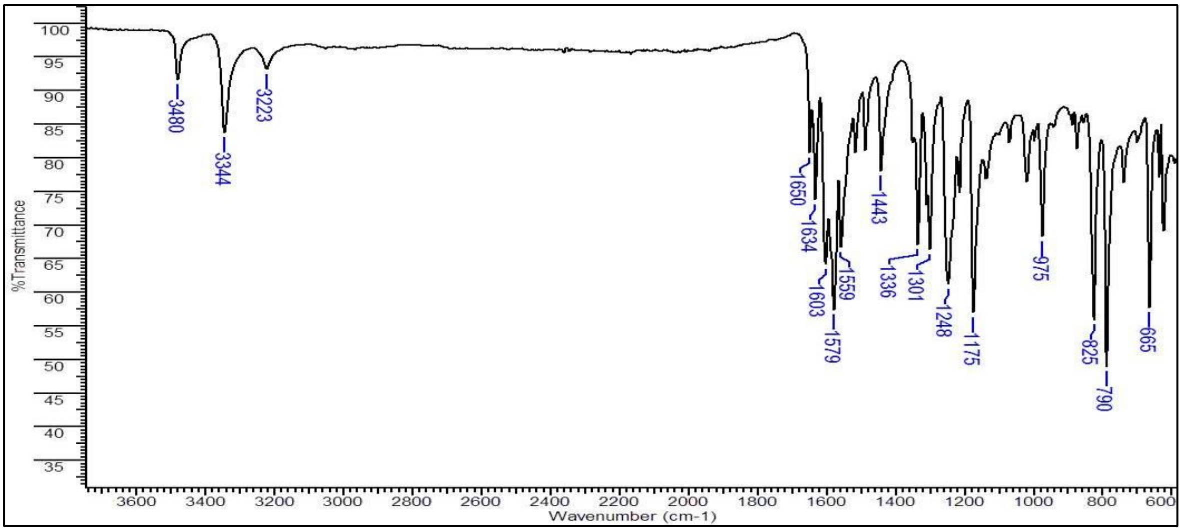

Figure S20. FT-IR spectrum of compound **5** (cm^{-1})

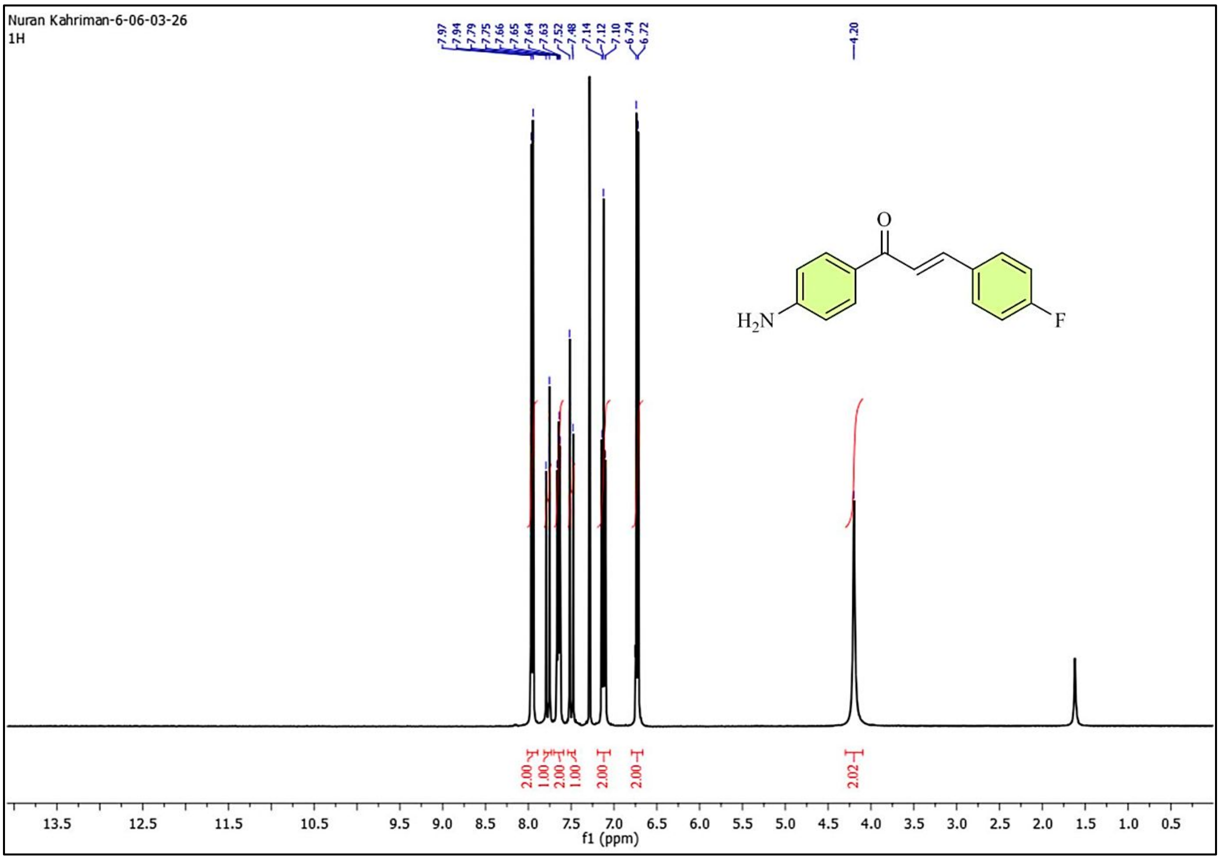

Figure S21. ^{1}H-NMR spectrum of compound **6** (400 MHz, $CDCl_3$, ppm)

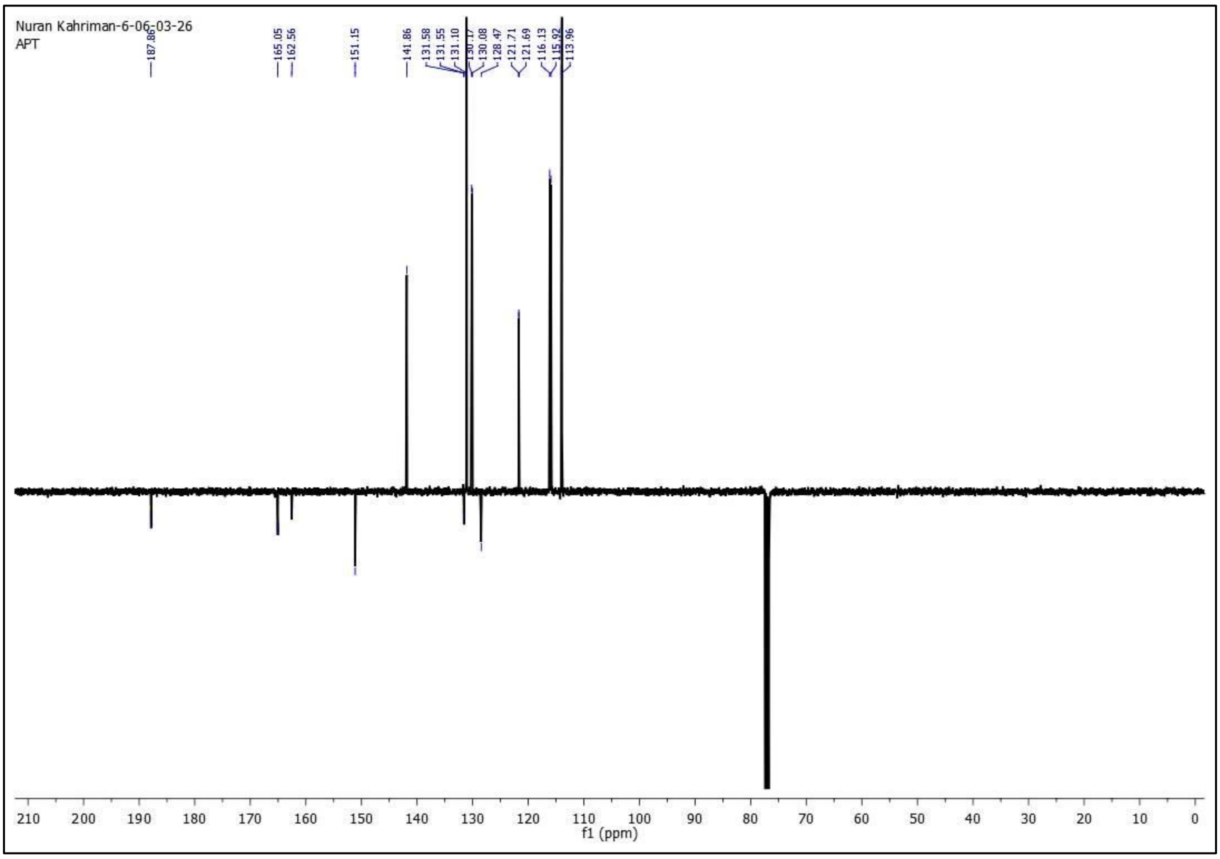

Figure S22.^{13}C-APT NMR spectrum of compound **6** (100 MHz, $CDCl_3$, ppm)

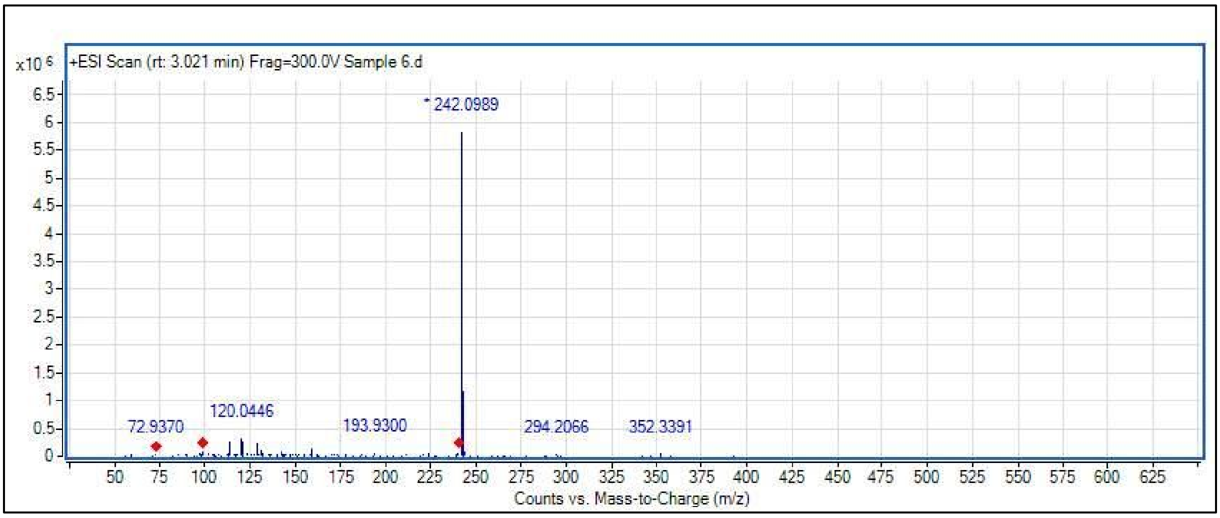

Figure S23. LC-Q-TOF-MS spectrum of compound **6**

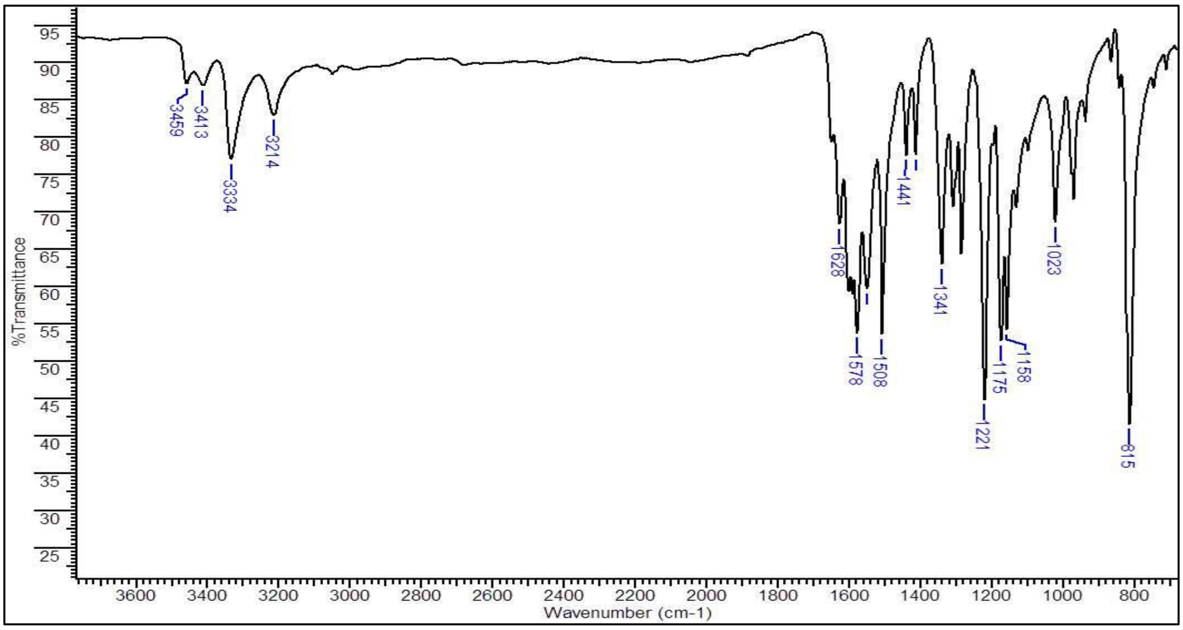

Figure S24. FT-IR spectrum of compound **6** (cm^{-1})

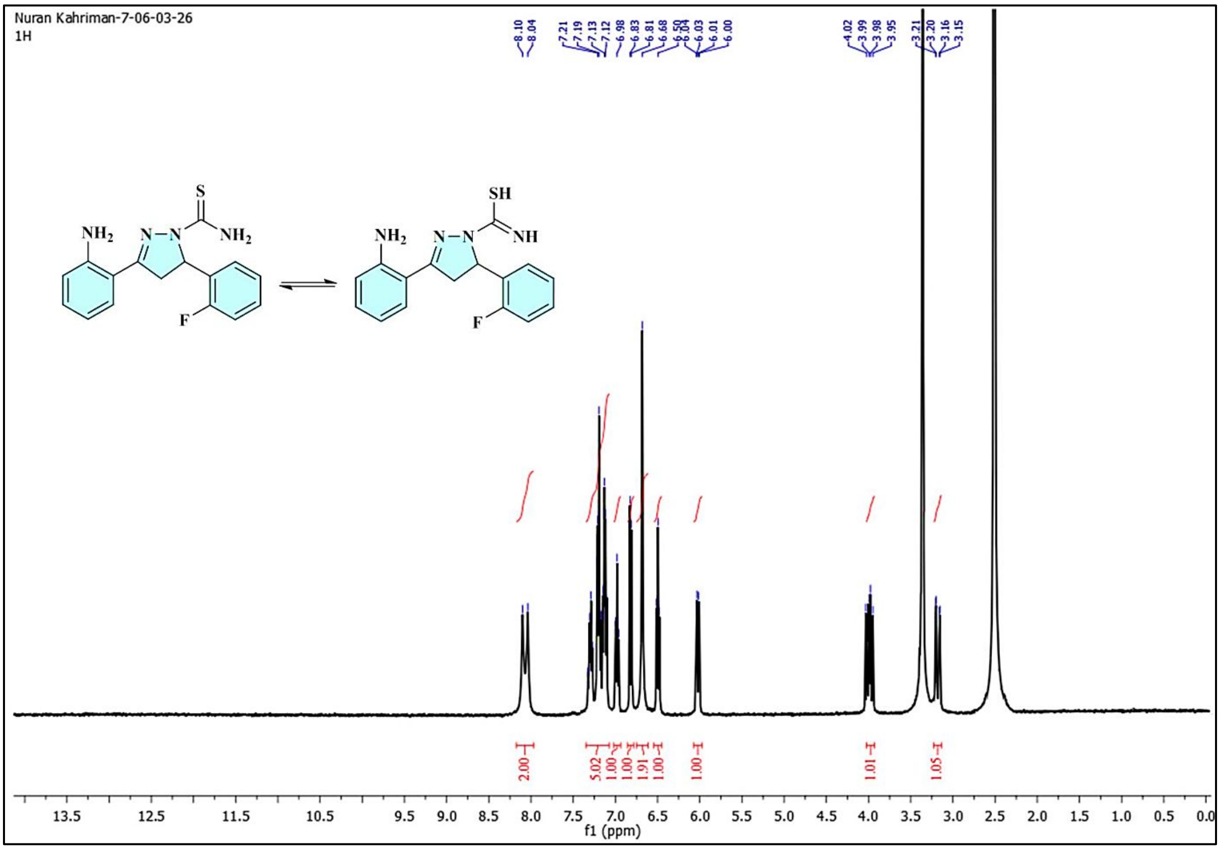

Figure S25. ^{1}H-NMR spectrum of compound **7** (400 MHz, DMSO-d_6, ppm)

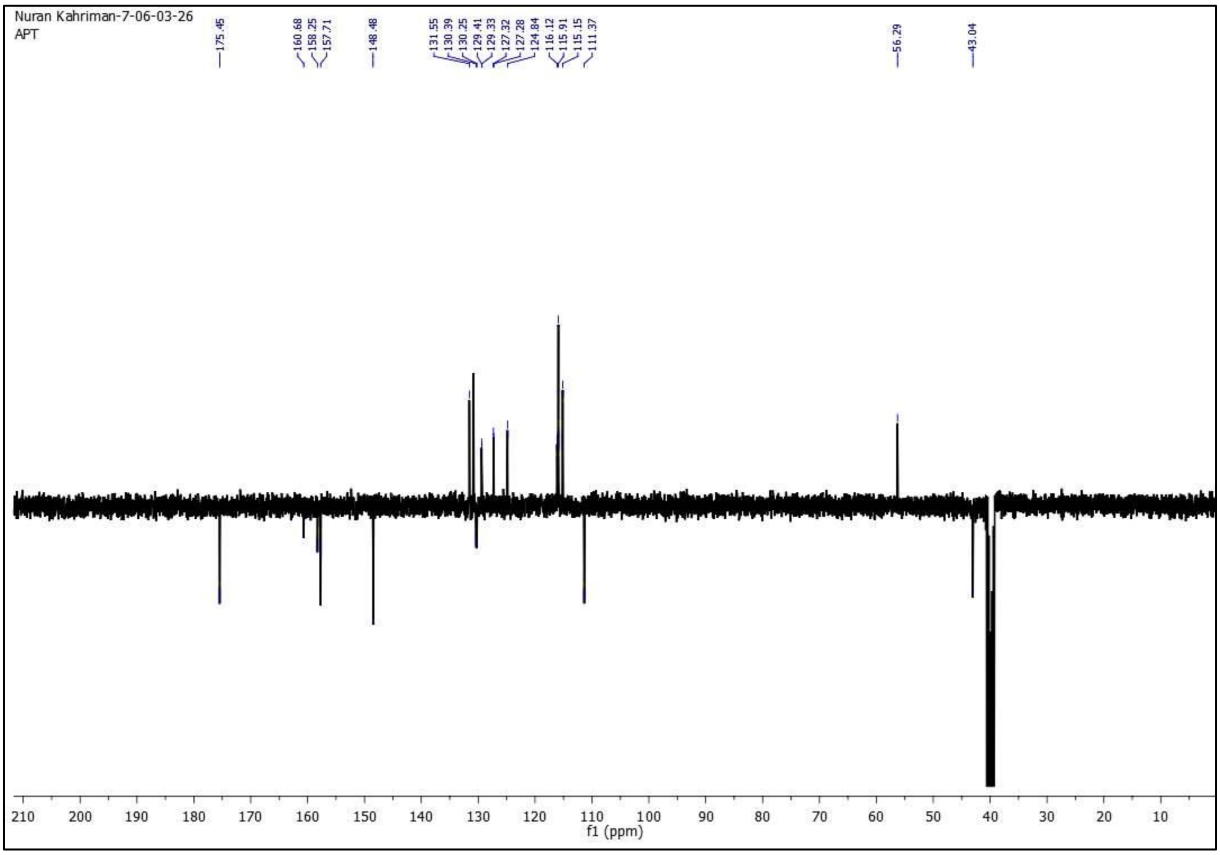

Figure S26.^{13}C-APT NMR spectrum of compound **7** (100 MHz, DMSO-d_6, ppm)

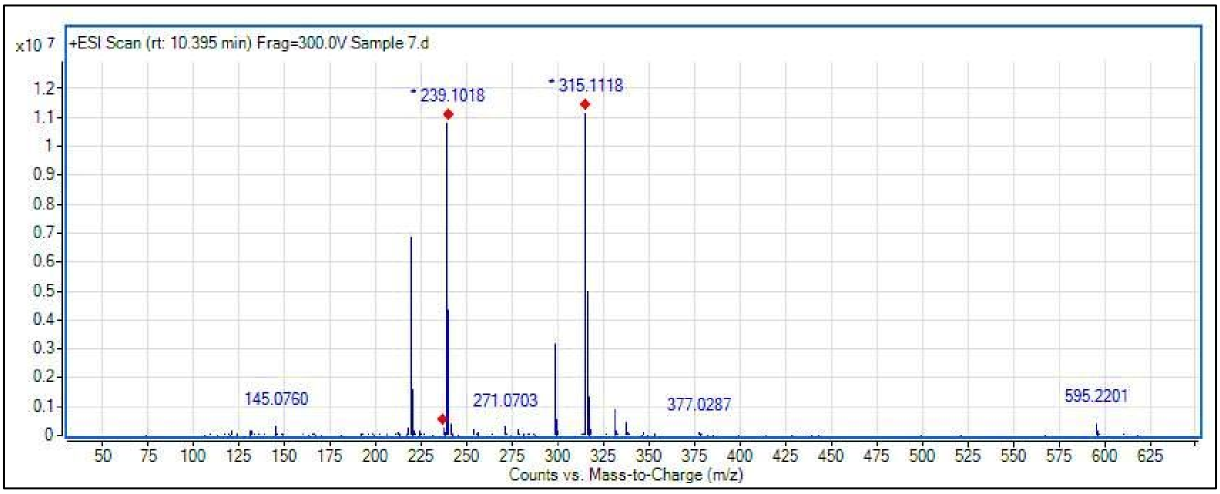

Figure S27. LC-Q-TOF-MS spectrum of compound **7**

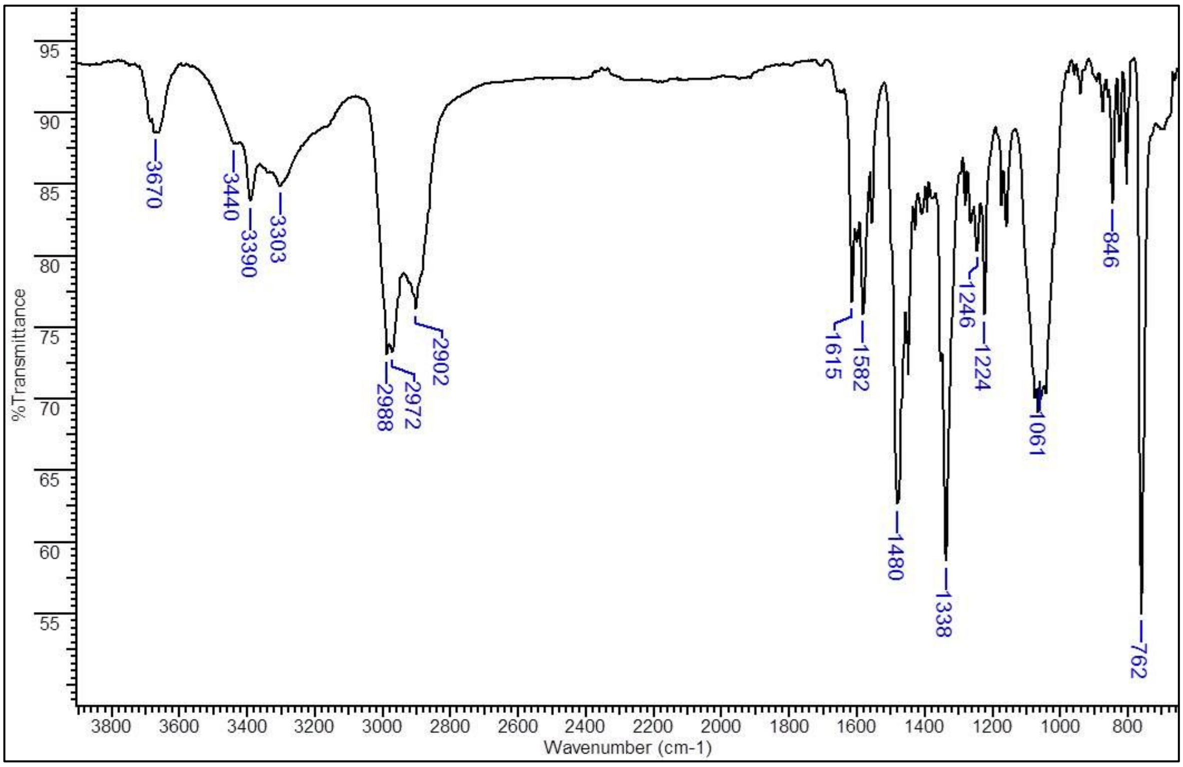

Figure S28. FT-IR spectrum of compound **7** (cm^{-1})

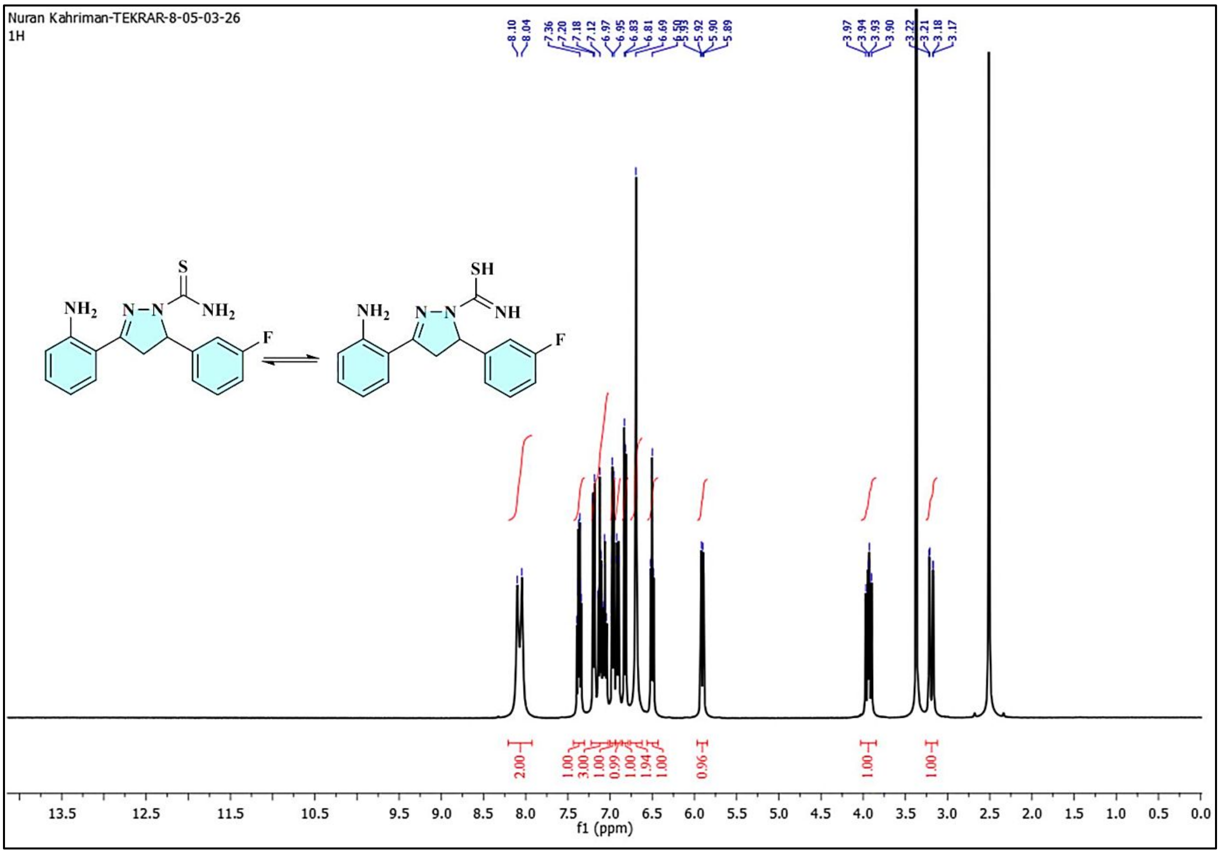

Figure S29. ^{1}H-NMR spectrum of compound **8** (400 MHz, DMSO-d_6, ppm)

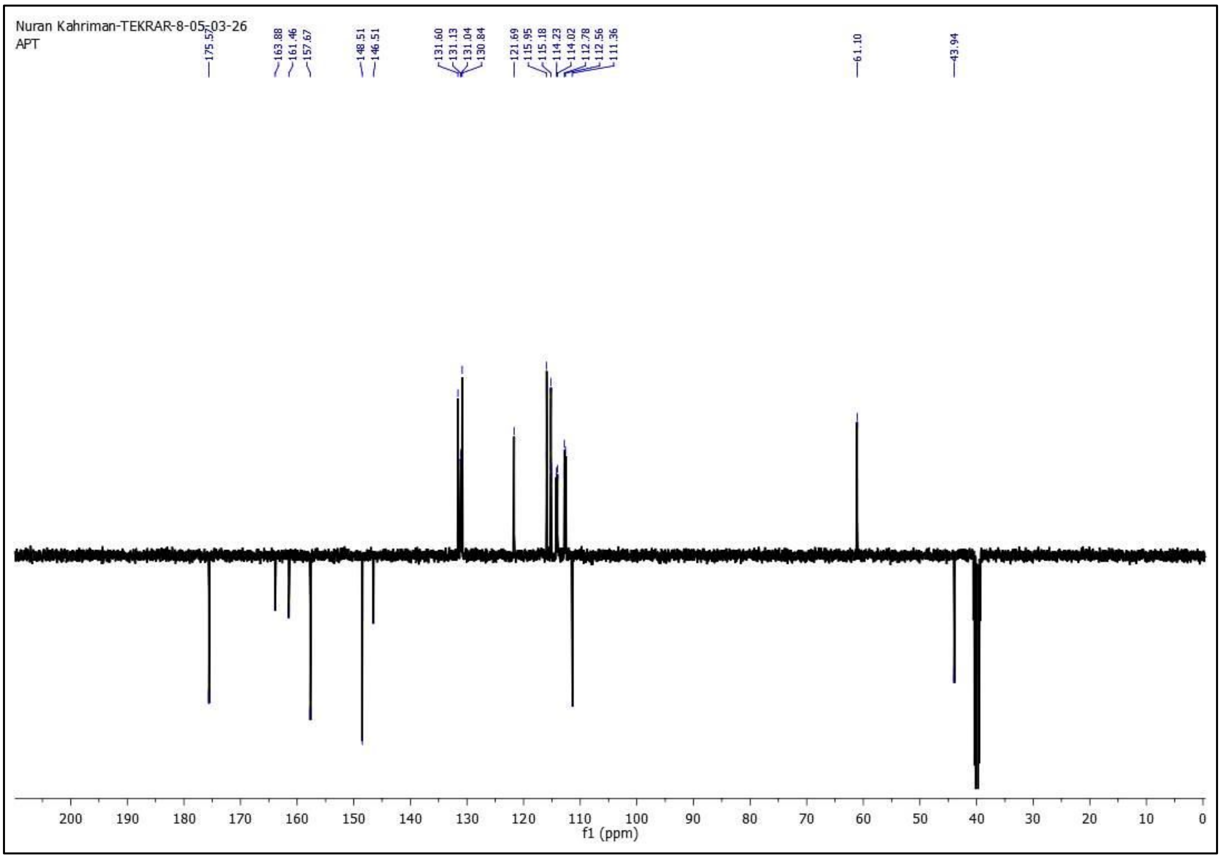

Figure S30.^{13}C-APT NMR spectrum of compound **8** (100 MHz, DMSO-d_6, ppm)

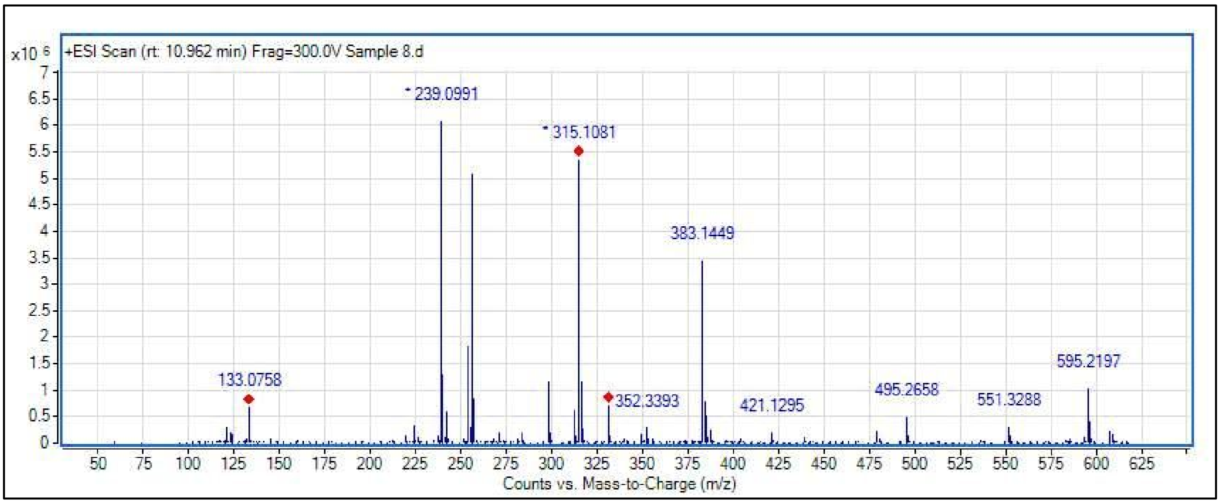

Figure S31. LC-Q-TOF-MS spectrum of compound **8**

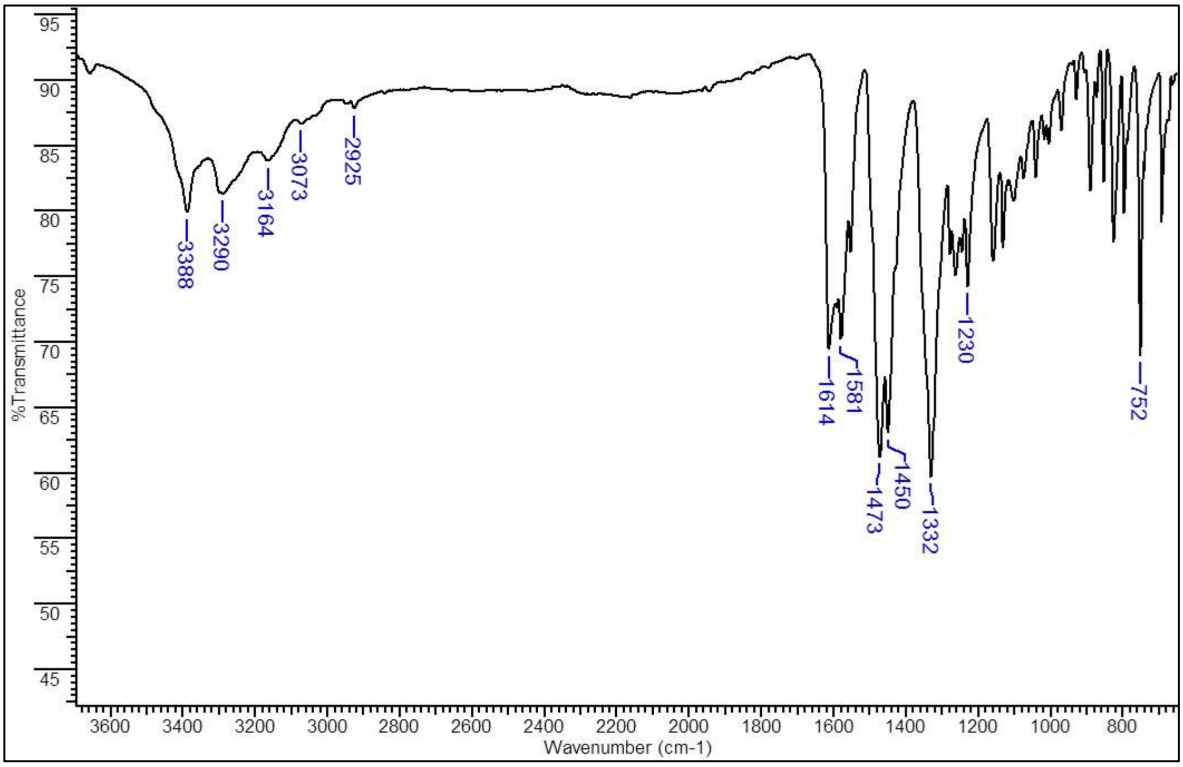

Figure S32. FT-IR spectrum of compound **8** (cm^{-1})

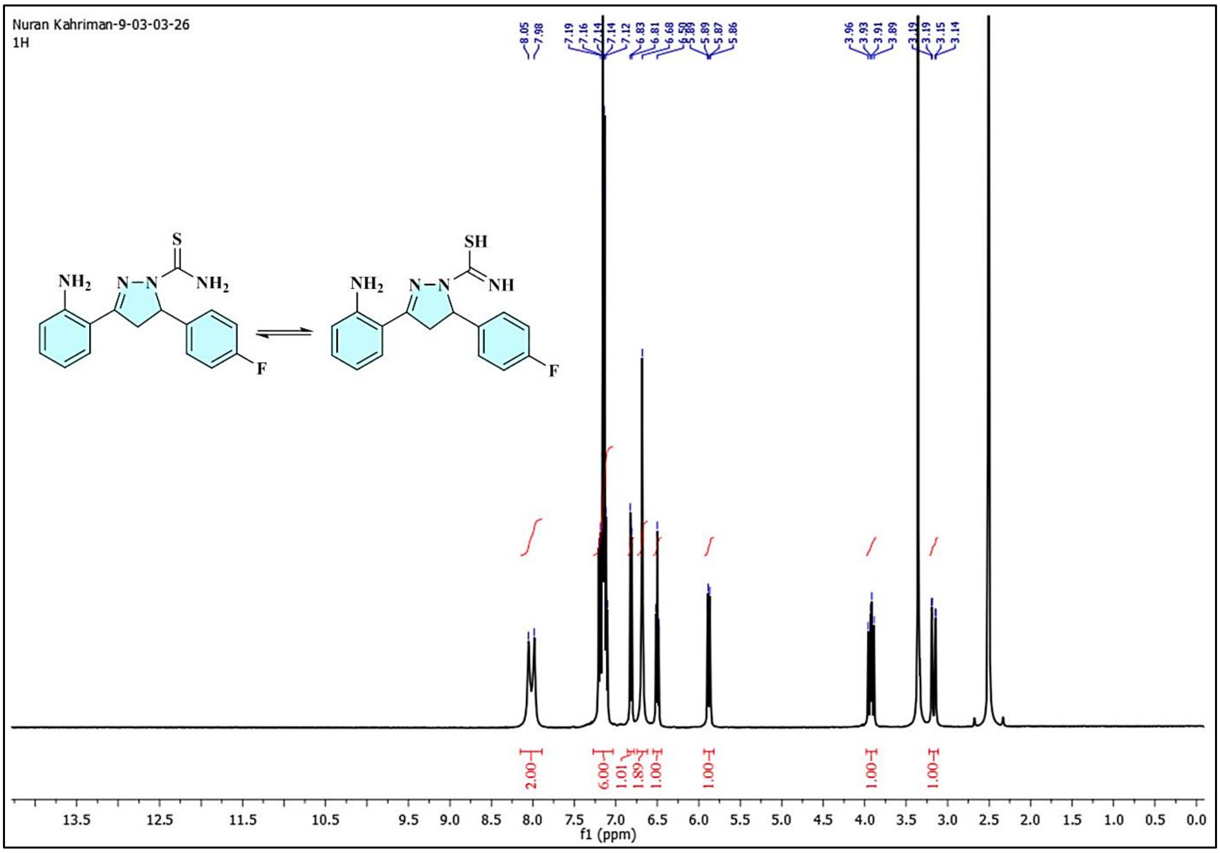

Figure S33. ^{1}H-NMR spectrum of compound **9** (400 MHz, DMSO-d_6, ppm)

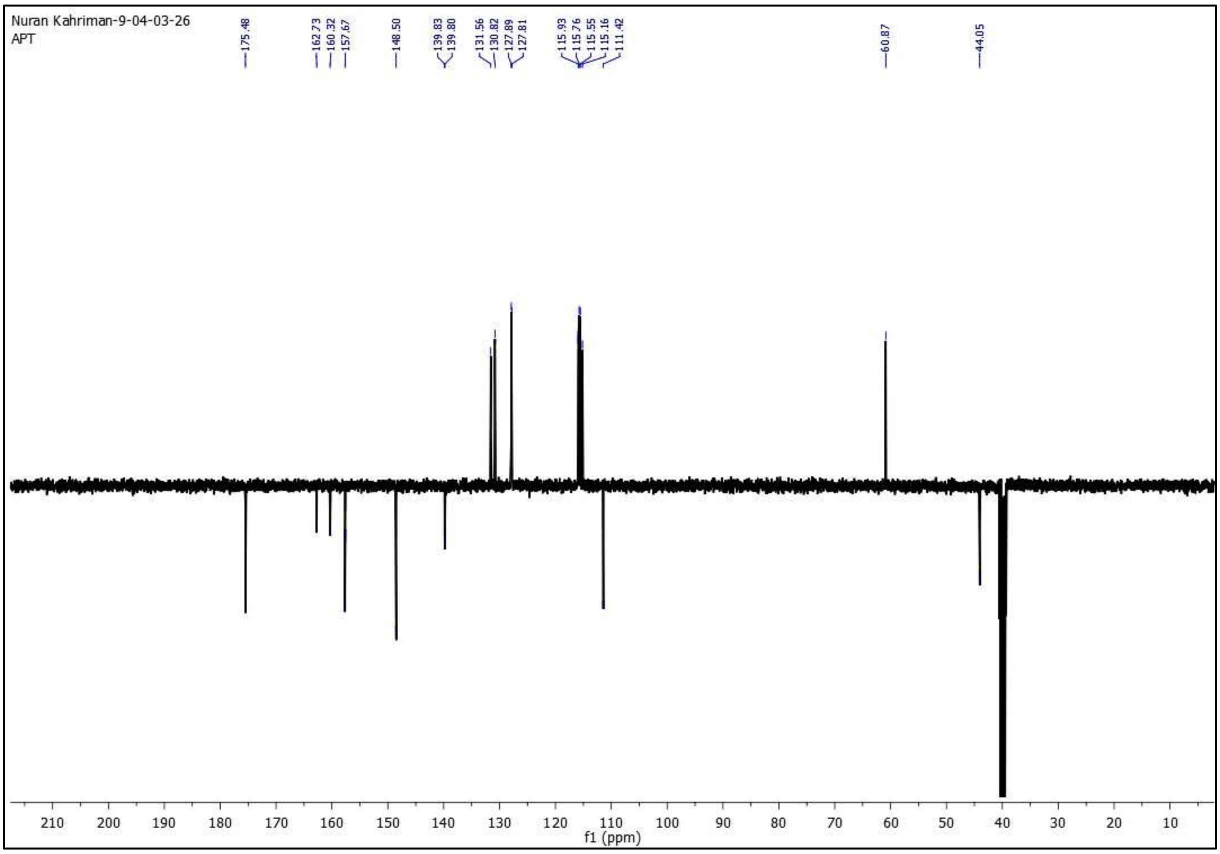

Figure S34.^{13}C-APT NMR spectrum of compound **9** (100 MHz, DMSO-d_6, ppm)

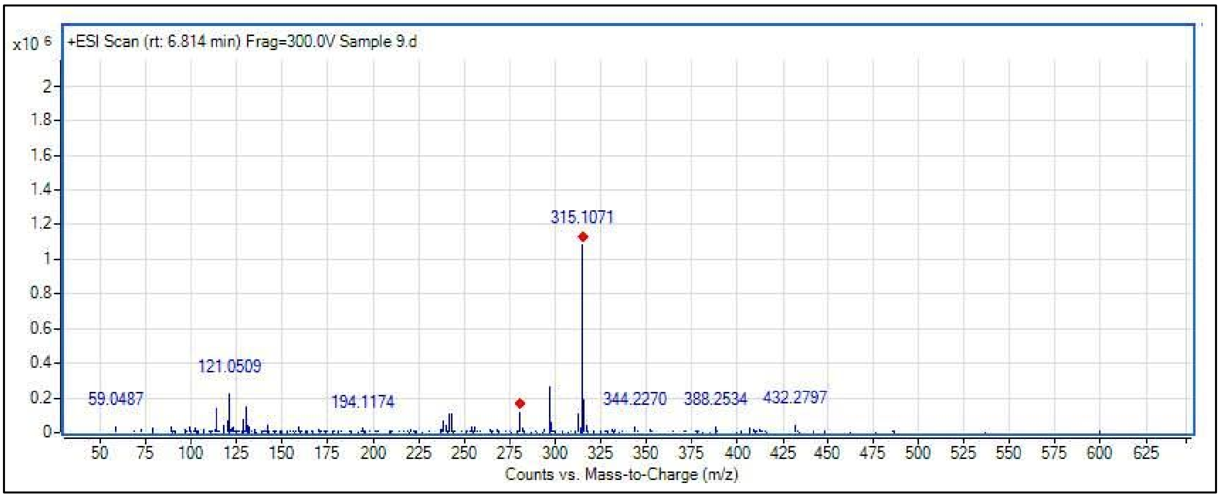

Figure S35. LC-Q-TOF-MS spectrum of compound **9**

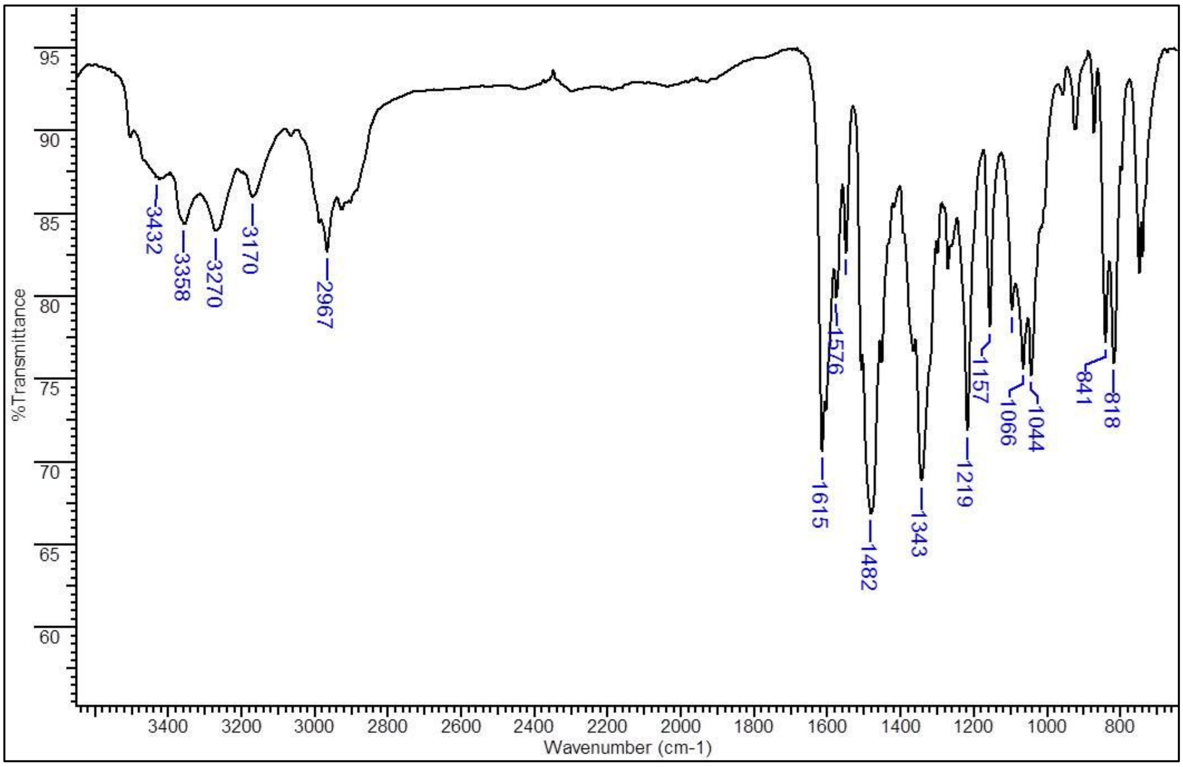

Figure S36. FT-IR spectrum of compound **9** (cm^{-1})

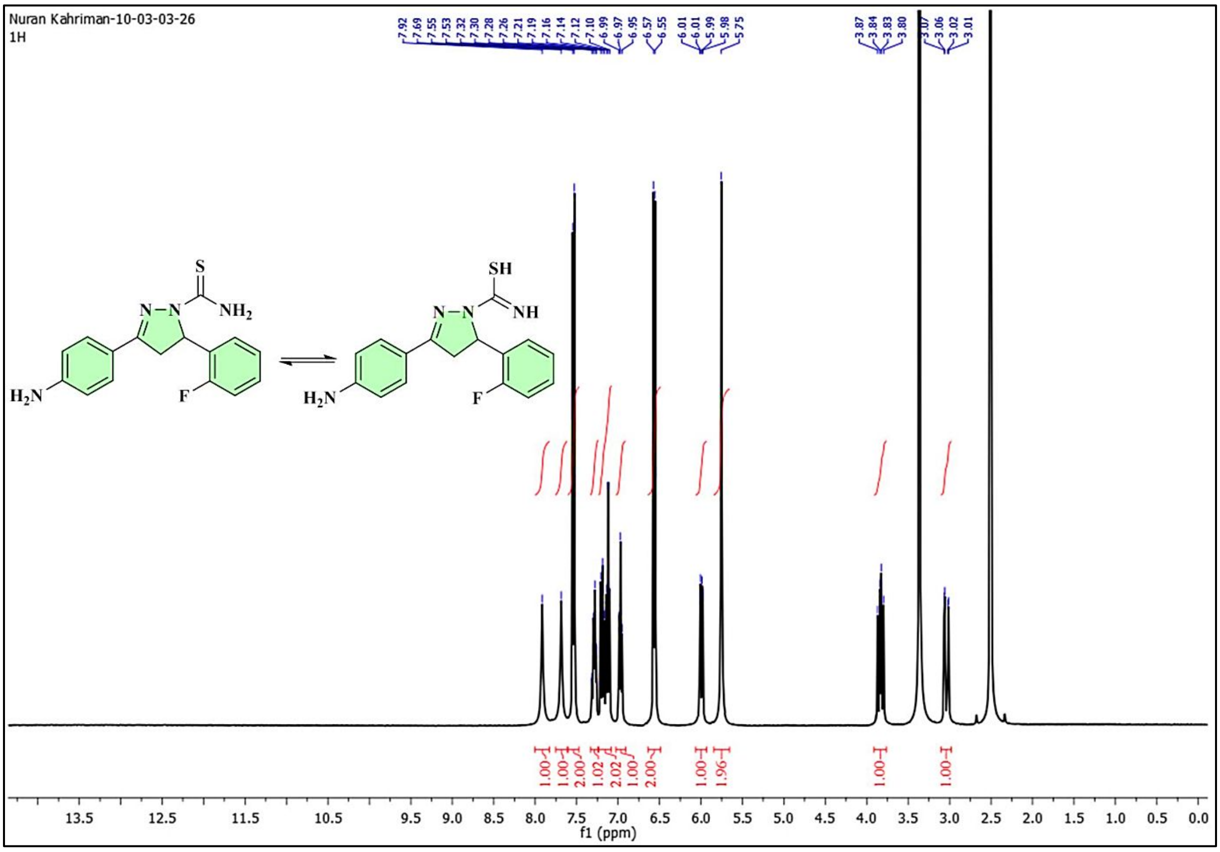

Figure S37. ^{1}H-NMR spectrum of compound **10** (400 MHz, DMSO-d_6, ppm)

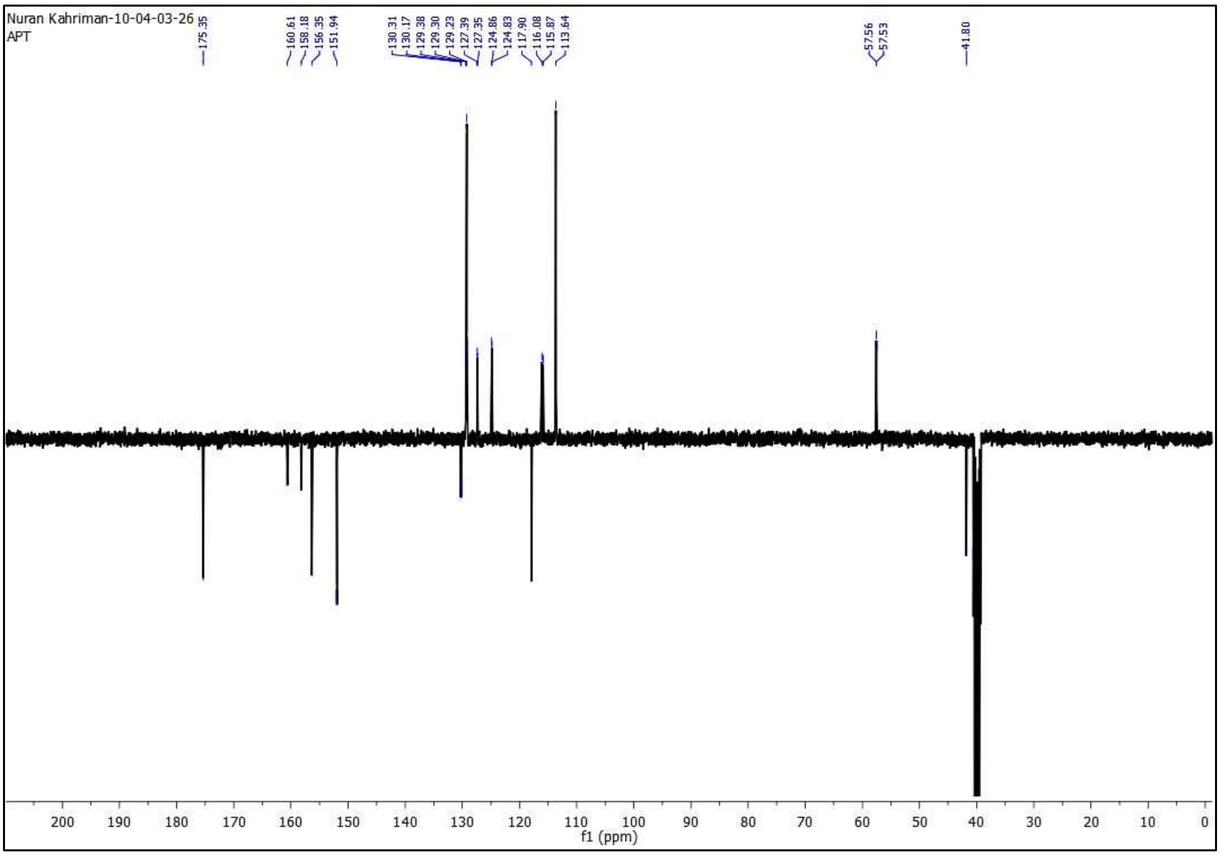

Figure S38.^{13}C-APT NMR spectrum of compound **10** (100 MHz, DMSO-d_6, ppm)

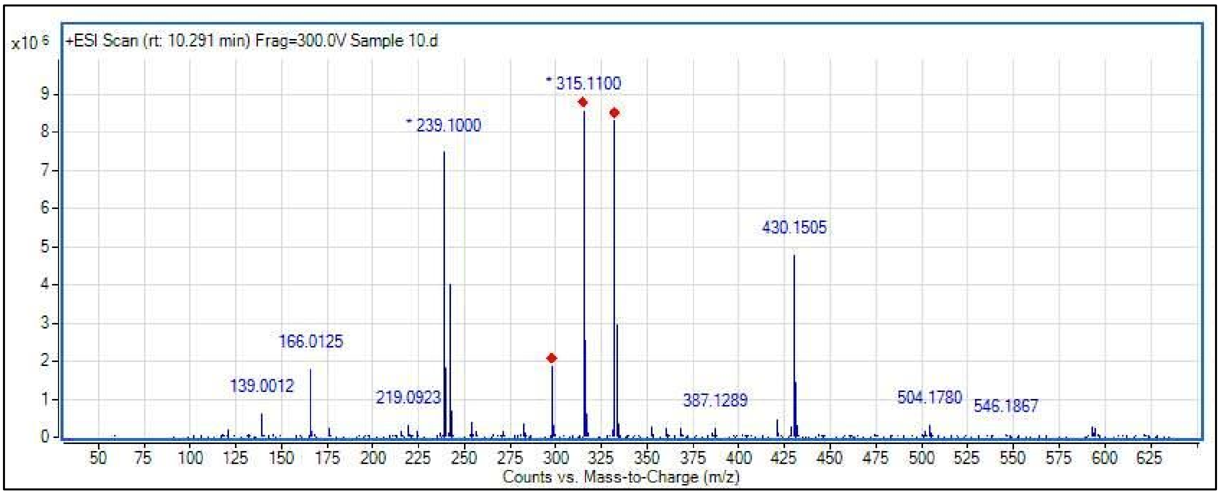

Figure S39. LC-Q-TOF-MS spectrum of compound **10**

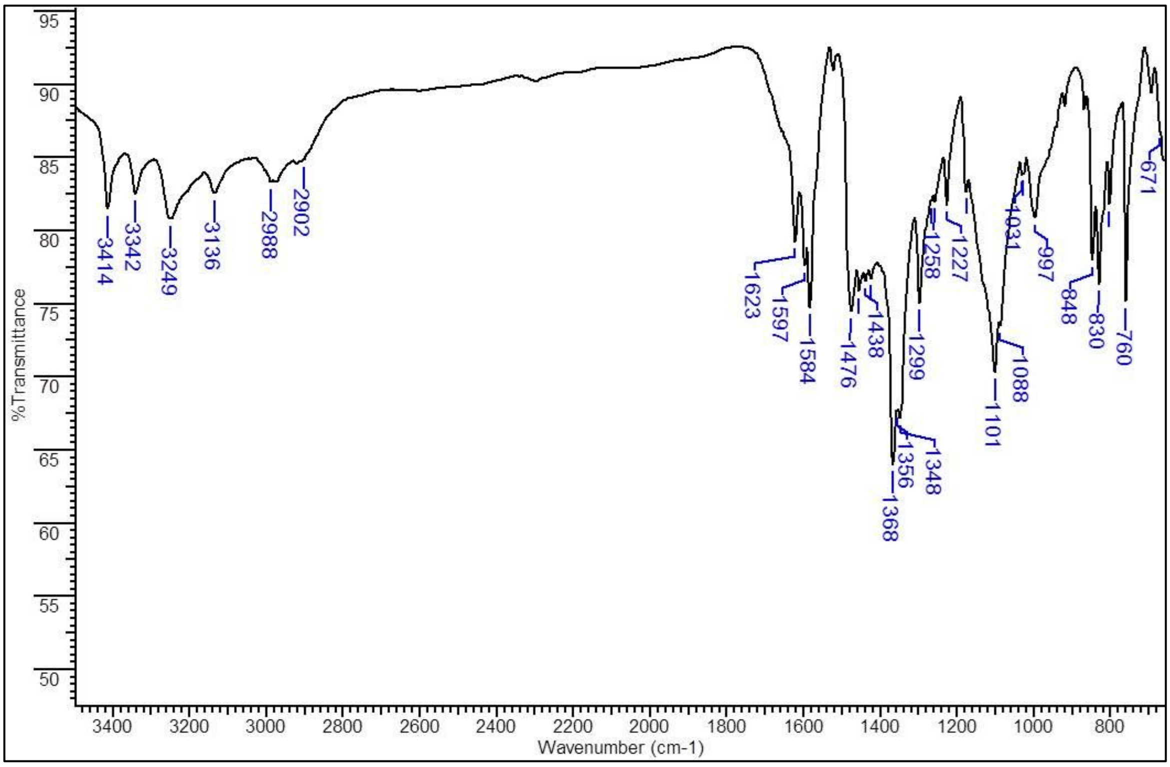

Figure S40. FT-IR spectrum of compound **10** (cm^{-1})

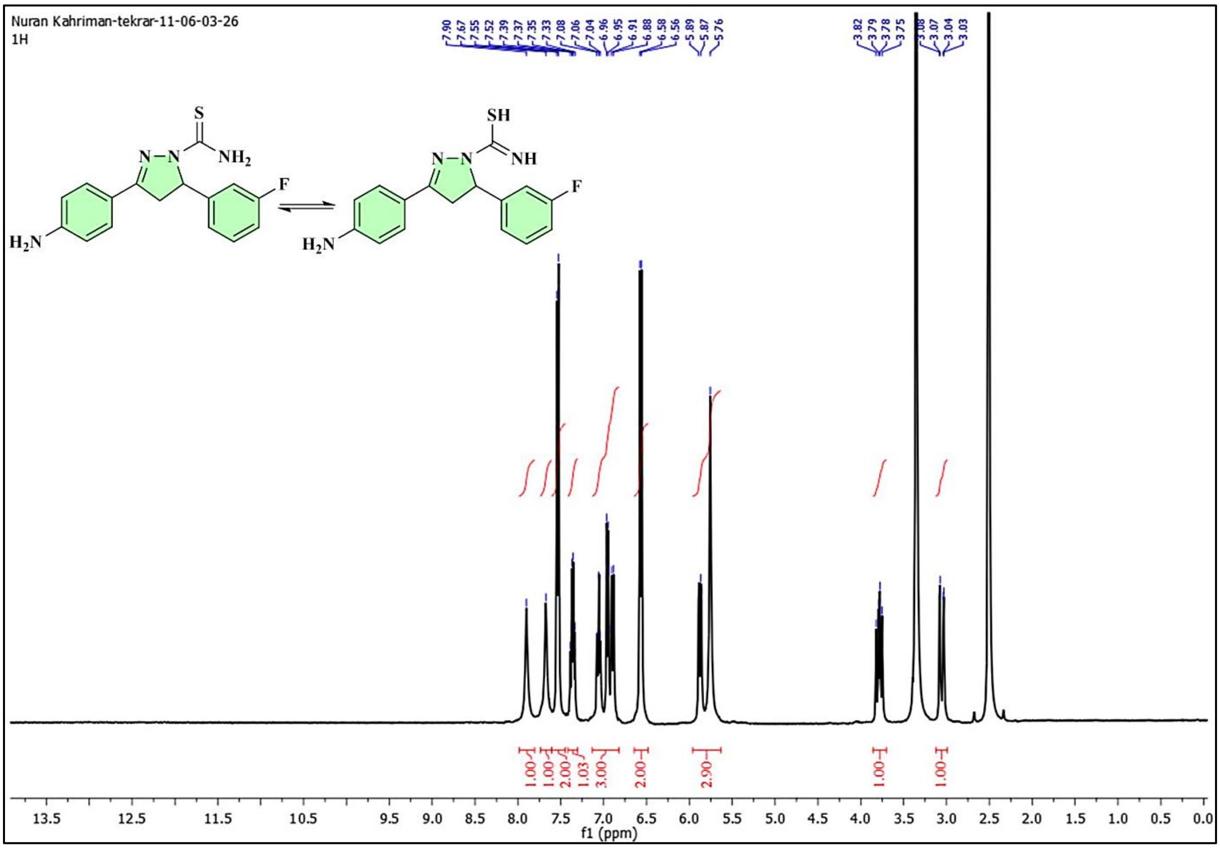

Figure S41. ^{1}H-NMR spectrum of compound **11** (400 MHz, DMSO-d_6, ppm)

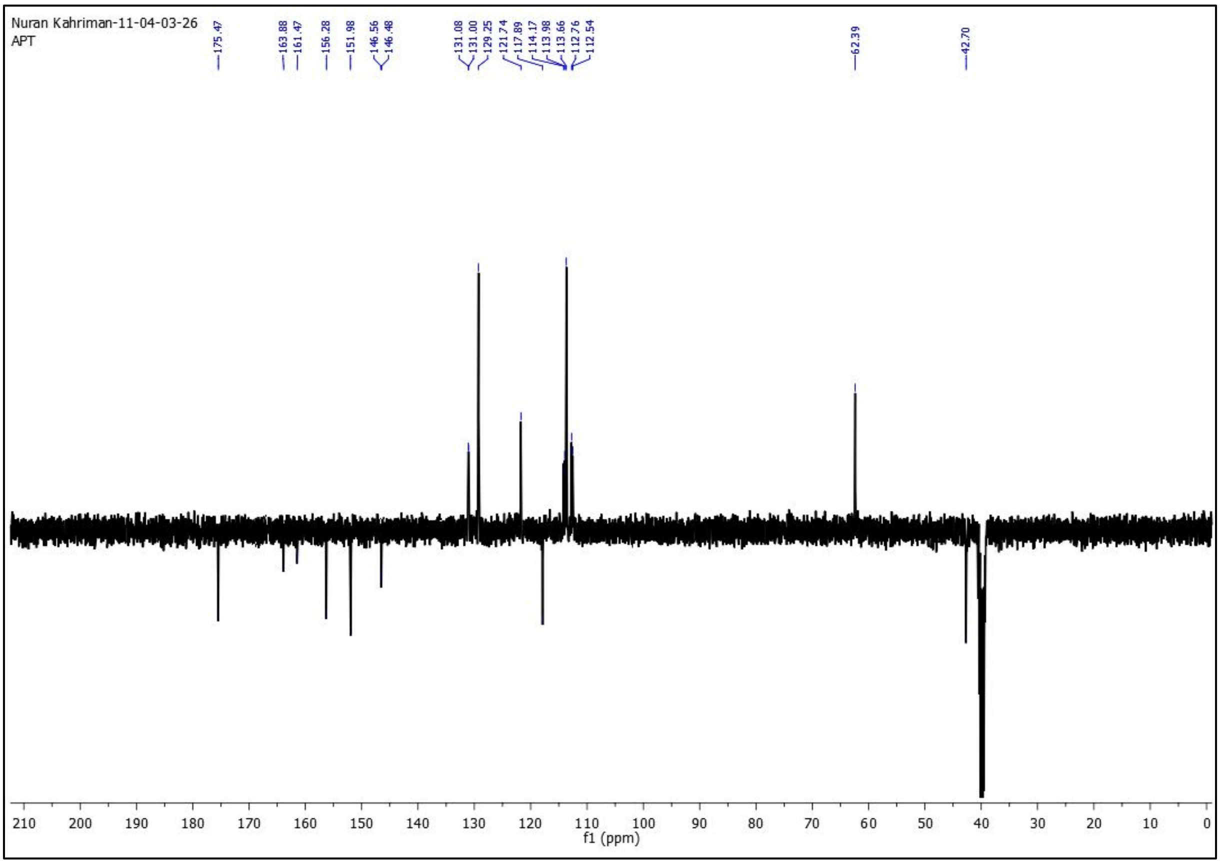

Figure S42.^{13}C-APT NMR spectrum of compound **11** (100 MHz, $CDCl_3$, ppm)

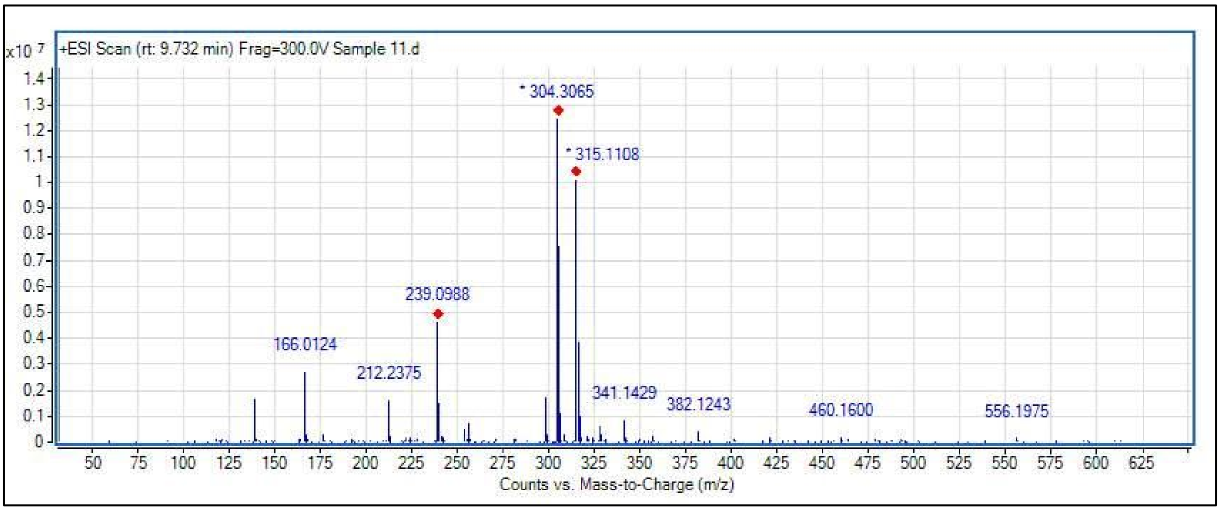

Figure S43. LC-Q-TOF-MS spectrum of compound **11**

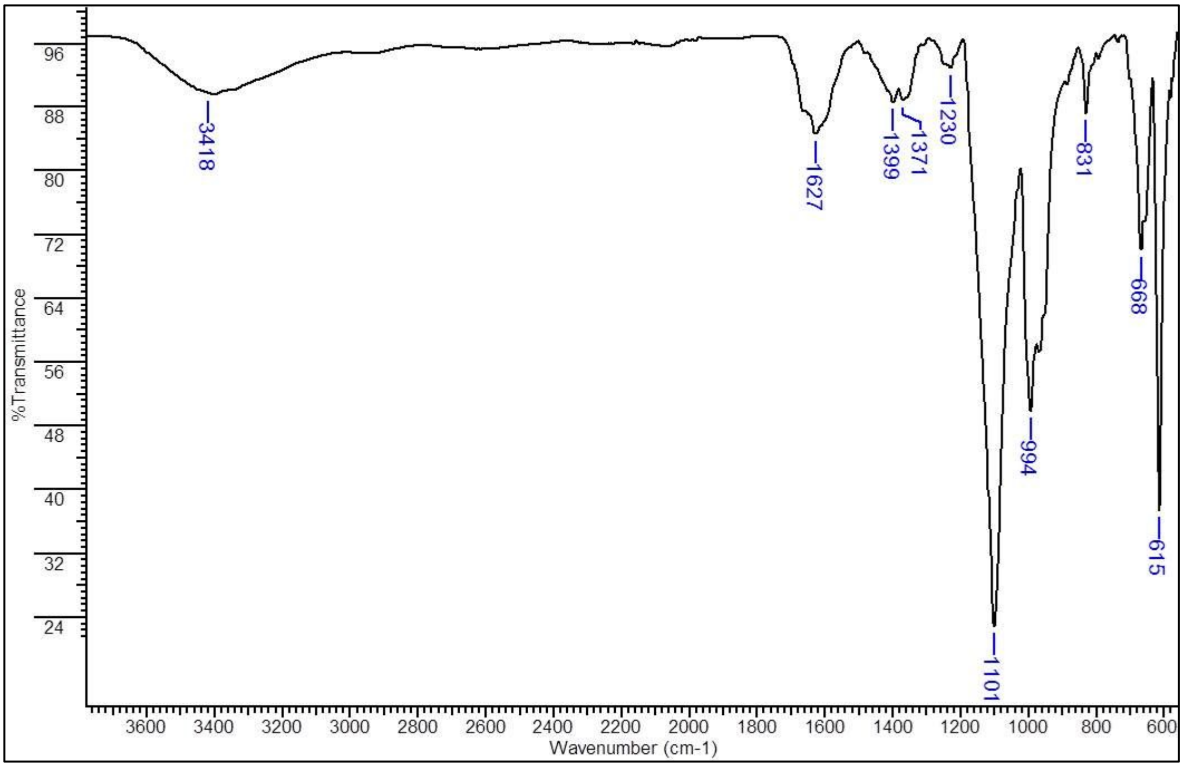

Figure S44. FT-IR spectrum of compound **11** (cm^{-1})

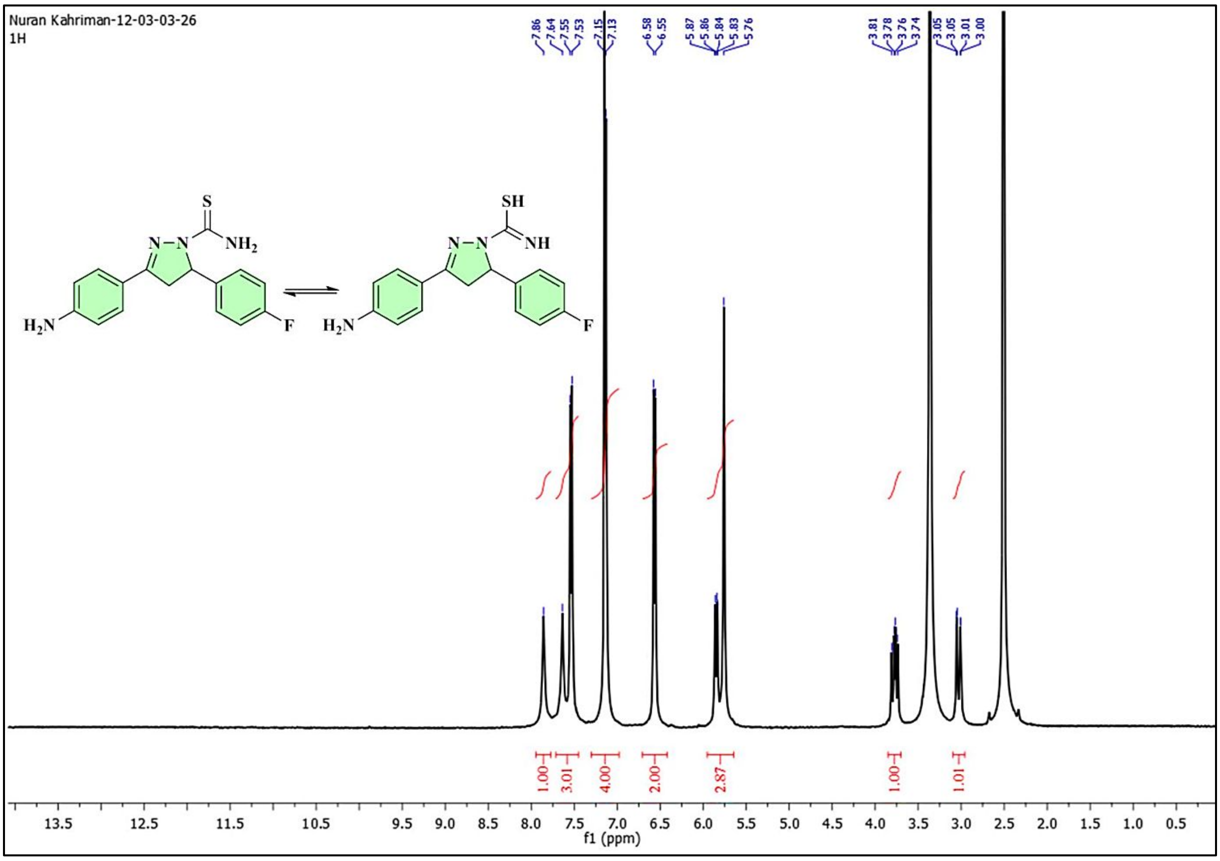

Figure S45. ^{1}H-NMR spectrum of compound **12** (400 MHz, DMSO-d_6, ppm)

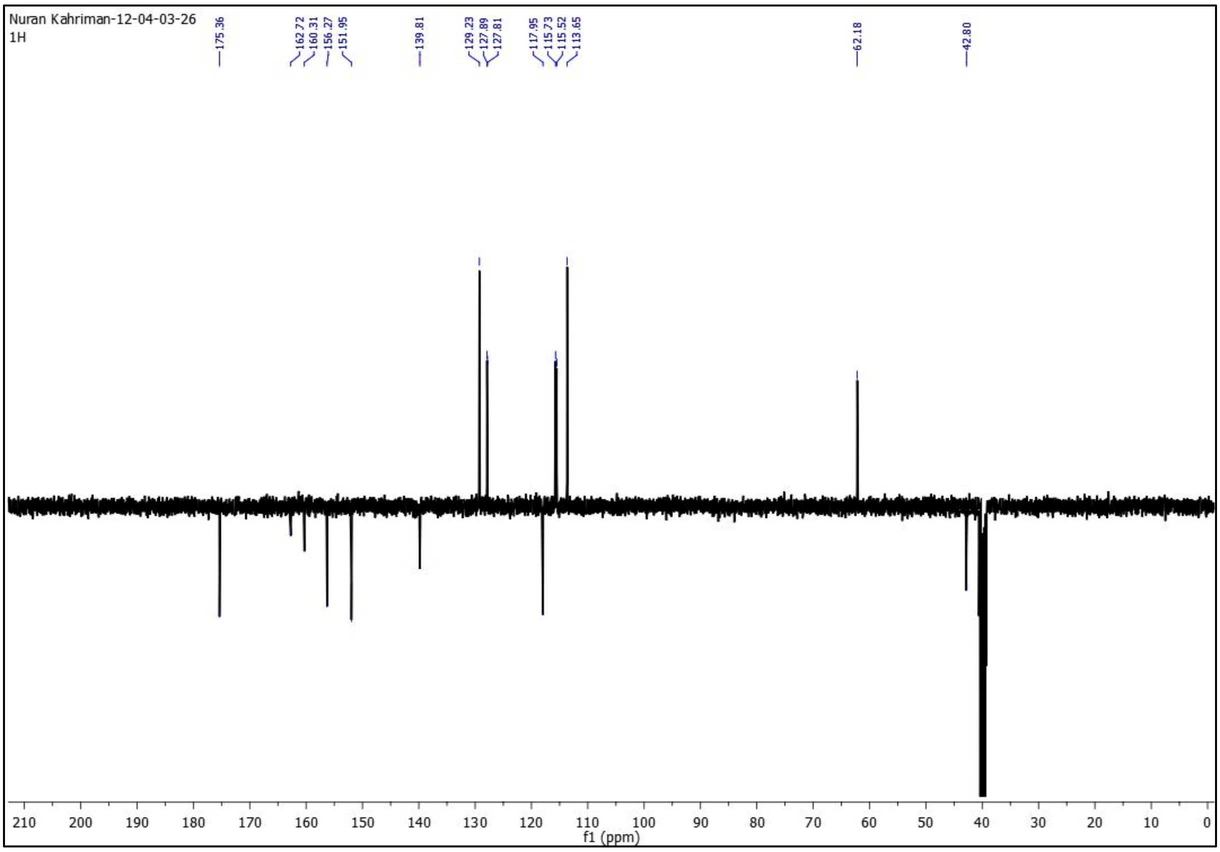

Figure S46. ^{13}C-APT NMR spectrum of compound **12** (100 MHz, DMSO-d_6, ppm)

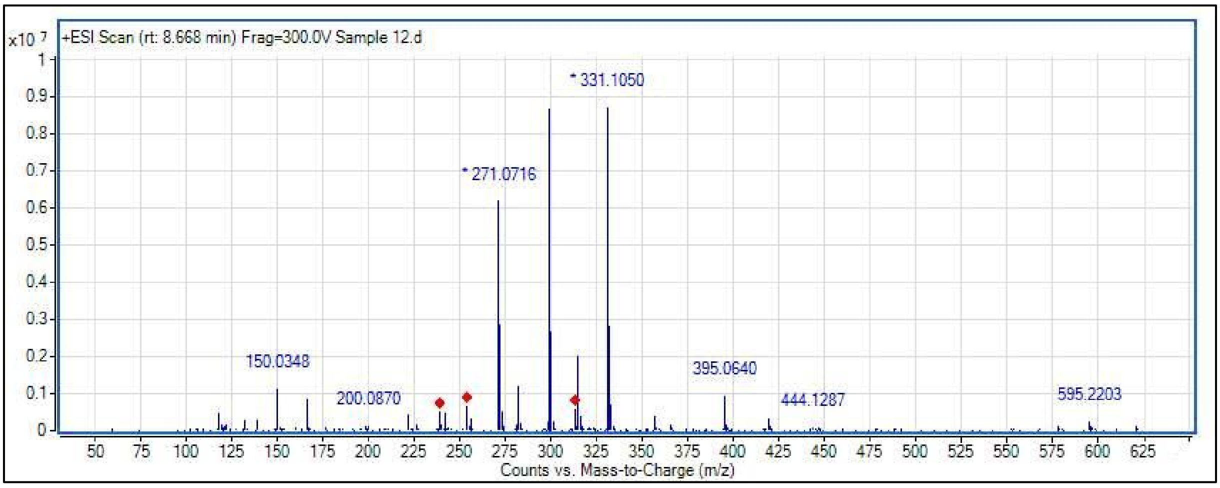

Figure S47. LC-Q-TOF-MS spectrum of compound **12**

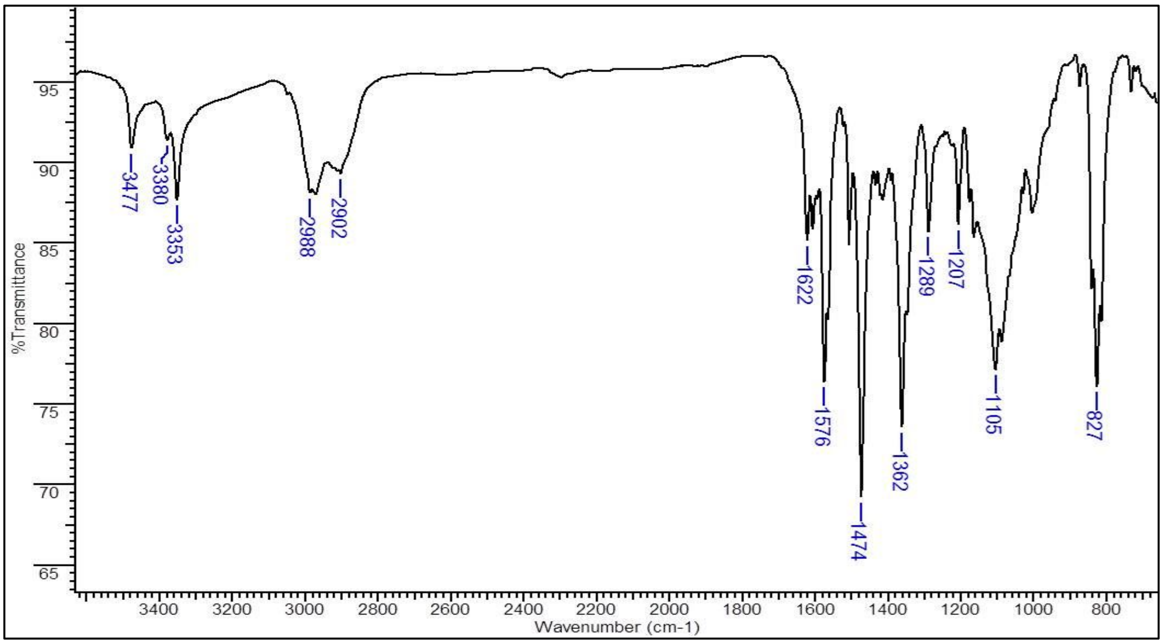

Figure S48. FT-IR spectrum of compound **12** (cm^{-1})

APPLICATION AND RESEARCH CENTER FOR MEDICINAL AND AROMATIC PLANTS

ANALYSIS RESULT NOTIFICATION FORM

Unit: KAL **Sample Number:TAB2636-TAB2646**

Due to a special request, the analysis results of the samples coded TAB2636 and TAB2646, which were analyzed in our Chemical Analysis Laboratory, are presented below.

Sample Code	Analysis Code	%Nitrogen	%Carbon	%Hydrojen	%Sulphur
TAB2635-1	TAB2635	5.817267532	74.63006287	5.029479694	0
TAB2636-2	TAB2636	5.816849556	74.65160583	5.020343626	0
TAB2637-3	TAB2637	5.808506371	74.68588348	5.015100479	0
TAB2638-4	TAB2638	5.811542557	74.67628754	5.016498356	0
TAB2639-5	TAB2639	5.812009758	74.68147797	5.016093159	0
TAB2640-6	TAB2640	5.813042736	74.67782013	5.012934246	0
TAB2641-7	TAB2641	17.82035645	61.13089111	4.817635365	10.22429333
TAB2642-8	TAB2642	17.82769272	61.13031350	4.810924053	10.21314499
TAB2643-9	TAB2643	17.81978733	61.12970749	4.814052315	10.20822289
TAB2644-10	TAB2644	17.82256591	61.13625244	4.826795769	10.21811661
TAB2645-11	TAB2645	17.82381386	61.12525192	4.817273502	10.22669647
TAB2646-12	TAB2646	17.82273178	61.12090506	4.817144756	10.21388474

***: Accredited analyses**

Abbreviations:
E: Evaluation, **C:** Compliant, **NC:** Non-Compliant, **NE:** Not Evaluated

Unit Sample Acceptance Date and Time: 03/03/2026 14:00
Analysis Start & End Date: 04/03/2026 -10/03/2026

Remarks:

Evaluation:

Full Name
KAL Unit
Unit Supervisor
Approval Date and Time:
11/03/2026 13:40
Abidin GÜMRÜKÇÜOĞLU

Received By
Full Name: Kemal Vehbi İMAMOĞLU
Date Received: 11/03/2026

F02.PR16/2/01.11.2025
Yayın Tarihi: 15.08.2023 1/1

Figure S49. Elemental analysis results for compounds **1-12**
